# Synthesis and Cytotoxic Activity of Novel C-23-Modified Asiatic Acid Derivatives

**DOI:** 10.3390/molecules25163709

**Published:** 2020-08-14

**Authors:** Yi-hong Lu, Ming-cang Chen, Fang Liu, Zhou Xu, Xiao-ting Tian, Yang Xie, Cheng-gang Huang

**Affiliations:** 1 Shanghai Institute of Materia Medica, Chinese Academy of Sciences, 501 Haike Road, Shanghai 201203, China; 201628012342013@simm.ac.cn (Y.-h.L.); baidulyac@126.com (M.-c.C.); lf86614@126.com (F.L.); xz_619@163.com (Z.X.); zhghydxtxt@163.com (X.-t.T.); 2 School of Pharmacy, University of Chinese Academy of Sciences, No. 19A Yuquan Road, Beijing 100049, China

**Keywords:** asiatic acid, modification at C-23, intramolecular hydrogen bonding, cytotoxic activity

## Abstract

We selectively oxidized the C-23 hydroxyl group in an asiatic acid (AA) derivative and then, for the first time with AA, modification of the C-23 carboxyl group was conducted to synthesize a series of new AA derivatives. The evaluation of their cytotoxic activities against two human cancer cell lines (SKOV-3 and HCT116) using the MTT assay in vitro revealed a distinctive structure activity relationship (SAR) associated with the intramolecular hydrogen bonding of the amide moiety at C-23. According to the established SAR, the cytotoxic activities of four promising compounds were then evaluated against MCF-7, A549, A2780, HepG2 and HL-60 cancer cell lines. Compound **10** had the best cytotoxic activity among all tested derivatives in the HL-60 cell line, giving IC_50_ = 0.47 μM, while showing no cytotoxic effect against human normal cells (HUVEC).

## 1. Introduction

**Figure 1:**
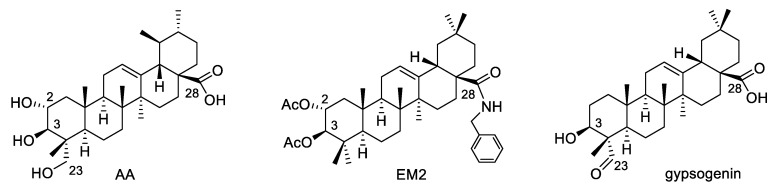
Chemical structure of asiatic acid (AA), EM2 and gypsogenin.

Natural products are a major source of lead compounds for new therapeutic agents [1,2]. In recent years, triterpenoids, as one of the largest classes of natural products, have been frequently reported for their remarkable bioactivities including anticancer effects; in particular, triterpenoid acids, such as oleanolic acid (OA) [3], asiatic acid (AA) (Figure 1) [4,5] and maslinic acid (MA) [6,7], have received increased attention.

Asiatic acid (AA, 2α,3β,23-trihydroxyurs-12-ene-28-oic acid), one of the active pentacyclic triterpenoid acids, is usually prepared from the hydrolysis of asiaticoside, which is extracted from the traditional Chinese medicine herb *Centella asiatica* [8]. The primarily usage of AA is for treating skin diseases [9,10]. In recent years, other noteworthy bioactivities have been reported, including hepatoprotective [11], antidiabetic [12], anti-inflammatory [13,14] and especially antitumor activity. AA inhibits proliferation of various cancer cell lines and induces apoptosis [4,5,15].

Previously, AA has been modified to improve its anticancer effects and other bioactivities. Li et al. reported that oxidation at C-11 to form α–β unsaturated ketone, and the introduction of anilines to form amide bond at C-28, could improve the anticancer activity in vitro [16]. Huang et al. synthesized AA based 1,2,3-triazole derivatives which were subjected to a cell-based NF-κB inhibition screening assay and MTT assay [17]. Huang et al. also synthesized AA derivatives containing α-aminophosphonate as antitumor agents [18]. Jing et al. introduced various amino acid esters to form amide bonds at C-28 [19]. Sommerwerk et al. modified C-28 with different quinolinyl amides [20]. Gonçalves et al. changed the A-ring framework to synthesize active compounds with a pentameric A-ring containing an α-β-unsaturated carbonyl group [21].

However, many modifications focused on the C-28 carboxyl group; the C-2, C-3 and C-23 hydroxyl groups have rarely been modified, except for the acetylation of all three hydroxyl groups due to the poor reaction selectivity of the three hydroxyl groups. In some studies, the C-3 and C-23 hydroxyl groups were protected as a ketal, and thus, modification at the 2α-hydroxy or further modification at the 3β,23-dihydroxy could be carried out [21,22,23,24,25,26]. Nevertheless, due to the poor reaction selectivity between the C-3 and C-23 hydroxyl groups, the respective modification of the C-3 and C-23 hydroxyl groups has rarely been reported [25].

Referring to other triterpenoid acids with a similar structure, an di-O-acetylated benzylamide maslinic acid derivative, named EM2 (Figure 1), exhibited rather low EC_50_ values (EC_50_ = 0.5 μM) against human ovarian cancer cells (A2780) [20,27]. Another natural triterpenoid acid, gypsogenin (Figure 1), is extracted from *Gypsophila* [28], and contains a natural C-23 aldehyde group, which inhibited several cancer cell lines [29]. Heller et al. conducted oxidation on the C-23 aldehyde group to synthesize 23-carboxyl and 23-methylester gypsogenin derivatives, which showed inhibition against several cancer cell lines [30]. Wu et al. synthesized bisamide gypsogenin derivatives at the C-23 and C-28 positions, which showed good antitumor activity. A 23-oicacid-28amide gypsogenin derivative gave IC_50_ = 2.2 μΜ for HepG-2 cell line [31].

Taking into account these good results, in this paper we introduced benzyl amine to form amide bonds at C-28, and conducted rather highly selective oxidation at the C-23 hydroxyl group to transform 23-hydroxy into aldehyde and then further into carboxyl functionalities. Afterwards, modification on the C-23 carboxyl group was conducted for the first time to synthesize a series of novel AA derivatives. The cytotoxic activities against ovarian (SKOV-3) and colon (HCT-116) cancer cell lines of these derivatives were evaluated using the MTT assay in vitro. According to the structure activity relationship (SAR) established on the cytotoxic activities against SKOV-3 and HCT-116 cells, the cytotoxic activities of four promising compounds were then evaluated against additional cancer cell lines (MCF-7, A549, A2780, HepG2 and HL-60).

## 2. Results and Discussion

### 2.1. Chemistry

**Scheme 1:**
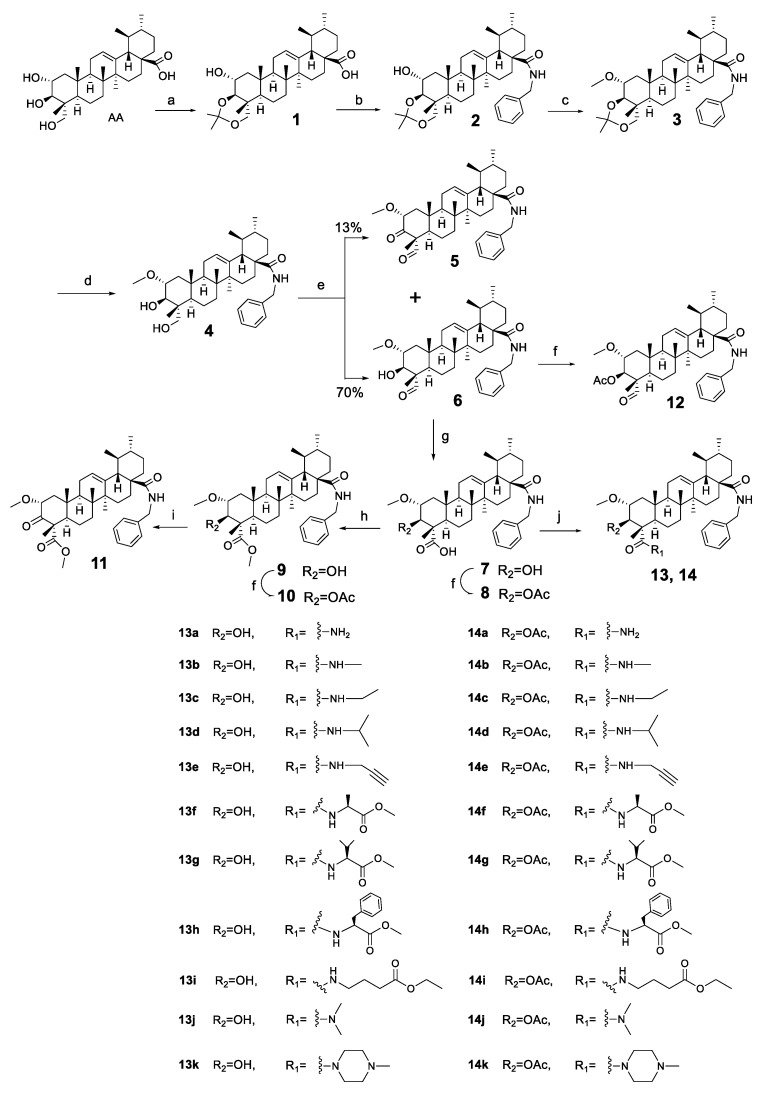
Reagents and conditions: (**a**) *p*-TsOH·H_2_O, 2,2-dimethoxypropane, dry acetone, rt, 12 h, 85%; (**b**) HATU, DIPEA, benzyl amine, dry DMF, 60 °C, 12 h, 62%; (**c**) NaH, CH_3_I, dry DMF, rt, 30 min, 97%; (**d**) 1M aq. HCl, rt, 30 min, 98%; (**e**) DMP, dry DCM, rt, 5: 13%, 6: 70%; (**f**) Ac_2_O, pyridine, rt, 12 h, 100%; (**g**) 2-methyl-2-butene, NaH_2_PO_4_, NaClO_2_, *t*-BuOH, H_2_O, rt, 1 h, 93%; (**h**) K_2_CO_3,_ CH_3_I, dry DMF, rt, 30 min, 89%; (**i**) PCC, DCM, rt, 12 h, 46%; (**j**) HOBt, EDCI·HCl, DIPEA, amine, dry DMF, 85 °C, 12 h, 45–84%.

The synthetic route of the newly synthesized AA derivatives is shown in Scheme 1. To protect the C-3 and C-23 hydroxyl groups, AA as the starting material was reacted with 2,2-dimethoxypropane to selectively form a six-membered ketal ring in the initial framework, affording crude product compound **1** in high yield. We introduced benzyl amine to form an amide bond at C-28, thereby obtaining compound **2**. After methylation at the 2α-hydroxy, the C-3 and C-23 hydroxyl groups were recovered in compound **4**. Critically, to obtain compound **6**, a low concentration DCM solution of Dess–Martin Periodinane (DMP) was added to the solution of compound **4** at an appropriately slow speed, because DMP prefers to oxidize the C-23 hydroxyl group, but the production of the side product, compound **5**, was inevitable. It should be noted that DMP gradually loses its efficacy at room temperature. We adapted a similar oxidation method carried out on gypsogenin [30] to further transform the 23-aldehyde into the 23-carboxyl and thereby, obtained compound **7** in 93% yield. Compound **9** was prepared by methylation of 23-carboxyl of compound **7** with CH_3_I. Then, oxidation of the C-3 hydroxyl group of compound **9** was carried out by employing pyridinium chlorochromate (PCC) to generate compound **11**. Compounds **13** and **14** were prepared by the introduction of various amines at the 23-carboxyl group in compounds **7** and **8** in yields between 45–84%. The structures of all new compounds were fully confirmed by ^1^H-NMR, ^13^C-NMR and high resolution mass spectrometry (HRMS).

### 2.2. Biology

To establish a SAR of the C-3 (R_2_) and C-23 (R_1_) of the AA derivatives, new compounds were subjected to MTT assays to evaluate their in-vitro antiproliferative activities against ovarian (SKOV-3) and colon (HCT-116) cancer cell lines, the IC_50_ values (the concentration that inhibits 50% of cell growth) of which are summarized in Table 1. Doxorubicin was used as the positive drug.

For compounds **13f**–**i**, with the introduction of various amino acid esters at C-23, their cytotoxic activities against the HCT116 cell line decreased progressively along with that of the larger group at C-23. In addition, the cytotoxic activities of compound **13f** against the SKOV-3 cell line improved considerably, and yet compound **13h** was not active. These combined results suggested that the smaller amide moiety at C-23 led to increased cytotoxic activity.

For the modification at C-3, compounds **9** and **10** were 8- and 23-fold more active, respectively, than AA against HCT116 cell line, and compound **9** was 3-fold more active than AA against SKOV-3 cell line, while the 3β-hydroxy oxidation product, compound **11,** lost activity towards both cell lines. In addition, compounds **6** and **12** were both much more active than compound **5** against the HCT116 cell line. These results indicated that the carbonyl moiety at C-3 decreased the cytotoxicity.

We studied the intramolecular hydrogen bonds of the 23-amide AA derivatives via exhaustive NMR characterization (Table 2). The assignments of C-2, C-3, C-23 and NH-23 of compounds **13b** and **14b** were unambiguously achieved by a combination of ^1^H, ^13^C, DEPT, HSQC and HMBC experiments (see the Supplementary Materials). The assignments of compounds **13a**, **c**–**j** and **14a**, **c**–**j** were then accomplished by analogy. The effect of intermolecular hydrogen bonding depends on the concentration of the NMR sample. In addition, the chemical shift is badly influenced by the intermolecular hydrogen bonding in the ^1^H-NMR experiment, while it is barely influenced in the ^13^C-NMR experiment. To minimize the effect of intermolecular hydrogen bonding in the ^1^H-NMR experiment, NMR titration (see the Supplementary Information) was conducted in compounds **13b** and **14b** to select the proper concentration, and then the ^1^H-NMR signals of NH-23 of compounds **13** and **14** were all recorded at the same concentration (4.85 mM). The ^13^C-NMR signals of C-2, C-3 and C-23 were recorded without the limitation of concentration.

**Figure 2:**
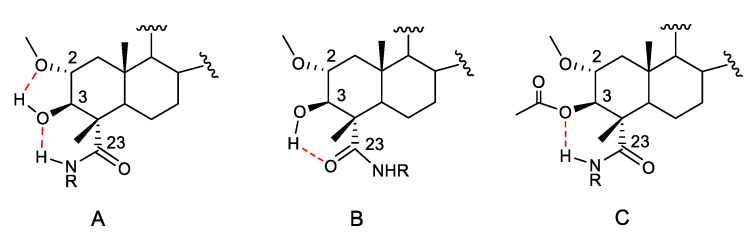
Three possible intramolecular hydrogen bonding modes for compounds **13** or **14**. Mode (**A**): the hydrogen bonds connecting 3β-OH donor with 2α-OCH_3_ acceptor and connecting NH-23 donor with 3β-OH acceptor; Mode (**B**): the hydrogen bond connecting 3β-OH donor with 23-carbonyl acceptor; Mode (**C**): the hydrogen bond connecting NH-23 donor with 3β-acetoxy acceptor.

According to Table 2, after the acetylation of 3β-hydroxy, the chemical shift at C-2, C-3, C-23 and NH-23 had changed because the loss of 3β-OH donor influenced or deprived the intramolecular hydrogen bonds. Thus, we could deduce the hydrogen bond from the change of the chemical shift (Table 2). Specifically, we could deduce the hydrogen bond connecting 3β-OH donor with 2α-OCH_3_ acceptor from the change of the chemical shift at C-2 and C-3. We could deduce the hydrogen bond connecting 3β-OH donor with 23-carbonyl acceptor from the change of the chemical shift at C-23. Additionally, we could deduce the hydrogen bond connecting NH-23 donor with 3β-OH acceptor from the change of the chemical shift at NH-23. Further, the chemical shifts of two amide hydrogen atoms at NH_2_-23 in compound **14a** were apparently different, which revealed the hydrogen bond connecting NH-23 donor with 3β-acetoxy acceptor. By analogy, it was possible to form the hydrogen bonds in 3β-acetoxy derivatives **14**. To sum up, the hydrogen bonding modes A and B (Figure 2) for 3β-hydroxy derivatives **13** were supposed to exist in the solvent at the same time, and mode C for 3β-acetoxy derivatives **14** may exist.

Surprisingly, for compounds **13a**–**i** and **14a**–**i,** we found that the 23-amide AA derivatives which showed rather good cytotoxic activities, could fix the amide hydrogen atom in the AA skeleton through intramolecular hydrogen bonding, no matter what the mode (A, B or C). Comparatively, the 23-amide AA derivatives which were not active, were unable to fix the amide hydrogen atom in the AA skeleton. Specifically, for compounds **13**, according to Table 2, the amide hydrogen atom at C-23 of **13b**–**h** could be fixed in the AA skeleton through mode A or B, the amide hydrogen atom of **13i** could be fixed through mode B, and the amide hydrogen atom of **13a** could be fixed through mode A. After the acetylation of 3β-hydroxy, the cytotoxic activities of compounds **14** against HCT116 cell line decreased progressively along with that of the larger group at C-23, and **14c**–**i** even lost its cytotoxic activities, probably because the steric hindrance of the amide moiety at C-23 blocked the intramolecular hydrogen bond in mode C. Therefore, compounds **13a**–**g, i** and **14a** which fixed the amide hydrogen atom in the AA skeleton, exhibited increased cytotoxic activity against HCT116 cell line. Similarly, compounds **13e**–**f** and **14a** showed rather good cytotoxic activities against the SKOV-3 cell line. Comparatively, compounds **14c**–**i**, which were unable to fix the amide hydrogen atom in the AA skeleton, were not active in either cell line. Additionally, compounds **9**, **10**, **13j** and **14j** without the amide hydrogen atom at C-23 exhibited a considerable improvement in the cytotoxic activities against the HCT116 cell line. To summarize, the cytotoxicity results suggested that the freely rotating amide hydrogen atom at C-23 led to the loss of cytotoxic activity.

Hence, the cytotoxic activities of compounds **10**, **13k**, **14j** and **14k** without any amide hydrogen atom at C-23 were evaluated in a panel of additional five cancer cell lines (MCF-7, A549, A2780, HepG2 and HL-60) using the MTT assay (Table 3). 3β-acetoxy derivative **14k** was more active than 3β-hydroxy derivative **13k** against A549, A2780, HL-60 and MCF-7 cell lines, and was commensurately active with **14k** against HepG2 cell lines. Combined with the results against the HCT116 cell line shown in Table 1, 3β-acetoxy derivatives **10** and **14j** exhibited increased activity compared to the corresponding 3β-hydroxy derivatives **9** and **13j**. These combined results suggested that 3β-acetoxy moiety improved the cytotoxic activity, in the case of a lack of amide hydrogen atoms at C-23.

**Figure 3:**
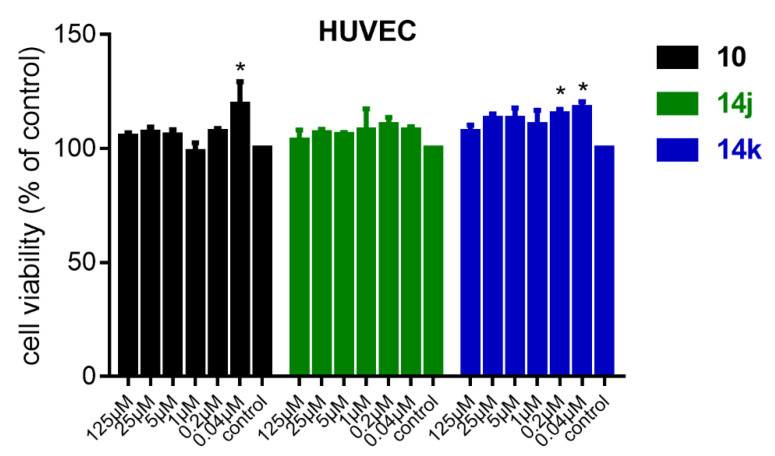
Cell viability of compounds **10**, **14j** and **14k** on the human normal cell line (HUVEC). * *p* < 0.05 vs. control.

Additionally, the cytotoxic activities of compounds **10**, **14j** and **14k** were evaluated against one human normal cell line (HUVEC) using CCK-8 assay (Table 3). Compounds **10**, **14j** and **14k** at the concentrations of 125, 25, 5, 1, 0.2, 0.04 μM all showed no cytotoxic effect on HUVEC cell viability (Figure 3). These results indicated that the cytotoxicity of compounds **10**, **14j** and **14k** against cancer cells was much higher than that against HUVEC normal cells.

## 3. Materials and Methods

### 3.1. Chemistry

#### 3.1.1. General

Asiatic acid was purchased from Xi’an Shouhe Biotechnology Co., Ltd. All reagents and solvents were obtained from Sinopharm. IR spectra were recorded on a THERMO IS5. NMR spectra (^1^H, ^13^C, DEPT, HSQC and HMBC) were recorded on a Bruker Ascend-400 400 MHz or Bruker Ascend-500 500 MHz at room temperature. HRMS were recorded on an Agilent 6530-Q-TOF mass spectrometer equipped with an Agilent 1260-HPLC.

#### 3.1.2. α-Hydroxy-3β,23-Isopropylidenedioxy-Urs-12-Ene-28-Oic Acid (**1**)

Under a nitrogen atmosphere, AA (480 mg, 1 mmol) was dissolved in dry acetone (50 mL). 2,2-dimethoxypropane (150 μL, 1.2 mmol) and TsOH·H_2_O (17 mg, 0.1 mmol) was added, stirring at room temperature overnight. Evaporated 2,2-dimethoxypropane and acetone, neutralized the reaction mixture with a saturated solution of sodium bicarbonate and extracted the solution with DCM (dichloromethane). The organic phase was washed with brine, dried over Na_2_SO_4_ and concentrated to dryness under vacuum to obtain the crude product **1** as a white solid (449 mg, 85%). IR (CH_2_Cl_2_, cm^−1^) ν_max_: 1033, 1190, 1391, 1454, 1552, 1648, 1689, 2871, 2939, 3199, and 3466 [22]. ^1^H-NMR (400 MHz, CDCl_3_) δ 5.25 (t, *J* = 3.6 Hz, 1H, H-12), 3.81 (td, *J* = 10.2, 4.5 Hz, 1H, H-2), 3.58–3.44 (m, 2H, H-23), 3.34 (d, *J* = 9.6 Hz, 1H, H-3), 1.27 (d, *J* = 2.2 Hz, 3H), 1.11 (s, 3H), 1.07 (s, 3H), 1.06 (s, 3H), 0.87 (d, *J* = 6.4 Hz, 3H), 0.76 (s, 3H). ^13^C-NMR (101 MHz, CDCl_3_) δ 183.63 (C-28), 137.85 (C-13), 125.45 (C-12), 99.65 (3β,23-ketal), 82.10 (C-3), 72.67 (C-23), 65.26 (C-2), 52.44, 51.38, 47.87, 47.57, 46.49, 41.94, 39.55, 39.01, 38.79, 38.01, 36.92, 36.66, 32.30, 30.57, 29.74, 27.92, 23.97, 23.67, 23.15, 21.20, 19.43, 17.96, 17.50, 16.99, 16.95, 13.49. HRMS (ESI) observed C_33_H_51_O_5_ 527.3742 (M-H^−^) requires 527.3742.

#### 3.1.3. α-Hydroxy-3β,23-Isopropylidenedioxy-Urs-12-Ene-28-Benzylamide (**2**)

To a solution of compound **1** (1.1 g, 2 mmol) in dry DMF (30 mL), HATU (1.17 g, 3 mmol), benzyl amine (0.5 mL, 4 mmol) and DIPEA (65 μL, 4 mmol) was added, stirring at 60 °C overnight. The reaction mixture was poured into the brine, and extracted with ethyl acetate (100 mL × 3). The organic phase was washed with brine (200 mL × 3), dried over Na_2_SO_4_ and concentrated to dryness under vacuum. The crude product was purified by column chromatography over silica gel ((200–300 mesh) using hexane/AcOEt (5:2) to afford compound **2** as a white solid (766 mg, 62%). IR (CH_2_Cl_2_, cm^−1^) ν_max_: 3367, 2920 (C=C-H), 1639 (C=O), 1520 (C=C). ^1^H-NMR (400 MHz, CDCl_3_) δ 6.18 (m, 1H, NH-28), 5.24 (t, *J* = 3.5 Hz, 1H, C-12), 4.51 (dd, *J* = 14.5, 5.9 Hz, 1H, CH_2_-N-28), 4.15 (dd, *J* = 14.8, 4.7 Hz, 1H, CH_2_-N-28), 4.10 (q, *J* = 7.2 Hz, 1H), 3.79 (td, *J* = 10.3, 4.5 Hz, 1H, H-2), 3.59–3.44 (m, 2H, H-23), 3.32 (d, *J* = 9.6 Hz, 1H, H-3), 2.06 (s, 1H), 1.12 (s, 3H), 1.09 (s, 3H), 1.01 (s, 3H), 0.86 (d, *J* = 6.5 Hz, 3H), 0.69 (s, 3H). ^13^C-NMR (101 MHz, CDCl_3_) δ 177.80 (C-28), 139.76, 138.31, 128.67, 127.89, 127.41, 125.39 (C-12), 99.60 (3β,23-ketal), 82.06 (C-3), 72.64 (C-23), 65.21 (C-2), 53.96, 51.34, 47.72, 47.56, 46.53, 43.68, 42.50, 39.75, 39.60, 39.06, 37.89, 37.20, 36.89, 32.19, 30.86, 29.75, 27.83, 24.80, 23.33, 23.18, 21.21, 19.41, 17.92, 17.47, 17.19, 16.90, 13.51. HRMS (ESI) observed C_40_H_60_NO_4_ 618.4518 (MH^+^) requires 618.4517.

#### 3.1.4. α-Methoxy-3β,23-Isopropylidenedioxy-Urs-12-Ene-28-Benzylamide (**3**)

Under a nitrogen atmosphere, compound **2** (3.3 g, 5.3 mmol) was dissolved in dry DMF (20 mL), and NaH (120 mg, 8 mmol) was added at 0 °C. After stirring for 20 min, CH_3_I (1.6 mL 25 mmol) was added in room temperature and stirring was continued for 30 min. The reaction mixture was poured into the brine, and extracted with ethyl acetate (100 mL × 3). The organic phase was washed with brine (200 mL × 3), dried over Na_2_SO_4_ and concentrated to dryness under vacuum to obtain the crude product **3** as a white solid (3.25 g, 97%). IR (CH_2_Cl_2_, cm^−1^) ν_max_: 3376, 2921 (C=C-H), 1640 (C=O), 1522 (C=C). ^1^H-NMR (400 MHz, CDCl_3_) δ 6.17 (t, *J* = 5.3 Hz, 1H, NH-28), 5.22 (t, *J* = 3.5 Hz, 1H, H-12), 4.52 (dd, *J* = 14.5, 5.9 Hz, 1H, CH_2_-N-28), 4.18 (dd, *J* = 14.5, 4.5 Hz, 1H, CH_2_-N-28), 3.54–3.43 (m, 3H, H-23 and H-3), 3.41 (s, 3H, OCH_3_-2), 3.36 (td, *J* = 10.2, 4.2 Hz, 1H, H-2), 2.07 (dd, *J* = 12.6, 4.4 Hz, 1H), 1.09 (d, *J* = 1.6 Hz, 6H), 0.99 (s, 3H), 0.67 (s, 3H). ^13^C-NMR (101 MHz, CDCl_3_) δ 177.70 (C-28), 139.75, 138.35, 128.63, 127.89, 127.37, 125.33 (C-12), 99.16 (3β,23-ketal), 81.44 (C-3), 74.32 (C-2), 72.62 (C-23), 57.63 (OCH_3_-2), 53.92, 51.00, 47.69, 47.58, 44.91, 43.66, 42.47, 39.72, 39.55, 39.05, 37.82, 37.35, 37.22, 32.20, 30.87, 29.88, 27.83, 26.91, 24.79, 23.31, 23.18, 21.22, 19.27, 17.87, 17.35, 17.18, 16.87, 13.73. HRMS (ESI) observed C_41_H_62_NO_4_ 632.4673 (MH^+^) requires 632.4673.

#### 3.1.5. α-Methoxy-3β,23-Dihydroxy-Urs-12-Ene-28-Benzylamide (**4**)

Compound **3** (2.5 g, 4 mmol) was stirred with 40 mL of dilute aqueous HCl (1 M) for 30 min. The reaction mixture was neutralized with a saturated solution of sodium bicarbonate and extracted with DCM. The organic phase was washed with brine, dried over Na_2_SO_4_ and concentrated to dryness under vacuum to obtain the crude product. The crude product was purified by column chromatography over silica gel ((200–300 mesh) using hexane/AcOEt (3:2) to afford compound 4 as a white solid (2.32 g, 98%). IR (CH_2_Cl_2_, cm^−1^) ν_max_: 3421, 2920 (C=C-H), 1637 (C=O), 1522 (C=C). ^1^H-NMR (400 MHz, CDCl_3_) δ 6.24–6.09 (m, 1H, NH-28), 5.24 (t, *J* = 3.5 Hz, 1H, H-12), 4.55 (dd, *J* = 14.5, 5.9 Hz, 1H, CH_2_-N-28), 4.20 (dd, *J* = 14.5, 4.5 Hz, 1H,) CH_2_-N-28, 4.13 (q, *J* = 7.1 Hz, 1H), 3.64 (d, *J* = 10.8 Hz, 1H, H-23), 3.46 (d, *J* = 9.5 Hz, 1H, H-23), 3.40 (s, 3H, OCH_3_-2), 3.39 (dd, 1H, H-3), 3.30 (ddd, *J* = 11.2, 9.5, 4.3 Hz, 1H, H-2), 2.75 (br s, 3H), 2.11 (dd, *J* = 12.4, 4.3 Hz, 1H), 2.06 (s, 1H), 1.10 (s, 3H), 1.00 (s, 3H), 0.88 (s, 3H), 0.86 (d, *J* = 6.5 Hz, 3H), 0.71 (s, 3H). ^13^C-NMR (101 MHz, CDCl_3_) δ 177.86 (C-28), 139.97, 138.32, 128.67, 127.92, 127.41, 125.33 (C-12), 78.36 (C-2), 78.36 (C-3), 69.60 (C-23), 56.58 (OCH_3_-2), 53.93, 48.34, 47.73, 47.42, 43.71, 42.57, 42.36, 41.93, 39.70, 39.58, 39.06, 37.88, 37.24, 32.42, 30.86, 27.80, 24.83, 23.42, 23.34, 21.21, 18.05, 17.23, 17.19, 17.07, 12.79. HRMS (ESI) observed C_38_H_58_NO_4_ 592.4373 (MH^+^) requires 592.4360.

#### 3.1.6. α-Methoxy-3,23-Dioxo-Urs-12-Ene-28-Benzylamide (**5**)

Fresh DCM (50 mL × 3) suspension of Dess-Martin Periodinane (283 mg × 3, 2 mmol) added dropwise in three separate times to compound **4** (1 g, 1.7 mmol) dissolved in dry DCM (20 mL), until starting material almost consumed. The reaction mixture was poured into aqueous solution of sodium thiosulfate. The organic phase was washed with brine, followed by dried over anhydrous Na_2_SO_4_ and concentrated to dryness under vacuum. The crude product was chromatographed over silica gel (200–300 mesh) using hexane/AcOEt (5:1) to give compound **5** as a white solid (130 mg, 13%). IR (CH_2_Cl_2_, cm^−1^) ν_max_: 3440, 2919 (C=C-H), 1737 (C=O), 1705 (C=O), 1645 (C=O), 1517 (C=C). ^1^H-NMR (400 MHz, CDCl_3_) δ 9.43 (s, 1H, H-23), 6.11 (t, *J* = 5.4 Hz, 1H, NH-28), 5.26 (t, *J* = 3.6 Hz, 1H, H-12), 4.54 (dd, *J* = 14.5, 5.8 Hz, 1H, CH_2_-N-28), 4.23 (dd, *J* = 14.6, 4.7 Hz, 1H, CH_2_-N-28), 3.93 (dd, *J* = 10.1, 6.6 Hz, 1H, H-2), 3.39 (s, 3H, OCH_3_-2), 2.34 (dd, *J* = 13.2, 6.7 Hz, 1H), 1.31 (s, 3H), 1.19 (s, 3H), 1.13 (s, 3H), 0.75 (s, 3H). ^13^C-NMR (101 MHz, CDCl_3_) δ 209.38 (C-3), 199.45 (C-23), 177.57 (C-28), 140.05, 138.36, 128.68, 127.92, 127.43, 124.82 (C-12), 78.87 (C-2), 63.33 (OCH_3_-2), 58.36 (C-4), 53.97, 48.81, 47.74, 47.11, 43.70, 42.64, 39.71, 39.55, 39.05, 37.28, 37.02, 31.74, 30.83, 27.81, 24.75, 23.47, 23.34, 21.17, 20.01, 17.20, 17.17, 17.00, 14.21. HRMS (ESI) observed C_38_H_54_NO_4_ 588.4060 (MH^+^) requires 588.4047.

#### 3.1.7. α-Methoxy-3β-Hydroxy-23-Oxo-Urs-12-Ene-28-Benzylamide (**6**)

Following the procedure given for **5**, compound **6** (701 mg, 70%) was obtained as a white solid (silica gel, hexane/AcOEt, 5:2). IR (CH_2_Cl_2_, cm^−1^) ν_max_: 3420, 2918 (C=C-H), 1730 (C=O), 1646 (C=O), 1521 (C=C). ^1^H-NMR (400 MHz, CDCl_3_) δ 9.35 (s, 1H, H-23), 6.14 (t, *J* = 5.3 Hz, 1H, NH-28), 5.24 (t, *J* = 3.4 Hz, 1H, H-12), 4.51 (dd, *J* = 14.5, 5.8 Hz, 1H, CH_2_-N-28), 4.21 (dd, *J* = 14.5, 4.6 Hz, 1H, CH_2_-N-28), 3.59 (d, *J* = 9.5 Hz, 1H, H-3), 3.40 (s, 3H, OCH_3_-2), 3.34 (ddd, *J* = 11.0, 9.4, 4.3 Hz, 1H, H-2), 2.16 (dd, *J* = 12.6, 4.3 Hz, 1H), 1.12 (s, 3H), 1.11 (s, 3H), 1.01 (s, 3H), 0.69 (s, 3H). ^13^C-NMR (101 MHz, CDCl_3_) δ 205.41 (C-23), 177.65 (C-28), 139.94, 138.34, 128.66, 127.91, 127.40, 125.03 (C-12), 77.87 (C-3), 75.30 (C-2), 56.75 (OCH_3_-2), 54.75 (C-4), 53.87, 47.69, 47.56, 47.42, 43.69, 42.57, 41.92, 39.67, 39.62, 39.04, 37.96, 37.25, 32.07, 30.84, 27.75, 24.75, 23.41, 23.34, 21.20, 19.88, 17.19, 17.04, 10.17. HRMS (ESI) observed C_38_H_56_NO_4_ 590.4188 (MH^+^) requires 590.4204.

#### 3.1.8. α-Methoxy-3β-Hydroxy-Urs-12-Ene-28-Benzylamide-23-Oic Acid (**7**)

Compound **6** (1 g, 1.7 mmol) was dissolved in t-BuOH (12 mL) and water (4 mL), and added 2-methyl-2-butene (1 mL), sodium dihydrogen phosphate (200 mg, 5.1 mmol) and sodium chlorite (460 mg, 5.1 mmol). After stirring at room temperature for 1 h, the reaction mixture was added aqueous solution of sodium thiosulfate, and extracted with ethyl acetate (100 mL × 3). The organic phase washed with brine followed by dried over anhydrous Na_2_SO_4_ and concentrated to dryness under vacuum. The crude product was chromatographed over silica gel (200–300 mesh) using hexane/AcOEt (1:1) to afford **7** as a white solid (957 mg, 93%). IR (CH_2_Cl_2_, cm^−1^) ν_max_: 3420, 2922 (C=C-H), 1715 (C=O), 1635 (C=O), 1525 (C=C). ^1^H-NMR (400 MHz, CDCl_3_) δ 6.19 (t, *J* = 5.3 Hz, 1H, NH-28), 5.23 (d, *J* = 3.6 Hz, 1H, H-12), 4.54 (dd, *J* = 14.5, 5.7 Hz, 1H, CH_2_-N-28), 4.21 (dd, *J* = 14.5, 4.4 Hz, 1H, CH_2_-N-28), 3.89 (d, *J* = 9.6 Hz, 1H, H-3), 3.41 (s, 3H, OCH_3_-2), 3.29 (td, *J* = 10.1, 9.7, 4.2 Hz, 1H, H-2), 2.13 (dd, *J* = 12.5, 4.3 Hz, 1H), 1.19 (s, 3H), 1.10 (s, 3H), 0.99 (s, 3H), 0.86 (d, *J* = 6.4 Hz, 3H), 0.69 (s, 3H). ^13^C-NMR (101 MHz, CDCl_3_) δ 181.18 (C-23), 178.00 (C-28), 139.92, 138.20, 128.67, 127.94, 127.44, 125.14 (C-12), 78.20 (C-2), 78.03 (C-3), 56.78 (OCH_3_-2), 53.89 (C-4), 53.15, 50.80, 47.72, 47.57, 43.79, 42.53, 42.22, 39.69, 39.56, 39.05, 37.96, 37.18, 32.25, 30.84, 27.79, 24.76, 23.40, 21.21, 20.35, 17.20, 17.13, 16.92, 12.10. HRMS (ESI) observed C_38_H_56_NO_5_ 606.4163 (MH^+^) requires 606.4153.

#### 3.1.9. α-Methoxy-3β-Acetoxy-Urs-12-Ene-28-Benzylamide-23-Oic Acid (**8**)

Compound **7** (300 mg, 0.5 mmol) was dissolved in pyridine (5 mL) and added acetic anhydride (0.5 mL), stirred at room temperature overnight. The reaction mixture was poured into dilute aqueous HCl (2 M), and extracted with DCM (100 mL). The organic phase was washed with dilute aqueous HCl (2 M, 200 mL), a saturated solution of sodium bicarbonate (200 mL × 2) and brine, followed by dried over anhydrous Na_2_SO_4_ and concentrated to dryness under vacuum. The crude product was chromatographed over silica gel (200–300 mesh) using hexane/AcOEt (2:1) to afford **8** as a white solid (304 mg, 94%). IR (CH_2_Cl_2_, cm^−1^) ν_max_: 3420, 2923 (C=C-H), 1739 (C=O), 1635 (C=O), 1525 (C=C). ^1^H-NMR (400 MHz, CDCl_3_) δ 6.17 (t, *J* = 5.2 Hz, 1H, NH-28), 5.29 (d, *J* = 9.9 Hz, 1H, H-3), 5.22 (d, *J* = 3.5 Hz, 1H, H-12), 4.53 (dd, *J* = 14.4, 5.6 Hz, 1H, CH_2_-N-28), 4.21 (dd, *J* = 14.5, 4.3 Hz, 1H, CH_2_-N-28), 3.39 (td, *J* = 10.9, 4.2 Hz, 1H, H-2), 3.34 (s, 3H, OCH_3_-2), 2.12 (dd, *J* = 12.7, 4.3 Hz, 1H), 2.06 (s, 3H, CH_3_COO-3), 1.21 (s, 3H), 1.10 (s, 3H), 1.00 (s, 3H), 0.86 (d, *J* = 6.3 Hz, 3H), 0.68 (s, 3H). ^13^C-NMR (101 MHz, CDCl_3_) δ 179.78 (C-23), 177.96 (C-28), 170.33 (COO-3), 139.98, 138.23, 128.67, 127.95, 127.43, 125.06 (C-12), 78.55 (C-3), 75.88 (C-2), 57.32 (OCH_3_-2), 53.92, 52.74, 50.34, 47.73, 47.61, 43.78, 43.51, 42.52, 39.67, 39.55, 39.05, 37.36, 37.17, 32.18, 30.84, 27.77, 24.75, 23.31, 21.20, 20.90, 20.56, 17.23, 17.03, 16.90, 12.75. HRMS (ESI) observed C_40_H_58_NO_6_ 648.4257 (MH^+^) requires 648.4259.

#### 3.1.10. α-Methoxy-3β-Hydroxy-Urs-12-Ene-28-Benzylamide-23-Methyl Ester (**9**)

Under a nitrogen atmosphere, compound **7** (600 mg, 1 mmol) was dissolved in dry DMF (2 mL), and K_2_CO_3_ (207 mg, 1.5 mmol) was added, and stirred for 20 min. Then CH_3_I (96 μL, 1.5 mmol) was added, and stirring was continued for 30 min at room temperature. The reaction mixture was neutralized with dilute aqueous HCl (1 M, 100 mL) and extracted with ethyl acetate (100 mL × 3). The combined organic phase was washed with a saturated solution of sodium bicarbonate and brine (200 mL × 3), followed by dried over anhydrous Na_2_SO_4_ and concentrated to dryness under vacuum.

The crude product was chromatographed over silica gel (200–300 mesh) using hexane/AcOEt (5:2) to afford compound **9** as a white solid (551 mg, 89%). IR (CH_2_Cl_2_, cm^−1^) ν_max_: 3420, 2921 (C=C-H), 1725 (C=O), 1639 (C=O), 1518 (C=C). ^1^H-NMR (400 MHz, CDCl_3_) δ 6.14 (t, *J* = 5.3 Hz, 1H, NH-28), 5.24 (t, *J* = 3.5 Hz, 1H, H-12), 4.54 (dd, *J* = 14.5, 5.9 Hz, 1H, CH_2_-N-28), 4.20 (dd, *J* = 14.5, 4.5 Hz, 1H, CH_2_-N-28), 3.86 (d, *J* = 9.6 Hz, 1H, H-3), 3.74 (s, 3H, OCH_3_-23), 3.40 (s, 3H, OCH_3_-2), 3.28 (ddd, *J* = 11.2, 9.6, 4.3 Hz, 1H, H-2), 2.13 (dd, *J* = 12.5, 4.3 Hz, 1H), 1.19 (s, 3H), 1.11 (s, 3H), 0.99 (s, 3H), 0.86 (d, *J* = 6.4 Hz, 3H), 0.69 (s, 3H). ^13^C-NMR (101 MHz, CDCl_3_) δ 177.75 (C-28), 177.24 (C-23), 139.95, 138.33, 128.67, 127.92, 127.41, 125.10 (C-12), 78.23 (C-2), 78.20 (C-3), 56.70 (OCH_3_-2), 53.91, 53.33, 52.30, 51.27, 47.71, 47.57, 43.71, 42.54, 42.07, 39.71, 39.58, 39.07, 38.01, 37.24, 32.27, 30.85, 27.80, 24.81, 23.38, 21.20, 20.43, 17.18, 16.89, 12.19. HRMS (ESI) observed C_39_H_58_NO_5_ 620.4308 (MH^+^) requires 620.4310.

#### 3.1.11. α-Methoxy-3β-Acetoxy-Urs-12-Ene-28-Benzylamide-23-Methyl Ester (**10**)

Following the procedure given for **8**, compound **10** (298 mg, 90%) was obtained from compound **9** as a white solid (silica gel, hexane/AcOEt, 3:1). IR (CH_2_Cl_2_, cm^−1^) ν_max_: 3412, 2919 (C=C-H), 1735 (C=O), 1639 (C=O), 1517 (C=C). ^1^H-NMR (400 MHz, CDCl_3_) δ 6.13 (d, *J* = 5.2 Hz, 1H, NH-28), 5.24 (d, *J* = 3.6 Hz, 1H, H-3), 5.20 (d, *J* = 9.9 Hz, 1H, H-12), 4.54 (dd, *J* = 14.5, 5.5 Hz, 1H, CH_2_-N-28), 4.20 (dd, *J* = 14.4, 4.1 Hz, 1H, CH_2_-N-28), 3.67 (s, 3H, OCH_3_-23), 3.39 (ddt, *J* = 12.2, 6.8, 3.3 Hz, 1H, H-2), 3.34 (s, 3H, OCH_3_-2), 2.13 (dd, *J* = 12.7, 4.5 Hz, 1H), 2.05 (s, 3H, CH_3_COO-3), 1.22 (s, 3H), 1.12 (s, 3H), 1.00 (s, 3H), 0.69 (s, 3H). ^13^C-NMR (101 MHz, CDCl_3_) δ 177.74 (C-28), 175.78 (C-23), 169.96 (COO-3), 139.93, 138.34, 128.66, 127.92, 127.40, 125.06 (C-12), 78.75 (C-3), 75.86 (C-2), 57.42 (OCH_3_-2), 53.92, 52.97, 52.53, 50.33, 47.72, 47.59, 43.71, 43.52, 42.52, 39.70, 39.58, 39.07, 37.31, 37.26, 32.23, 30.85, 27.80, 26.91, 24.79, 23.32, 21.19, 20.95, 20.71, 17.20, 17.04, 16.87, 13.01. HRMS (ESI) observed C_41_H_60_NO_6_ 662.4398 (MH^+^) requires 662.4415.

#### 3.1.12. α-Methoxy-3-Oxo-Urs-12-Ene-28-Benzyl Amide-23-Methyl Ester (**11**)

Compound **9** (62 mg, 0.1 mmol) was dissolved in DCM (5 mL), added PCC (65 mg, 0.3 mmol) and stirred at room temperature overnight. The reaction mixture was added water (100 mL) and extracted with DCM. The organic phase was washed with brine, dried over anhydrous Na_2_SO_4_ and concentrated to dryness under vacuum. The crude product was chromatographed over silica gel (200–300 mesh) using hexane/AcOEt (5:1) to afford compound **10** as a white solid (28 mg, 46%). IR (CH_2_Cl_2_, cm^−1^) ν_max_: 3418, 2920 (C=C-H), 1741 (C=O), 1719 (C=O), 1640 (C=O), 1521 (C=C). ^1^H-NMR (400 MHz, CDCl_3_) δ 6.14 (t, *J* = 5.3 Hz, 1H, NH-28), 5.24 (t, *J* = 3.5 Hz, 1H, H-12), 4.54 (dd, *J* = 14.5, 5.8 Hz, 1H, CH_2_-N-28), 4.21 (dd, *J* = 14.6, 4.6 Hz, 1H, CH_2_-N-28), 3.99 (dd, *J* = 11.3, 6.3 Hz, 1H, H-2), 3.75 (s, 3H, OCH_3_-23), 3.44 (s, 3H, OCH_3_-2), 2.29 (td, *J* = 12.9, 12.1, 4.3 Hz, 2H), 1.42 (s, 3H), 1.18 (s, 3H), 1.12 (s, 3H), 0.73 (s, 3H). ^13^C-NMR (101 MHz, CDCl_3_) δ 208.25 (C-3), 177.67 (C-28), 172.83 (C-23), 140.02, 138.34, 128.67, 127.91, 127.42, 124.86 (C-12), 78.64 (C-2), 62.21 (OCH_3_-2), 58.43 (23-OCH_3_), 53.93, 52.56, 51.35, 47.72, 47.31, 46.61, 43.68, 42.58, 39.69, 39.55, 39.05, 37.26, 36.83, 31.94, 30.83, 27.84, 26.91, 24.75, 23.38, 21.18, 21.00, 17.19, 16.98, 16.76, 16.63. HRMS (ESI) observed C_39_H_56_NO_5_ 618.4131 (MH^+^) requires 618.4153.

#### 3.1.13. α-Methoxy-3β-Acetoxy-23-Oxo-Urs-12-Ene-28-Benzyl Amide (**12**)

Following the procedure given for **8**, compound **12** (290 mg, 91%) was obtained from compound **6** as a white solid (silica gel, hexane/AcOEt, 4:1). IR (CH_2_Cl_2_, cm^−1^) ν_max_: 3376, 2923 (C=C-H), 1734 (C=O), 1640 (C=O), 1522 (C=C). ^1^H-NMR (400 MHz, CDCl_3_) δ 9.28 (s, 1H, H-23), 6.12 (t, *J* = 5.3 Hz, 1H, NH-28), 5.25 (t, *J* = 3.5 Hz, 1H, H-12), 5.02 (d, *J* = 9.8 Hz, 1H, H-3), 4.52 (dd, *J* = 14.5, 5.7 Hz, 1H, CH_2_-N-28), 4.22 (dd, *J* = 14.5, 4.6 Hz, 1H, CH_2_-N-28), 3.57–3.41 (m, 1H, H-2), 3.37 (s, 3H, OCH_3_-2), 2.19 (dd, *J* = 12.8, 4.6 Hz, 1H), 2.03 (s, 3H, CH_3_COO-3), 1.12 (s, 3H), 1.08 (s, 3H), 1.03 (s, 3H), 0.70 (s, 3H). ^13^C-NMR (101 MHz, CDCl_3_) δ 202.69 (C-23), 177.61 (C-28), 170.31 (COO-3), 139.94, 138.36, 128.66, 127.91, 127.40, 124.95 (C-12), 75.43 (C-3), 75.25 (C-2), 57.65 (OCH_3_-2), 55.48 (C-4), 53.90, 47.70, 47.50, 47.45, 43.67, 42.56, 39.68, 39.59, 39.05, 37.43, 37.27, 32.05, 30.83, 27.76, 26.91, 24.76, 23.31, 21.19, 20.87, 19.95, 17.21, 16.99, 16.95, 10.35. HRMS (ESI) observed C_40_H_58_NO_5_ 632.4316 (MH^+^) requires 632.4310.

#### 3.1.14. α-Methoxy-3β-Hydroxy-Urs-12-Ene-28-Benzyl Amide-23-Amide (**13a**)

To a solution of compound **7** (60 mg, 0.1 mmol) in dry DMF (1 mL), HOBt (25 mg, 0.2 mmol), EDCI·HCl (20 mg, 0.12 mmol), DIPEA (20 μL) and NH_4_HCO_3_ (15.8 mg, 0.2 mmol) was added, stirring at 85 °C overnight. The reaction mixture was poured into the brine, and extracted with ethyl acetate (100 mL × 3). The organic phase was washed with a saturated solution of sodium bicarbonate and brine (200 mL × 3), dried over Na_2_SO_4_ and concentrated to dryness under vacuum. The crude product was purified by column chromatography over silica gel (200–300 mesh) using hexane/AcOEt (1:4) to afford compound **13a** as a white solid (51 mg, 84%). IR (CH_2_Cl_2_, cm^−1^) ν_max_: 3361, 2919 (C=C-H), 1643 (C=O), 1522 (C=C). ^1^H-NMR (400 MHz, CDCl_3_) δ 6.16 (t, *J* = 5.4 Hz, 1H, NH-28), 6.14 (s, 1H, NH-23), 5.87 (s, 1H, NH-23), 5.23 (t, *J* = 3.4 Hz, 1H, H-12), 4.54 (dd, *J* = 14.5, 5.8 Hz, 1H, CH_2_-N-28), 4.19 (dd, *J* = 14.6, 4.4 Hz, 1H, CH_2_-N-28), 3.82 (d, *J* = 9.5 Hz, 1H, H-3), 3.40 (s, 3H, OCH_3_-2), 3.26 (tt, *J* = 11.3, 5.6 Hz, 1H, H-2), 2.70 (br s, 1H, OH-3), 2.09 (dd, *J* = 12.4, 4.1 Hz, 1H), 1.23 (s, 3H), 1.10 (s, 3H), 1.00 (s, 3H), 0.85 (d, *J* = 6.4 Hz, 3H), 0.68 (s, 3H). ^13^C-NMR (101 MHz, CDCl_3_) δ 179.63 (C-28), 177.80 (C-23), 140.02, 138.36, 128.65, 127.89, 127.39, 125.05 (C-12), 78.34 (C-2), 77.70 (C-3), 56.75 (OCH_3_-2), 53.92, 52.57, 50.39, 47.71, 47.56, 43.69, 42.57, 41.90, 39.68, 39.59, 39.07, 37.79, 37.25, 32.30, 30.84, 27.78, 24.81, 23.46, 23.38, 21.19, 20.17, 17.28, 17.21, 16.95, 12.50. HRMS (ESI) observed C_38_H_57_N_2_O_4_ 605.4309 (MH^+^) requires 605.4313.

#### 3.1.15. α-Methoxy-3β-Hydroxy-Urs-12-Ene-28-Benzyl Amide-23-Methylamide (**13b**)

As described for **13a**, compound **13b** (43 mg, 70%) was obtained from methylamine hydrochloride as a white solid (silica gel, hexane/AcOEt, 1:3). IR (CH_2_Cl_2_, cm^−1^) ν_max_: 3377, 2919 (C=C-H), 1636 (C=O), 1534 (C=C). ^1^H-NMR (400 MHz, CDCl_3_) δ 6.15 (t, *J* = 5.2 Hz, 1H, NH-28), 6.05 (q, *J* = 4.8 Hz, 1H, NH-23), 5.23 (t, *J* = 3.5 Hz, 1H, H-12), 4.55 (dd, *J* = 14.5, 5.9 Hz, 1H, CH_2_-N-28), 4.19 (dd, *J* = 14.5, 4.4 Hz, 1H, CH_2_-N-28), 3.86 (d, *J* = 9.7 Hz, 1H, H-3), 3.39 (s, 3H, OCH_3_-2), 3.34–3.18 (m, 1H, H-2), 2.85 (d, *J* = 4.5 Hz, 3H, CH_3_-N-23), 1.20 (s, 3H), 1.11 (s, 3H), 1.00 (s, 3H), 0.86 (d, *J* = 6.4 Hz, 3H), 0.69 (s, 3H). ^13^C-NMR (101 MHz, CDCl_3_) δ 177.83 (C-28), 177.04 (C-23), 140.06, 138.36, 128.66, 127.90, 127.39, 125.07 (C-12), 78.40 (C-2), 77.59 (C-3), 56.55 (OCH_3_-2), 53.95, 52.36, 50.40, 47.73, 47.55, 43.70, 42.60, 41.69, 39.68, 39.60, 39.08, 37.77, 37.25, 32.34, 30.85, 27.80, 26.87, 24.83, 23.43, 23.39, 21.19, 20.17, 17.29, 17.20, 16.97, 12.19. HRMS (ESI) observed C_39_H_59_N_2_O_4_ 619.4471 (MH^+^) requires 619.4469.

#### 3.1.16. α-Methoxy-3β-Hydroxy-Urs-12-Ene-28-Benzyl Amide-23-Ethylamide (**13c**)

As described for **13a**, compound **13c** (38 mg, 60%) was obtained from ethylamine as a white solid (silica gel, hexane/AcOEt, 1:1). IR (CH_2_Cl_2_, cm^−1^) ν_max_: 3372, 2922 (C=C-H), 1636 (C=O), 1528 (C=C). ^1^H-NMR (400 MHz, CDCl_3_) δ 6.15 (t, *J* = 5.2 Hz, 1H, NH-28), 5.97 (t, *J* = 5.5 Hz, 1H, NH-23), 5.23 (t, *J* = 3.4 Hz, 1H, H-12), 4.55 (dd, *J* = 14.5, 5.9 Hz, 1H, CH_2_-N-28), 4.19 (dd, *J* = 14.6, 4.4 Hz, 1H, CH_2_-N-28), 3.85 (d, *J* = 9.7 Hz, 1H, H-3), 3.39 (s, 3H, OCH_3_-2), 3.37–3.21 (m, 1H, H-2), 2.09 (dd, *J* = 12.4, 4.3 Hz, 1H), 1.11 (s, 3H), 0.99 (s, 3H), 0.85 (d, *J* = 6.4 Hz, 3H), 0.68 (s, 3H). ^13^C-NMR (101 MHz, CDCl_3_) δ 177.85 (C-28), 176.16 (C-23), 140.05, 138.35, 128.65, 127.90, 127.39, 125.09 (C-12), 78.45 (C-2), 77.53 (C-3), 56.58 (OCH_3_-2), 53.94, 52.15, 50.39, 47.73, 47.55, 43.70, 42.59, 41.74, 39.68, 39.59, 39.08, 37.77, 37.24, 34.88, 32.33, 30.85, 27.79, 24.83, 23.42, 23.39, 21.19, 20.07, 17.29, 17.20, 16.96, 14.80, 12.21. HRMS (ESI) observed C_40_H_61_N_2_O_4_ 633.4599 (MH^+^) requires 633.4626.

#### 3.1.17. α-Methoxy-3β-Hydroxy-Urs-12-Ene-28-Benzyl Amide-23-Isopropylamide (**13d**)

As described for **13a**, compound **13d** (48 mg, 75%) was obtained from isopropylamine as a white solid (silica gel, hexane/AcOEt, 3:2). IR (CH_2_Cl_2_, cm^−1^) ν_max_: 3372, 2923 (C=C-H), 1637 (C=O), 1522 (C=C). ^1^H-NMR (400 MHz, CDCl_3_) δ 6.16 (t, *J* = 5.3 Hz, 1H, NH-28), 5.73 (d, *J* = 7.7 Hz, 1H, NH-23), 5.22 (t, *J* = 3.5 Hz, 1H, H-12), 4.54 (dd, *J* = 14.6, 5.9 Hz, 1H, CH_2_-N-28), 4.18 (dd, *J* = 14.6, 4.4 Hz, 1H, CH_2_-N-28), 3.84 (d, *J* = 9.7 Hz, 1H, H-3), 3.38 (s, 3H, OCH_3_-2), 3.34–3.20 (m, 1H, H-2), 2.42 (br s, 1H, OH-3), 2.08 (dd, *J* = 12.4, 4.3 Hz, 1H), 1.10 (s, 3H), 0.99 (s, 3H), 0.85 (d, *J* = 6.4 Hz, 3H), 0.68 (s, 3H). ^13^C-NMR (101 MHz, CDCl_3_) δ 177.84 (C-28), 175.33 (C-23), 140.04, 138.36, 128.64, 127.89, 127.37, 125.09 (C-12), 78.47 (C-2), 77.49 (C-3), 56.58 (OCH_3_-2), 53.93, 51.96, 50.31, 47.72, 47.54, 43.69, 42.59, 41.77, 41.73, 39.68, 39.59, 39.07, 37.76, 37.24, 32.31, 30.85, 27.79, 24.82, 23.43, 23.39, 22.83, 22.59, 21.19, 20.02, 17.28, 17.20, 16.96, 12.23. HRMS (ESI) observed C_41_H_63_N_2_O_4_ 647.4764 (MH^+^) requires 647.4782.

#### 3.1.18. α-Methoxy-3β-Hydroxy-Urs-12-Ene-28-Benzyl Amide-23-Propargylamide (**13e**)

As described for **13a**, compound **13e** (50 mg, 78%) was obtained from propargylamine as a white solid (silica gel, hexane/AcOEt, 1:1). IR (CH_2_Cl_2_, cm^−1^) ν_max_: 3372, 2923 (C=C-H), 1640 (C=O), 1520 (C=C). ^1^H-NMR (400 MHz, CDCl_3_) δ 6.28 (t, *J* = 5.2 Hz, 1H, NH-23), 6.15 (t, *J* = 5.3 Hz, 1H, NH-28), 5.22 (t, *J* = 3.4 Hz, 1H, H-12), 4.54 (dd, *J* = 14.5, 5.9 Hz, 1H, CH_2_-N-28), 4.19 (dd, *J* = 14.6, 4.4 Hz, 1H, CH_2_-N-28), 4.16–4.10 (m, 1H), 3.99 (ddd, *J* = 17.5, 4.6, 2.6 Hz, 1H), 3.81 (d, *J* = 9.7 Hz, 1H, H-3), 3.38 (s, 3H, OCH_3_-2), 3.27 (td, *J* = 10.1, 9.5, 4.1 Hz, 1H, H-2), 2.23 (t, *J* = 2.5 Hz, 1H), 1.21 (s, 3H), 1.10 (s, 3H), 0.99 (s, 3H), 0.85 (d, *J* = 6.4 Hz, 3H), 0.68 (s, 3H). ^13^C-NMR (101 MHz, CDCl_3_) δ 177.80 (C-28), 176.39 (C-23), 140.03, 138.35, 128.65, 127.89, 127.38, 125.04 (C-12), 79.80 (alkyne), 78.36 (C-2), 77.48 (C-3), 71.51 (terminal alkyne), 56.63 (OCH_3_-2), 53.92, 52.31, 50.58, 47.71, 47.55, 43.69, 42.57, 41.73, 39.67, 39.58, 39.06, 37.80, 37.25, 32.27, 30.84, 29.75, 27.78, 24.81, 23.41, 23.38, 21.19, 20.11, 17.29, 17.20, 16.96, 12.19. HRMS (ESI) observed C_41_H_59_N_2_O_4_ 643.4473 (MH^+^) requires 643.4469. 

#### 3.1.19. *N*-(2α-Methoxy-3β-Hydroxy-Urs-12-Ene-28-Benzylamide-23-Oyl)-l-Alanine Methyl Ester (**13f**)

As described for **13a**, compound **13f** (45 mg, 65%) was obtained from l-alanine methyl ester as a white solid (silica gel, hexane/AcOEt, 1:1). IR (CH_2_Cl_2_, cm^−1^) ν_max_: 3437, 2918 (C=C-H), 1738 (C=O), 1637 (C=O), 1523 (C=C). ^1^H-NMR (400 MHz, CDCl_3_) δ 6.55 (d, *J* = 7.0 Hz, 1H, NH-23), 6.15 (m, 1H, NH-28), 5.23 (t, *J* = 3.4 Hz, 1H, H-12), 4.58 (d, *J* = 7.0 Hz, 1H), 4.55 (dd, *J* = 14.5, 5.9 Hz, 1H, CH_2_-N-28), 4.19 (dd, *J* = 14.5, 4.4 Hz, 1H, CH_2_-N-28), 3.77 (d, *J* = 9.7 Hz, 1H, H-3), 3.76 (s, 3H, CH_3_-ester), 3.41 (s, 3H, OCH_3_-2), 3.29 (ddd, *J* = 11.2, 9.6, 4.3 Hz, 1H, H-2), 2.16–2.05 (m, 1H), 1.43 (d, *J* = 7.2 Hz, 3H), 1.25 (s, 3H), 1.10 (s, 3H), 1.01 (s, 3H), 0.85 (d, *J* = 6.4 Hz, 3H), 0.69 (s, 3H). ^13^C-NMR (101 MHz, CDCl_3_) δ 177.82 (C-28), 176.42 (C-23), 173.97 (amino acid ester), 140.04, 138.36, 128.65, 127.90, 127.38, 125.09 (C-12), 78.02 (C-2), 77.85 (C-3), 56.84 (OCH_3_-2), 53.93, 52.52, 52.47, 50.01, 48.73, 47.72, 47.61, 43.69, 42.58, 42.09, 39.68, 39.60, 39.07, 37.64, 37.24, 32.29, 30.85, 27.79, 24.82, 23.42, 23.37, 21.20, 20.28, 18.06, 17.32, 17.21, 16.98, 12.09. HRMS (ESI) observed C_42_H_63_N_2_O_6_ 691.4665 (MH^+^) requires 691.4681.

#### 3.1.20. *N*-(2α-Methoxy-3β-Hydroxy-Urs-12-Ene-28-Benzylamide-23-Oyl)-l-Valine Methyl Ester (**13g**)

As described for **13a**, compound **13g** (40 mg, 56%) was obtained from l-valine methyl ester as a white solid (silica gel, hexane/AcOEt, 1:1). IR (CH_2_Cl_2_, cm^−1^) ν_max_: 3438, 2919 (C=C-H), 1738 (C=O), 1643 (C=O), 1517 (C=C). ^1^H-NMR (400 MHz, CDCl_3_) δ 6.52 (d, *J* = 8.1 Hz, 1H, NH-23), 6.16 (t, *J* = 5.2 Hz, 1H, NH-28), 5.23 (t, *J* = 3.5 Hz, 1H, H-12), 4.62–4.43 (m, 1H, CH_2_-N-28), 4.19 (dd, *J* = 14.6, 4.4 Hz, 1H, CH_2_-N-28), 3.77 (d, *J* = 9.7 Hz, 1H, H-3), 3.75 (s, 3H, CH_3_-ester), 3.41 (s, 3H, OCH_3_-2), 3.28 (ddd, *J* = 11.3, 9.6, 4.2 Hz, 1H, H-2), 2.20 (pd, *J* = 6.9, 4.6 Hz, 1H), 1.11 (s, 3H), 1.02 (s, 3H), 0.85 (d, *J* = 6.4 Hz, 3H), 0.69 (s, 3H). ^13^C-NMR (101 MHz, CDCl_3_) δ 177.84 (C-28), 176.88 (C-23), 172.87 (amino acid ester), 140.07, 138.36, 128.65, 127.90, 127.38, 125.09 (C-12), 78.04 (C-2), 77.94 (C-3), 57.83, 56.88 (OCH_3_-2), 53.94, 52.67, 52.21, 49.78, 47.73, 47.66, 43.69, 42.60, 42.04, 39.67, 39.65, 39.07, 37.61, 37.24, 32.31, 30.84, 27.78, 24.83, 23.41, 23.38, 21.19, 20.52, 19.06, 18.07, 17.35, 17.21, 17.03, 12.21. HRMS (ESI) observed C_44_H_67_N_2_O_6_ 719.4993 (MH^+^) requires 719.4994.

#### 3.1.21. *N*-(2α-Methoxy-3β-Hydroxy-Urs-12-Ene-28-Benzylamide-23-oyl)-l-Phenylalanine Methyl Ester (**13h**)

As described for **13a**, compound **13h** (37 mg, 48%) was obtained from l-phenylalanine methyl ester as a white solid (silica gel, hexane/AcOEt, 2:1). IR (CH_2_Cl_2_, cm^−1^) ν_max_: 3396, 2919 (C=C-H), 1740 (C=O), 1638 (C=O), 1517 (C=C). ^1^H-NMR (400 MHz, CDCl_3_) δ 7.20–7.06 (m, 2H), 6.41 (d, *J* = 7.5 Hz, 1H, NH-23), 6.15 (t, *J* = 5.2 Hz, 1H, NH-28), 5.22 (t, *J* = 3.2 Hz, 1H, H-12), 4.87 (q, *J* = 6.7 Hz, 1H), 4.55 (dd, *J* = 14.6, 5.5 Hz, 1H, CH_2_-N-28), 4.19 (m, 1H, CH_2_-N-28), 3.74 (s, 3H, CH_3_-ester), 3.70 (d, *J* = 9.6 Hz, 1H, H-3), 3.39 (s, 3H, OCH_3_-2), 3.30–3.20 (m, 1H, H-2), 3.20–3.05 (m, 2H), 1.14 (s, 3H), 1.09 (s, 3H), 0.66 (s, 3H). ^13^C-NMR (101 MHz, CDCl_3_) δ 177.86 (C-28), 176.55 (C-23), 172.51(amino acid ester), 140.05, 138.34, 129.11, 128.66, 128.62, 127.91, 127.40, 127.14, 125.09 (C-12), 78.00 (C-2), 77.85 (C-3), 56.81 (OCH_3_-2), 53.94, 53.63, 52.55, 52.43, 49.88, 47.74, 47.60, 43.72, 42.55, 41.97, 39.68, 39.59, 39.08, 37.85, 37.59, 37.25, 32.32, 30.85, 27.81, 24.82, 23.44, 23.36, 21.20, 20.13, 17.28, 17.22, 17.00, 12.01. HRMS (ESI) observed C_48_H_67_N_2_O_6_ 767.4978 (MH^+^) requires 767.4994. 

#### 3.1.22. *N*-(2α-Methoxy-3β-Hydroxy-Urs-12-Ene-28-Benzylamide-23-Oyl)-4-Aminobutyric Ethyl Ester (**13i**)

As described for **13a**, compound **13i** (32 mg, 45%) was obtained from 4-aminobutyric ethyl ester as a white solid (silica gel, hexane/AcOEt, 1:2). IR (CH_2_Cl_2_, cm^−1^) ν_max_: 3361, 2924 (C=C-H), 1732 (C=O), 1634 (C=O), 1526 (C=C). ^1^H-NMR (400 MHz, CDCl_3_) δ 6.33 ((t, *J* = 5.5 Hz, 1H, NH-23), 6.15 (t, *J* = 5.3 Hz, 1H, NH-28), 5.22 (d, *J* = 3.6 Hz, 1H, H-12), 4.53 (dd, *J* = 14.5, 5.7 Hz, 1H, CH_2_-N-28), 4.25–4.04 (m, 3H), 3.80 (d, *J* = 9.5 Hz, 1H, H-3), 3.38 (s, 3H, OCH_3_-2), 2.64 (br s, 1H, OH-3), 2.43–2.29 (m, 2H), 2.08 (dd, *J* = 12.5, 4.1 Hz, 1H), 1.18 (s, 3H), 0.84 (d, *J* = 6.4 Hz, 3H), 0.67 (s, 3H). ^13^C-NMR (101 MHz, CDCl_3_) δ 177.98 (C-28), 176.67 (C-23), 173.65 (amino acid ester), 139.96, 138.22, 128.65, 127.88, 127.40, 125.13 (C-12), 78.42 (C-2), 77.55 (C-3), 60.56, 56.63 (OCH_3_-2), 53.90, 52.37, 50.33, 47.71, 47.54, 43.73, 42.55, 41.80, 39.66, 39.57, 39.04, 37.73, 37.19, 32.29, 31.82, 30.82, 27.74, 24.78, 24.32, 23.37, 21.18, 20.15, 17.29, 17.18, 16.92, 14.23, 12.12. HRMS (ESI) observed C_44_H_67_N_2_O_6_ 719.4978 (MH^+^) requires 719.4994.

#### 3.1.23. α-Methoxy-3β-Hydroxy-Urs-12-Ene-28-Benzyl Amide-23-Dimethylamide (**13j**)

As described for **13a**, compound **13j** (51 mg, 80%) was obtained from dimethyamine hydrochloride as a white solid (silica gel, hexane/AcOEt, 2:3). IR (CH_2_Cl_2_, cm^−1^) ν_max_: 3372, 2921 (C=C-H), 1630 (C=O), 1522 (C=C). ^1^H-NMR (400 MHz, CDCl_3_) δ 6.16 (t, *J* = 5.3 Hz, 1H, NH-28), 5.22 (t, *J* = 3.4 Hz, 1H, H-12), 4.53 (dd, *J* = 14.5, 5.9 Hz, 1H, CH_2_-N-28), 4.18 (dd, *J* = 14.5, 4.5 Hz, 1H, CH_2_-N-28), 4.10 (d, *J* = 9.5 Hz, 1H, H-3), 3.39 (s, 3H, OCH_3_-2), 3.28 (ddd, *J* = 11.2, 9.4, 4.2 Hz, 1H, H-2), 3.07 (s, 6H, dimethyl-23), 2.09 (dd, *J* = 12.6, 4.5 Hz, 1H), 1.33 (s, 3H), 1.08 (s, 3H), 0.84 (d, *J* = 6.4 Hz, 3H), 0.68 (s, 3H). ^13^C-NMR (101 MHz, CDCl_3_) δ 177.82 (C-28), 177.49 (C-23), 139.97, 138.36, 128.65, 127.90, 127.38, 125.15 (C-12), 79.22 (C-2), 78.42 (C-3), 56.62 (OCH_3_-2), 54.27, 53.93, 51.76, 47.73, 47.56, 43.69, 42.61, 42.09, 39.70, 39.67, 39.41, 39.07, 38.08, 37.24, 32.29, 30.85, 27.79, 24.82, 23.41, 23.33, 21.19, 20.79, 17.20, 17.11, 16.98, 15.99. HRMS (ESI) observed C_40_H_61_N_2_O_4_ 633.4620 (MH^+^) requires 633.4626.

#### 3.1.24. α-Methoxy-3β-Hydroxy-Urs-12-Ene-28-Benzylamide-23- (4-Methyl-1-Piperazinyl)-Amide (**13k**)

As described for **13a**, compound **13k** (48 mg, 70%) was obtained from methylpiperazine as a white solid (silica gel, DCM/MeOH, 30:1). IR (CH_2_Cl_2_, cm^−1^) ν_max_: 3393, 2922 (C=C-H), 1630 (C=O), 1523 (C=C). ^1^H-NMR (400 MHz, CDCl_3_) δ 6.13 (t, *J* = 5.2 Hz, 1H, NH-28), 5.24 (t, *J* = 3.5 Hz, 1H, H-12), 4.55 (dd, *J* = 14.6, 5.9 Hz, 1H, CH_2_-N-28), 4.20 (dd, *J* = 14.6, 4.5 Hz, 1H, CH_2_-N-28), 3.96 (d, *J* = 9.5 Hz, 1H, H-3), 3.72 (s, 4H), 3.40 (s, 3H, OCH_3_-2), 3.27 (ddd, *J* = 11.1, 9.4, 4.2 Hz, 1H, H-2), 2.44 (q, *J* = 4.9 Hz, 4H), 2.32 (s, 3H), 2.10 (dd, *J* = 12.4, 4.2 Hz, 1H), 1.12 (s, 3H), 1.02 (s, 3H), 0.86 (d, *J* = 6.4 Hz, 3H), 0.71 (s, 3H). ^13^C-NMR (126 MHz, CDCl_3_) δ 177.79 (C-28), 177.79 (C-23), 140.04, 138.40, 128.68, 127.92, 127.41, 125.14 (C-12), 78.52 (C-2), 78.52 (C-3), 56.66 (OCH_3_-2), 55.35, 54.17, 53.95, 51.34, 47.83, 47.74, 46.18, 45.92, 45.83, 43.70, 42.67, 41.91, 39.69, 39.51, 39.08, 38.03, 37.27, 32.37, 30.86, 27.80, 24.85, 23.41, 23.35, 21.20, 20.88, 17.21, 17.12, 15.44, 8.77. HRMS (ESI) observed C_43_H_66_N_3_O_4_ 688.5028 (MH^+^) requires 688.5048.

#### 3.1.25. α-Methoxy-3β-Acetoxy-Urs-12-Ene-28-Benzyl Amide-23-Amide (**14a**)

As described for **13a**, compound **14a** (52 mg, 80%) was obtained from **8** (65 mg, 0.1 mmol) and NH_4_HCO_3_ as a white solid (silica gel, hexane/AcOEt, 2:3). IR (CH_2_Cl_2_, cm^−1^) ν_max_: 3451, 2917 (C=C-H), 1735 (C=O), 1654 (C=O), 1537 (C=C). ^1^H-NMR (400 MHz, CDCl_3_) δ 6.15 (t, *J* = 5.3 Hz, 1H, NH-28), 6.15 (br s, 1H, NH-23), 5.85 (br s, 1H, NH-23), 5.28 (d, *J* = 9.9 Hz, 1H, H-3), 5.23 (t, *J* = 3.6 Hz, 1H, H-12), 4.53 (dd, *J* = 14.5, 5.8 Hz, 1H, CH_2_-N-28), 4.20 (dd, *J* = 14.5, 4.4 Hz, 1H, CH_2_-N-28), 3.39 (td, *J* = 10.7, 4.3 Hz, 1H, H-2), 3.32 (s, 3H, OCH_3_-2), 2.11 (dd, *J* = 12.7, 4.4 Hz, 1H), 2.05 (s, 3H, CH_3_COO-3), 1.09 (s, 3H), 1.02 (s, 3H), 0.86 (d, *J* = 6.4 Hz, 3H), 0.68 (s, 3H). ^13^C-NMR (101 MHz, CDCl_3_) δ 177.73 (C-28), 177.72 (C-23), 169.93 (COO-3), 139.98, 138.36, 128.65, 127.88, 127.39, 124.98 (C-12), 78.54 (C-3), 76.19 (C-2), 57.07 (OCH_3_-2), 53.88, 52.43, 51.23, 47.70, 47.58, 43.68, 43.18, 42.51, 39.67, 39.54, 39.05, 37.60, 37.26, 32.26, 30.82, 27.77, 24.78, 23.40, 23.36, 21.20, 21.03, 19.97, 17.29, 17.24, 16.87, 13.22. HRMS (ESI) observed C_40_H_59_N_2_O_5_ 647.4413 (MH^+^) requires 647.4418.

#### 3.1.26. α-Methoxy-3β-Acetoxy-Urs-12-Ene-28-Benzyl Amide-23-Methylamide (**14b**)

As described for **13a**, compound **14b** (49 mg, 74%) was obtained from **8** (65 mg, 0.1 mmol) and methylamine hydrochloride as a white solid (silica gel, hexane/AcOEt, 2:3). IR (CH_2_Cl_2_, cm^−1^) ν_max_: 3404, 2920 (C=C-H), 1735 (C=O), 1640 (C=O), 1529 (C=C). ^1^H-NMR (400 MHz, CDCl_3_) δ 6.15 (t, *J* = 5.3 Hz, 1H, NH-28), 6.05 (q, *J* = 4.7 Hz, 1H, NH-23), 5.28–5.19 (m, 2H, H-3 and H-12), 4.53 (dd, *J* = 14.5, 5.9 Hz, 1H, CH_2_-N-28), 4.19 (dd, *J* = 14.5, 4.5 Hz, 1H, CH_2_-N-28), 3.39 (td, *J* = 10.7, 4.3 Hz, 1H, H-2), 3.31 (s, 3H, OCH_3_-2), 2.78 (d, *J* = 4.5 Hz, 3H, CH_3_-N-23), 2.10 (dd, *J* = 12.7, 4.4 Hz, 1H), 2.03 (s, 3H, CH_3_COO-3), 1.24 (s, 3H), 1.09 (s, 3H), 1.01 (s, 3H), 0.86 (d, *J* = 6.4 Hz, 3H), 0.67 (s, 3H). ^13^C-NMR (101 MHz, CDCl_3_) δ 177.77 (C-28), 175.26 (C-23), 169.76 (COO-3), 139.99, 138.34, 128.66, 127.88, 127.40, 125.01 (C-12), 78.63 (C-3), 76.22 (C-2), 57.03 (OCH_3_-2), 53.91, 52.40, 51.12, 47.71, 47.57, 43.68, 43.13, 42.53, 39.67, 39.55, 39.06, 37.50, 37.27, 32.30, 30.83, 27.78, 26.98, 24.79, 23.39, 23.35, 21.19, 21.01, 20.07, 17.31, 17.22, 16.87, 13.02. HRMS (ESI) observed C_41_H_61_N_2_O_5_ 661.4560 (MH^+^) requires 661.4575.

#### 3.1.27. α-Methoxy-3β-Acetoxy-Urs-12-Ene-28-Benzyl Amide-23-Ethylamide (**14c**)

As described for **13a**, compound **14c** (47 mg, 70%) was obtained from **8** (65 mg, 0.1 mmol) and ethylamine as a white solid (silica gel, hexane/AcOEt, 3:2). IR (CH_2_Cl_2_, cm^−1^) ν_max_: 3372, 2924 (C=C-H), 1740 (C=O), 1639 (C=O), 1526 (C=C). ^1^H-NMR (400 MHz, CDCl_3_) δ 6.14 (t, *J* = 5.3 Hz, 1H, NH-28), 5.88 (t, *J* = 5.5 Hz, 1H, NH-23), 5.32–5.13 (m, 2H, H-3 and H-12), 4.54 (dd, *J* = 14.5, 5.9 Hz, 1H, CH_2_-N-28), 4.19 (dd, *J* = 14.6, 4.5 Hz, 1H, CH_2_-N-28), 3.39 (td, *J* = 10.8, 10.4, 4.2 Hz, 1H, H-2), 3.32 (s, 3H, OCH_3_-2), 3.26–3.15 (m, 1H), 2.11 (dd, *J* = 12.7, 4.4 Hz, 1H), 2.04 (s, 3H, CH_3_COO-3), 1.23 (s, 3H), 1.02 (s, 3H), 0.86 (d, *J* = 6.4 Hz, 3H), 0.68 (s, 3H). ^13^C-NMR (101 MHz, CDCl_3_) δ 177.78 (C-28), 174.30 (C-23), 169.66 (COO-3), 140.01, 138.36, 128.65, 127.89, 127.38, 125.00 (C-12), 78.53 (C-3), 76.19 (C-2), 57.03 (OCH_3_-2), 53.91, 52.26, 51.04, 47.71, 47.64, 43.68, 43.20, 42.53, 39.68, 39.56, 39.07, 37.46, 37.26, 34.86, 32.32, 30.84, 27.78, 24.80, 23.36, 21.19, 20.96, 20.03, 17.33, 17.23, 16.89, 14.82, 12.98. HRMS (ESI) observed C_41_H_61_N_2_O_5_ 675.4712 (MH^+^) requires 675.4731.

#### 3.1.28. α-Methoxy-3β-Acetoxy-Urs-12-Ene-28-Benzyl Amide-23-Isopropylamide (**14d**)

As described for **13a**, compound **14d** (58 mg, 84%) was obtained from **8** (65 mg, 0.1 mmol) and isopropylamine as a white solid (silica gel, hexane/AcOEt, 2:1). IR (CH_2_Cl_2_, cm^−1^) ν_max_: 3403, 2921 (C=C-H), 1740 (C=O), 1637 (C=O), 1526 (C=C).^1^ H-NMR (400 MHz, CDCl_3_) δ 6.15 (t, *J* = 5.3 Hz, 1H, NH-28), 5.61 (d, *J* = 7.7 Hz, 1H, NH-23), 5.25–5.19 (m, 2H, H-3 and H-12), 4.54 (dd, *J* = 14.5, 5.9 Hz, 1H, CH_2_-N-28), 4.19 (dd, *J* = 14.6, 4.5 Hz, 1H, CH_2_-N-28), 4.13–3.97 (m, 1H), 3.44–3.33 (m, 1H, H-2), 3.32 (s, 3H, OCH_3_-2), 2.11 (dd, *J* = 12.7, 4.4 Hz, 1H), 2.03 (s, 3H, CH_3_COO-3), 1.21 (s, 3H), 1.01 (s, 3H), 0.86 (d, *J* = 6.4 Hz, 3H), 0.67 (s, 3H). ^13^C NMR (101 MHz, CDCl_3_) δ 177.78 (C-28), 173.42 (C-23), 169.56 (COO-3), 140.01, 138.37, 128.64, 127.88, 127.37, 125.01 (C-12), 78.46 (C-3), 76.17 (C-2), 57.02 (OCH_3_-2), 53.91, 52.13, 51.00, 47.71, 47.67, 43.67, 43.22, 42.52, 41.63, 39.67, 39.56, 39.06, 37.43, 37.25, 32.34, 30.84, 27.77, 24.79, 23.34, 22.73, 22.55, 21.19, 20.92, 20.02, 17.33, 17.22, 16.89, 12.92. HRMS (ESI) observed C_42_H_65_N_2_O_5_ 689.4893 (MH^+^) requires 689.4888.

#### 3.1.29. α-Methoxy-3β-Acetoxy-Urs-12-Ene-28-Benzyl Amide-23-Propargylamide (**14e**)

As described for **13a**, compound **14e** (54 mg, 79%) was obtained from **8** (65 mg, 0.1 mmol) and propargylamine as a white solid (silica gel, hexane/AcOEt, 3:1). IR (CH_2_Cl_2_, cm^−1^) ν_max_: 3379, 2922 (C=C-H), 1737 (C=O), 1643 (C=O), 1521 (C=C). ^1^H-NMR (400 MHz, CDCl_3_) δ 6.16 (t, *J* = 5.3 Hz, 1H, NH-28), 6.15 (t, *J* = 5.2 Hz, 1H, NH-23), 5.34–5.16 (m, 2H, H-3 and H-12), 4.54 (dd, *J* = 14.5, 5.8 Hz, 1H, CH_2_-N-28), 4.19 (dd, *J* = 14.5, 4.4 Hz, 1H, CH_2_-N-28), 4.08 (ddd, *J* = 17.5, 5.5, 2.6 Hz, 1H), 3.95 (ddd, *J* = 17.5, 4.6, 2.5 Hz, 1H), 3.39 (td, *J* = 10.8, 4.4 Hz, 1H, H-2), 3.32 (s, 3H, OCH_3_-2), 2.22 (t, *J* = 2.5 Hz, 1H), 2.11 (dd, *J* = 12.7, 4.4 Hz, 1H), 2.04 (s, 3H, CH_3_COO-3), 1.10 (s, 3H), 1.02 (s, 3H), 0.86 (d, *J* = 6.4 Hz, 3H), 0.68 (s, 3H). ^13^C-NMR (101 MHz, CDCl_3_) δ 177.77 (C-28), 174.49 (C-23), 169.72 (COO-3), 140.01, 138.35, 128.66, 127.89, 127.40, 124.97 (C-12), 79.70 (alkyne), 78.39 (C-3), 76.10 (C-2), 71.46 (terminal alkyne), 57.08 (OCH_3_-2), 53.92, 52.47, 51.04, 47.72, 47.61, 43.69, 43.15, 42.54, 39.68, 39.55, 39.07, 37.49, 37.26, 32.24, 30.84, 29.77, 27.79, 24.79, 23.37, 21.20, 21.00, 20.07, 17.34, 17.22, 16.89, 12.89. HRMS (ESI) observed C_43_H_61_N_2_O_5_ 685.4553 (MH^+^) requires 685.4575.

#### 3.1.30. *N*-(2α-Methoxy-3β-Acetoxy-Urs-12-Ene-28-Benzylamide-23-oyl)-l-Alanine Methyl Ester (**14f**) 

As described for **13a**, compound **14f** (62 mg, 84%) was obtained from **8** (65 mg, 0.1 mmol) and l-alanine methyl ester as a white solid (silica gel, hexane/AcOEt, 5:2). IR (CH_2_Cl_2_, cm^−1^) ν_max_: 3391, 2921 (C=C-H), 1742 (C=O), 1640 (C=O), 1525 (C=C). ^1^H-NMR (400 MHz, CDCl_3_) δ 6.39 (d, *J* = 7.2 Hz, 1H, NH-23), 6.14 (t, *J* = 5.4 Hz, 1H, NH-28), 5.23 (t, *J* = 3.9 Hz, 1H, H-12), 5.19 (d, *J* = 9.9 Hz, 1H, H-3), 4.56 (ddd, *J* = 14.4, 11.3, 6.6 Hz, 2H), 4.19 (dd, *J* = 14.6, 4.4 Hz, 1H, CH_2_-N-28), 3.74 (s, 3H, CH_3_-ester), 3.39 (td, *J* = 10.8, 4.3 Hz, 1H, H-2), 3.33 (s, 3H, OCH_3_-2), 2.04 (s, 3H, CH_3_COO-3), 1.03 (s, 3H), 0.86 (d, *J* = 6.4 Hz, 3H), 0.68 (d, *J* = 2.3 Hz, 3H). ^13^C-NMR (101 MHz, CDCl_3_) δ 177.80 (C-28), 174.11 (C-23), 173.62 (amino acid ester), 169.85 (COO-3), 140.04, 138.36, 128.65, 127.90, 127.39, 124.98 (C-12), 78.30 (C-3), 76.12 (C-2), 57.13 (OCH_3_-2), 53.93, 52.40, 52.23, 50.95, 48.30, 47.72, 43.69, 43.24, 42.55, 39.68, 39.57, 39.07, 37.43, 37.25, 32.24, 30.84, 27.77, 24.80, 23.35, 21.19, 20.96, 20.10, 18.35, 17.30, 17.23, 16.92, 12.84. HRMS (ESI) observed C_44_H_65_N_2_O_7_ 733.4770 (MH^+^) requires 733.4786.

#### 3.1.31. *N*-(2α-Methoxy-3β-Acetoxy-Urs-12-Ene-28-Benzylamide-23-Oyl)-l-Valine Methyl Ester (**14g**)

As described for **13a**, compound **14g** (57 mg, 75%) was obtained from **8** (65 mg, 0.1 mmol) and l-valine methyl ester as a white solid (silica gel, hexane/AcOEt, 4:1). IR (CH_2_Cl_2_, cm^−1^) ν_max_: 3420, 2959, 2921 (C=C-H), 1741 (C=O), 1638 (C=O), 1515 (C=C). ^1^H-NMR (400 MHz, CDCl_3_) δ 6.28 (d, *J* = 8.3 Hz, 1H, NH-23), 6.15 (t, *J* = 5.2 Hz, 1H, NH-28), 5.24 (t, *J* = 3.4 Hz, 1H, H-12), 5.17 (d, *J* = 9.9 Hz, 1H, H-3), 4.62–4.44 (m, 2H), 4.19 (dd, *J* = 14.6, 4.4 Hz, 1H, CH_2_-N-28), 3.73 (s, 3H, CH_3_-ester), 3.39 (td, *J* = 10.8, 4.3 Hz, 1H, H-2), 3.33 (s, 3H, OCH_3_-2), 2.04 (s, 3H, CH_3_COO-3), 1.12 (s, 3H), 1.03 (s, 3H), 0.86 (d, *J* = 6.4 Hz, 3H), 0.68 (s, 3H). ^13^C-NMR (101 MHz, CDCl_3_) δ 177.84 (C-28), 174.63 (C-23), 172.74 (amino acid ester), 169.76 (COO-3), 140.07, 138.35, 128.65, 127.90, 127.39, 124.99 (C-12), 78.26 (C-3), 76.19 (C-2), 57.41, 57.09 (OCH_3_-2), 53.95, 52.52, 52.11, 50.66, 47.73, 47.71, 43.69, 43.12, 42.57, 39.68, 39.58, 39.07, 37.35, 37.24, 32.19, 31.14, 30.84, 27.76, 24.81, 23.34, 21.19, 20.96, 20.29, 18.98, 17.83, 17.31, 17.23, 16.94, 12.80. HRMS (ESI) observed C_46_H_69_N_2_O_7_ 761.5092 (MH^+^) requires 761.5099.

#### 3.1.32. *N*-(2α-Methoxy-3β-Acetoxy-Urs-12-Ene-28-Benzylamide-23-Oyl)-l-Phenylalanine Methyl Ester (**14h**)

As described for **13a**, compound **14h** (47 mg, 58%) was obtained from **8** (65 mg, 0.1 mmol) and l-phenylalanine methyl ester as a white solid (silica gel, hexane/AcOEt, 4:1). IR (CH_2_Cl_2_, cm^−1^) ν_max_: 3373, 2953, 2921 (C=C-H), 1742 (C=O), 1640 (C=O), 1525 (C=C). ^1^H-NMR (400 MHz, CDCl_3_) δ 7.17–7.07 (m, 3H, ), 6.16 (d, *J* = 7.5 Hz, 1H, NH-23), 6.15 (t, *J* = 5.2 Hz, 1H, NH-28), 5.23 (t, *J* = 3.5 Hz, 1H, H-12), 5.14 (d, *J* = 10.0 Hz, 1H, H-3), 4.90 (dt, *J* = 7.9, 6.1 Hz, 1H), 4.55 (dd, *J* = 14.5, 5.9 Hz, 1H, CH_2_-N-28 ), 4.20 (dd, *J* = 14.5, 4.4 Hz, 1H, CH_2_-N-28), 3.72 (s, 3H, CH_3_-ester), 3.36 (dt, *J* = 10.8, 5.4 Hz, 1H, H-2), 3.31 (s, 3H, OCH_3_-2), 3.20–3.00 (m, 3H), 2.08 (dd, *J* = 12.7, 4.4 Hz, 1H), 2.00 (s, 3H, CH_3_COO-3), 1.18 (s, 3H), 0.99 (s, 3H), 0.67 (s, 3H). ^13^C-NMR (101 MHz, CDCl_3_) δ 177.81 (C-28), 174.22 (C-23), 172.17 (amino acid ester), 169.74 (COO-3), 140.06, 138.37, 135.94, 129.18, 128.65, 128.57, 127.90, 127.39, 127.11, 124.97 (C-12), 78.23 (C-3), 76.00 (C-2), 57.07 (OCH_3_-2), 53.94, 52.97, 52.29, 52.26, 50.28, 47.74, 47.63, 43.69, 43.08, 42.53, 39.68, 39.55, 39.09, 38.03, 37.26, 32.24, 31.51, 30.85, 30.14, 27.80, 24.81, 23.39, 23.32, 21.20, 20.89, 20.03, 17.24, 16.94, 12.60. HRMS (ESI) observed C_50_H_69_N_2_O_7_ 809.5068 (MH^+^) requires 809.5099.

#### 3.1.33. *N*-(2α-Methoxy-3β-Acetoxy-Urs-12-Ene-28-Benzylamide-23-Oyl)-4-Aminobutyric Ethyl Ester (**14i**)

As described for **13a**, compound **14i** (49 mg, 64%) was obtained from **8** (65 mg, 0.1 mmol) and 4-aminobutyric ethyl ester as a white solid (silica gel, hexane/AcOEt, 3:2). IR (CH_2_Cl_2_, cm^−1^) ν_max_: 3372, 2924 (C=C-H), 1737 (C=O), 1639 (C=O), 1525 (C=C). ^1^H-NMR (400 MHz, CDCl_3_) δ 6.26 (t, *J* = 5.5 Hz, 1H, NH-23), 6.14 (t, *J* = 5.4 Hz, 1H, NH-28), 5.30–5.15 (m, 2H, H-3 and H-12), 4.54 (dd, *J* = 14.5, 5.8 Hz, 1H, CH_2_-N-28), 4.29–3.98 (m, 3H, CH_2_-N-28 and CH_2_-ester), 3.38 (m, 1H, H-2), 3.32 (s, 3H, OCH_3_-2), 3.19 (dq, *J* = 13.0, 6.6 Hz, 1H), 2.34 (t, *J* = 7.0 Hz, 2H), 2.11 (dd, *J* = 12.7, 4.4 Hz, 1H), 1.10 (s, 3H), 1.01 (s, 3H), 0.86 (d, *J* = 6.4 Hz, 3H), 0.68 (s, 3H). ^13^C-NMR (101 MHz, CDCl_3_) δ 177.76 (C-28), 174.70 (C-23), 173.66 (amino acid ester), 169.67 (COO-3), 140.01, 138.36, 128.65, 127.89, 127.38, 125.01 (C-12), 78.57 (C-3), 76.19 (C-2), 60.59, 57.05 (OCH_3_-2), 53.92, 52.37, 51.02, 47.71, 47.63, 43.68, 43.20, 42.53, 39.67, 39.56, 39.06, 37.46, 37.25, 32.30, 31.86, 30.83, 27.78, 24.79, 24.27, 23.34, 21.18, 20.95, 20.09, 17.29, 17.22, 16.89, 14.22, 12.89. HRMS (ESI) observed C_46_H_69_N_2_O_7_ 761.5096 (MH^+^) requires 761.5099.

#### 3.1.34. α-Methoxy-3β-Acetoxy-Urs-12-Ene-28-Benzyl Amide-23-Dimethylamide (**14j**)

As described for **13a**, compound **14j** (42 mg, 62%) was obtained from **8** (65 mg, 0.1 mmol) and dimethyamine hydrochloride as a white solid (silica gel, hexane/AcOEt, 1:1). IR (CH_2_Cl_2_, cm^−1^) ν_max_: 3411, 2920 (C=C-H), 1740 (C=O), 1632 (C=O), 1522 (C=C). ^1^H-NMR (400 MHz, CDCl_3_) δ 6.12 (t, *J* = 5.2 Hz, 1H, NH-28), 5.58 (d, *J* = 9.3 Hz, 1H, H-3), 5.24 (q, *J* = 6.0, 4.7 Hz, 1H, H-12), 4.53 (dd, *J* = 14.6, 5.7 Hz, 1H, CH_2_-N-28), 4.21 (dd, *J* = 14.5, 4.6 Hz, 1H, CH_2_-N-28), 3.36 (s, 3H, OCH_3_-2), 3.16 (s, 4H), 2.05 (s, 3H, CH_3_COO-3), 1.11 (s, 3H), 1.04 (s, 3H), 0.87 (d, *J* = 6.4 Hz, 3H), 0.70 (s, 3H). ^13^C-NMR (101 MHz, MeOD) δ 178.37 (C-28), 174.31 (C-23), 170.73 (COO-3), 138.94, 138.64, 128.03, 127.57, 126.68, 125.18 (H-12), 76.87 (C-3), 76.54 (C-2), 65.24, 56.67 (OCH_3_-2), 53.67, 52.88, 49.25, 47.88, 42.99, 41.95, 39.42, 39.15, 38.89, 37.36, 32.45, 30.57, 30.37, 29.47, 27.55, 23.82, 23.10, 22.80, 20.30, 19.53, 18.91, 16.42, 16.15, 15.04, 12.79. HRMS (ESI) observed C_42_H_63_N_2_O_5_ 670.4709 (MH^+^) requires 675.4731.

#### 3.1.35. α-Methoxy-3β-Acetoxy-Urs-12-Ene-28-Benzylamide-23-(4-Methyl-1-Piperazinyl)-Amide (**14k**)

As described for **13a**, compound **14k** (33 mg, 45%) was obtained from **8** (65 mg, 0.1 mmol) and methylpiperazine as a white solid (silica gel, hexane/AcOEt, 1:3). IR (CH_2_Cl_2_, cm^−1^) ν_max_: 3383, 2925 (C=C-H), 1740 (C=O), 1635 (C=O), 1522 (C=C). ^1^H-NMR (400 MHz, CDCl_3_) δ 6.13 (t, *J* = 5.4 Hz, 1H, NH-28), 5.51 (d, *J* = 9.3 Hz, 1H, H-3), 5.23 (d, *J* = 3.7 Hz, 1H, H-12), 4.50 (ddd, *J* = 14.7, 6.0, 2.5 Hz, 1H, CH_2_-N-28), 4.20 (dd, *J* = 14.5, 4.5 Hz, 1H, CH_2_-N-28), 3.89 (s, 1H), 3.67 (s, 2H), 3.41 (d, *J* = 10.2 Hz, 1H, H-2), 3.34 (s, 3H, OCH_3_-2), 2.55–2.33 (m, 4H), 2.28 (d, *J* = 1.8 Hz, 3H), 2.22–2.09 (m, 1H), 2.02 (s, 3H, CH_3_COO-3), 1.09 (s, 3H), 1.01 (s, 3H), 0.85 (d, *J* = 6.3 Hz, 3H), 0.68 (d, *J* = 2.2 Hz, 3H). ^13^C-NMR (101 MHz, CDCl_3_) δ 177.56 (C-28), 172.21 (C-23), 170.17 (COO-3), 139.82, 138.35, 128.63, 127.89, 127.38, 125.05 (C-12), 77.30, 60.36, 57.65 (OCH_3_-2), 54.94, 53.86, 53.57, 50.23, 48.53, 47.69, 46.25, 45.77, 43.66, 43.41, 42.54, 39.68, 39.01, 37.44, 37.21, 32.50, 30.83, 27.79, 24.75, 23.43, 23.32, 21.19, 21.02, 20.41, 17.18, 17.04, 16.95, 16.00, 14.20. HRMS (ESI) observed C_45_H_68_N_3_O_5_ 730.5144 (MH^+^) requires 730.5153.

### 3.2. Biology

#### 3.2.1. Materials

All cell lines were obtained from the China State Institute of Pharmaceutical Industry (SIPI) (Shanghai). Fetal Bovine Serum (FBS) and Dulbecco’s Modified Eagle Medium (DMEM) with penicillin and streptomycin were obtained from PAA Laboratories GmbH (Pashing, Austria). 3-(4,5-Dimethylthiazol-2-yl)-2,5-diphenyltetrazolium bromide (MTT) was obtained from Amresco. A Cell Counting Kit-8 (CCK-8) was obtained from Beyotime Biotechnology (Shanghai, China). DMSO was obtained from Merck (Darmstadt, Germany). Doxorubicin was obtained from Iffect Chemphar Co., Ltd (Nanjing, China).

#### 3.2.2. In-Vitro Cytotoxicity of Human Cancer Cell Lines

HCT116, SKOV-3, A549, A2780, HepG2, HL-60 and MCF-7 cells were incubated in DMEM with 10% heat-inactivated FBS in a humidified atmosphere of 5.0% CO_2_ at 37 °C. The in-vitro cytotoxicity of AA derivatives was determined using MTT assay. A 100-μL volume of culture medium at a concentration of 3 × 10^4^ cell/mL was added to 96-well plate wells, which were maintained in a humidified atmosphere of 5.0% CO_2_ at 37 °C. After 24 h, 10 μL solution of AA derivatives and doxorubicin dissolved in DMSO and phophate buffered saline (PBS) and then diluted with DMEM under a concentration gradient, was added in the wells for final concentrations of 100, 10, 1, 0.1, 0.01, 0.001 μM of compounds in HCT116 and SKOV-3 cells and 125, 25, 5, 1, 0.2, 0.04 μM in A549, A2780, HepG2, HL-60 and MCF-7 cells. After 72 h incubation in a humidified atmosphere of 5.0% CO_2_ at 37 °C, 20 μl MTT solution (5 mg/mL) was added into each well and the cells were incubated. After 4 h, 100 μL HCl aqueous solution (0.01 M) with 10% SDS and 5% isobutanol was added into each well to dissolve the precipitated formazan and the absorbance was read at 570 nm on Thermo Scientific Varioskan Flash plate reader. All IC_50_ values were averaged from three independent experiments.

#### 3.2.3. In-Vitro Cytotoxicity of Human Normal Cell Line

HUVEC cell lines were incubated in DMEM with 10% heat-inactivated FBS in a humidified atmosphere of 5.0% CO_2_ at 37 °C. The in-vitro cytotoxicity of AA derivatives was determined using CCK-8. A 100-μL volume of culture medium at a concentration of 3 × 10^4^ cell/mL was added to 96-well plate wells, and these were maintained in a humidified atmosphere of 5.0% CO_2_ at 37 °C. After 24 h, 10 μL solution of AA derivatives dissolved in DMSO and PBS and then diluted with DMEM under a concentration gradient was added to the wells for final concentrations of 125, 25, 5, 1, 0.2, 0.04 μM. After 72 h incubation in a humidified atmosphere of 5.0% CO_2_ at 37 °C, 10 μl CCK-8 solution was added into each well and the cells were incubated for another 1 h, and the absorbance was read at 450 nm. All cell viability levels were averaged from three independent experiments.

## 4. Conclusions

In this paper, we reported a highly selective oxidation method on the C-23 hydroxyl group in an AA derivative, and then, for the first time with AA derivatives, the C-23 carboxyl group was modified to synthesize a series of novel AA derivatives. The selective oxidation method provided a high-yield route for more modification in the C-3 hydroxyl, C-23 aldehyde and C-23 carboxyl groups in AA.

The 23-amide AA derivatives **10**, **14j** and **14k** exhibited good cytotoxic activities against tumor cell lines but were significantly less cytotoxic against normal human cell line, showing excellent selectivity.

We studied the intramolecular hydrogen bonds of the 23-amide AA derivatives via exhaustive NMR characterization, and combined with their cytotoxic activities against SKOV-3 and HCT116 cell lines, which revealed a distinctive SAR associated with the intramolecular hydrogen bonding of the amide moiety at C-23. We found that the 23-amide AA derivatives, which fixed the amide hydrogen atom in the AA skeleton through intramolecular hydrogen bonding, showed rather good cytotoxic activities. Intramolecular hydrogen bond has been actively applied in drug design for its effects on receptor binding and its improved molecular properties, such as membrane permeability, water solubility, and lipophilicity. When a donor and an acceptor are in proximity on the same molecule, an equilibrium may exist between closed conformations in which an intramolecular hydrogen bond is formed, creating a temporary ring system, and open conformations in which the polar groups are exposed to solvent. The closed forms, hiding polarity from the environment, should be more lipophilic and might display a higher membrane permeability, whereas the open forms should be more water-soluble [33]. Especially for the triterpenoids, with a rigid skeleton that often leads to poor water solubility, dynamic and proper water solubility and lipophilicity may be crucial for activity and the pharmacokinetic profile. In this paper, these active AA derivatives with intramolecular hydrogen bonds in the A ring, can function as ideal starting points for further modification to develop compounds with better activities and improved molecular properties.

## Figures and Tables

**Table 1:** Cytotoxic activities (IC50)^a^ of AA, AA derivatives and doxorubicin against human ovarian (SKOV-3) and colon (HCT-116) cancer cell lines under the treatment with six increasing concentrations of each compound for 72 h. Abbreviation: N.D., not determined.

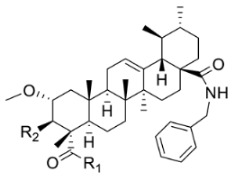				
			IC_50_ (μM)	
Compound	R_2_	R_1_	SKOV-3	HCT116
**5**	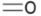	H	>100	25.99 ± 6.68
**6**	OH	H	N.D.	8.46 ± 1.64
**7**	OH	OH	31.91 ± 2.59	16.71 ± 1.68
**9**	OH	OCH_3_	6.81 ± 3.87	3.07 ± 1.48
**10**	OAc	OCH_3_	N.D.	1.08 ± 0.66
**11**	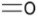	OCH_3_	>100	>100
**12**	OAc	H	>100	6.69 ± 1.15
**13a**	OH	NH_2_	N.D.	7.45 ± 1.62
**13b**	OH	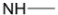	N.D.	7.45 ± 3.53
**13c**	OH	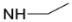	N.D.	10.95 ± 4.28
**13d**	OH	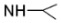	N.D.	8.57 ± 3.04
**13e**	OH	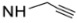	6.72 ± 4.32	8.03 ± 2.79
**13f**	OH	HNCH(CH_3_)COOCH_3_	3.09 ± 1.54	4.35 ± 1.05
**13g**	OH	(CH_3_)_2_CH(NH)COOCH_3_	N.D.	9.69 ± 1.64
**13h**	OH	PhCH_2_CH(HN)COOCH_3_	>100	>50
**13i**	OH	HN(CH_2_)_3_COOCH_2_CH_3_	N.D.	15.23 ± 1.99
**13j**	OH	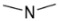	N.D.	9.6 ± 4.58
**14a**	OAc	NH_2_	7.12 ± 2.61	6.52 ± 3.03
**14b**	OAc	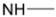	N.D.	34.24 ± 7.87
**14c**	OAc	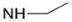	>100	>100
**14d**	OAc	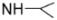	>100	>100
**14e**	OAc	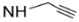	>100	>100
**14f**	OAc	HNCH(CH_3_)COOCH_3_	>100	>50
**14g**	OAc	(CH3)_2_CH(NH)COOCH_3_	>100	>100
**14h**	OAc	PhCH_2_CH(HN)COOCH_3_	>100	>100
**14i**	OAc	HN(CH_2_)_3_COOCH_2_CH_3_	>100	>100
**14j**	OAc	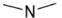	N.D.	1.83 ± 0.91
AA			20.51 ± 4.06	24.64 ± 3.26
Doxorubicin			0.08 ± 0.01	0.03 ± 0.02

(IC50)^a^ (μM) values are expressed as the mean ± SD of three independent experiments.

**Table 2:** ^13^C-NMR and ^1^H-NMR signals of the positions about intramolecular hydrogen bond in compounds **13** and **14** (CDCl_3_).

	C-2	C-3	C-23	NH-23 (4.85 mM)	
**13a**	78.34	77.70	177.80	5.94	5.44
**14a**	76.19	78.54	177.72	5.82	5.43
Δ_δ**13a**–δ**14a**_	2.15	−0.84	0.08	0.12	0.01
**13b**	78.40	77.59	177.04	5.96	
**14b**	76.22	78.63	175.26	5.84	
Δ_δ**13b**–δ**14b**_	2.18	−1.04	1.78	0.12	
**13c**	78.45	77.53	176.16	5.90	
**14c**	76.19	78.53	174.30	5.81	
Δ_δ**13c–**δ**14c**_	2.26	−1.00	1.86	0.09	
**13d**	78.47	77.49	175.33	5.71	
**14d**	76.17	78.46	173.42	5.60	
Δ_δ**13d–**δ**14d**_	2.30	−0.97	1.91	0.11	
**13e**	78.36	77.48	176.39	6.13	
**14e**	76.10	78.39	174.49	5.98	
Δ_δ**13e–**δ**14e**_	2.26	−0.91	1.90	0.15	
**13f**	78.02	77.85	176.42	6.53	
**14f**	76.12	78.30	174.11	6.39	
Δ_δ**13f–**δ**14f**_	1.90	−0.45	2.31	0.14	
**13g**	78.04	77.94	176.88	6.53	
**14g**	76.19	78.26	174.63	6.28	
Δ_δ**13g**–δ**14g**_	1.85	–0.32	2.25	0.25	
**13h**	78.00	77.85	176.55	6.40	
**14h**	76.00	78.23	174.22	6.17	
Δ_δ**13h**–δ**14h**_	2.00	–0.38	2.33	0.23	
**13i**	78.42	77.55	176.67	6.22	
**14i**	76.19	78.57	174.70	6.21	
Δ_δ**13i**–δ**14i**_	2.23	–1.02	1.97	0.01	

**Table 3:** Cytotoxic activities (IC50)^a^ of compounds **10**, **13k**, **14j** and **14k** against five human cancer cell lines and one human normal cell line under the treatment with six increasing concentrations of each compound for 72 h.

Compound	IC_50_ (μM)					
	A549	A2780	HepG2	HL-60	MCF-7	HUVEC
**10**	>125	1.28 ± 0.32	1.43 ± 0.32	0.47 ± 0.08	3.99 ± 1.21	>125
**14j**	6.72 ± 0.64	2.24 ± 0.52	3.77 ± 1.22	3.01 ± 0.44	5.89 ± 1.33	>125
**13k**	5.70 ± 0.58	12.97± 1.18	2.22 ± 0.68	10.73 ± 2.08	2.85 ± 0.25	N.D.
**14k**	4.04 ± 0.51	4.31 ± 0.58	2.84 ± 0.46	3.36 ±0.66	1.78 ± 0.38	>125
AA	41.02 ± 0.87 [17]	28.2 ± 0.3 [32]	35.37 ± 1.47 [17]	N.D.	68.5 ± 2.50 [21]	>100 [16]

(IC50)^a^ (μM) values are expressed as the mean ± SD of three independent experiments.

## Supplementary Materials

Supplementary Information shows the NMR spectra of compounds **1**-**14** and the NMR titration of **13b** and **14b**.
