# Supplementary material for: Synthesis and Cytotoxic Activity of Novel C-23-Modified Asiatic Acid Derivatives
Source: Molecules. 2020 Aug 14;25(16):3709. doi: 10.3390/molecules25163709 (PMC7464201; doi:10.3390/molecules25163709)
Supplement: Supplementary file 1 [file molecules-25-03709-s001.pdf]

Supplementary information

Synthesis and cytotoxic activity of novel C-23-modified asiatic acid derivatives

Contents	Page
^{1}H and ^{13}C NMR spectra of **1**	3
^{1}H and ^{13}C NMR spectra of **2**	4
^{1}H and ^{13}C NMR spectra of **3**	5
^{1}H and ^{13}C NMR spectra of **4**	6
^{1}H and ^{13}C NMR spectra of **5**	7
^{1}H and ^{13}C NMR spectra of **6**	8
^{1}H and ^{13}C NMR spectra of **7**	9
^{1}H and ^{13}C NMR spectra of **8**	10
^{1}H and ^{13}C NMR spectra of **9**	11
^{1}H and ^{13}C NMR spectra of **10**	12
^{1}H and ^{13}C NMR spectra of **11**	13
^{1}H and ^{13}C NMR spectra of **12**	14
^{1}H and ^{13}C NMR spectra of **13a**	15
^{1}H, ^{13}C, DEPT, HSQC and HMBC NMR spectra of **13b**	16-18
^{1}H and ^{13}C NMR spectra of **13c**	19
^{1}H and ^{13}C NMR spectra of **13d**	20
^{1}H and ^{13}C NMR spectra of **13e**	21
^{1}H and ^{13}C NMR spectra of **13f**	22
^{1}H and ^{13}C NMR spectra of **13g**	23
^{1}H and ^{13}C NMR spectra of **13h**	24
^{1}H and ^{13}C NMR spectra of **13i**	25
^{1}H and ^{13}C NMR spectra of **13j**	26
^{1}H and ^{13}C NMR spectra of **13k**	27
^{1}H and ^{13}C NMR spectra of **14a**	28
^{1}H, ^{13}C, DEPT, HSQC and HMBC NMR spectra of **14b**	29-31
^{1}H and ^{13}C NMR spectra of **14c**	32
^{1}H and ^{13}C NMR spectra of **14d**	33
^{1}H and ^{13}C NMR spectra of **14e**	34
^{1}H and ^{13}C NMR spectra of **14f**	35
^{1}H and ^{13}C NMR spectra of **14g**	36
^{1}H and ^{13}C NMR spectra of **14h**	37
^{1}H and ^{13}C NMR spectra of **14i**	38
^{1}H and ^{13}C NMR spectra of **14j**	39

^{1}H and ^{13}C NMR spectra of **14k**	40
NMR titration of **13b** and **14b**	41
IC_{50} curves of compounds **10**, **13k**, **14j** and **14k** against HL-60	42-43

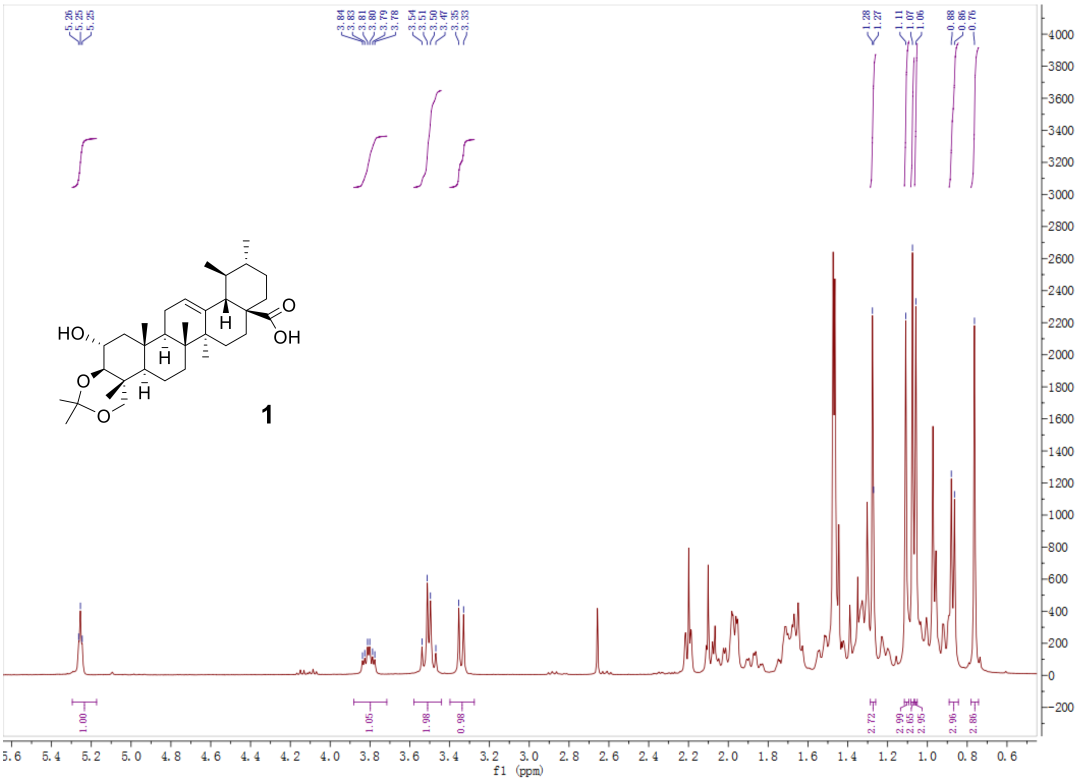

^{1}H NMR Spectrum of α-hydroxy-3β,23-isopropylidenedioxy-urs-12-ene-28-oic acid (**1**) (400 MHz, CDCl3)

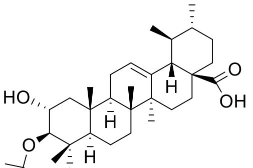

1

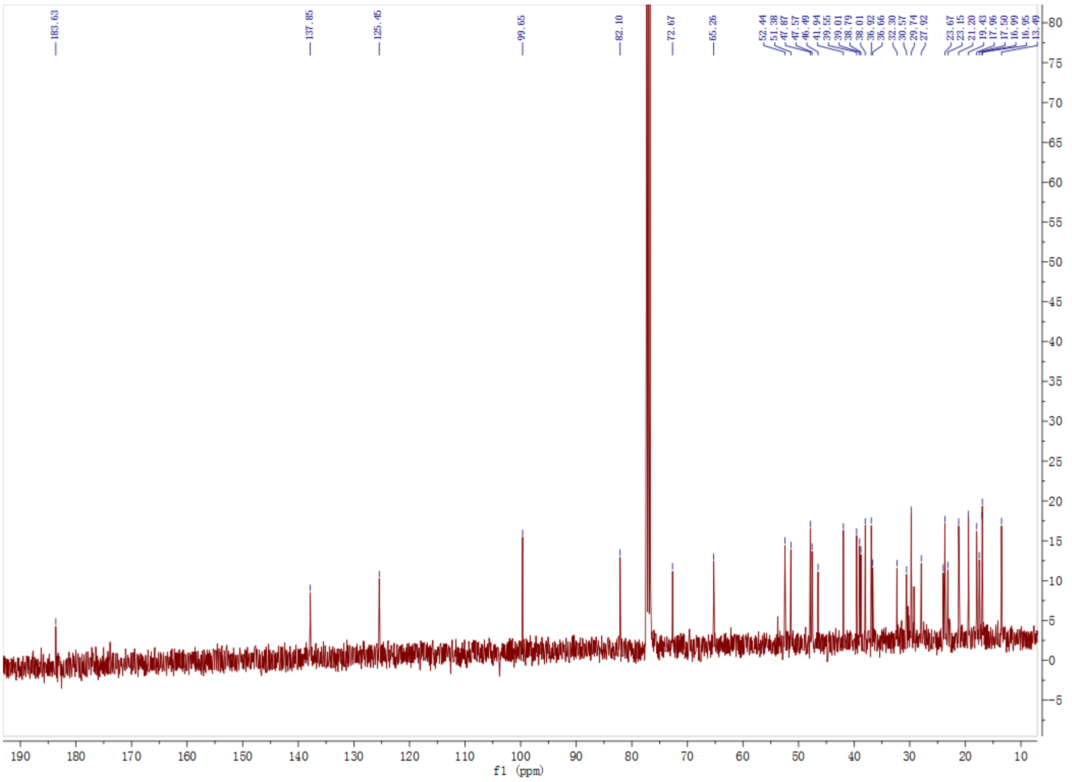

^{13}C NMR Spectrum of α-hydroxy-3β,23-isopropylidenedioxy-urs-12-ene-28-oic acid (**1**) (101 MHz, CDCl3)

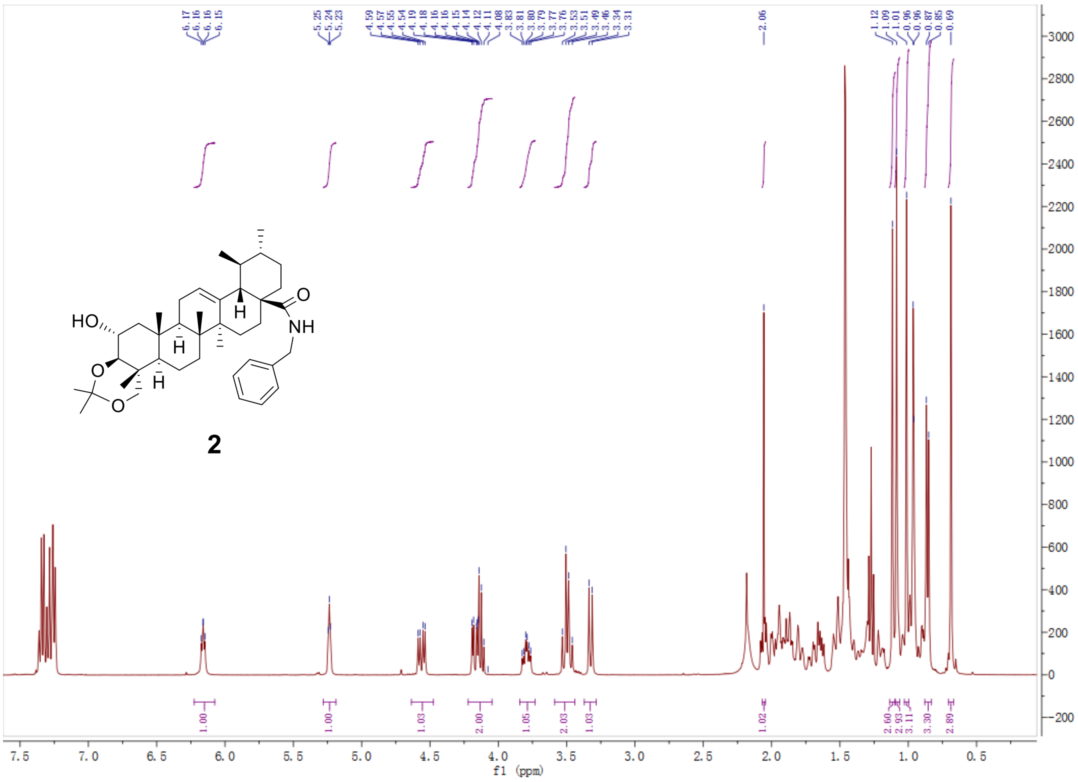

^{1}H NMR Spectrum of α-hydroxy-3β,23-isopropylidenedioxy-urs-12-ene-28-benzylamide (**2**) (400 MHz, CDCl3)

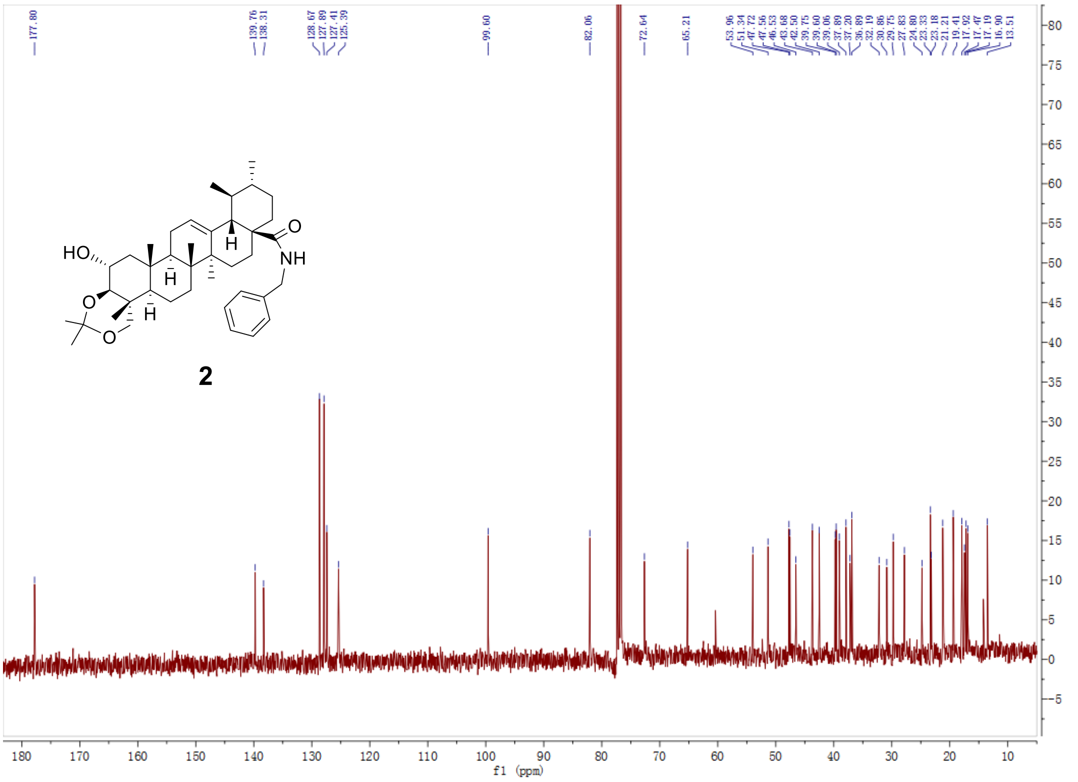

^{13}C NMR Spectrum of α-hydroxy-3β,23-isopropylidenedioxy-urs-12-ene-28-benzylamide (**2**) (101 MHz, CDCl3)

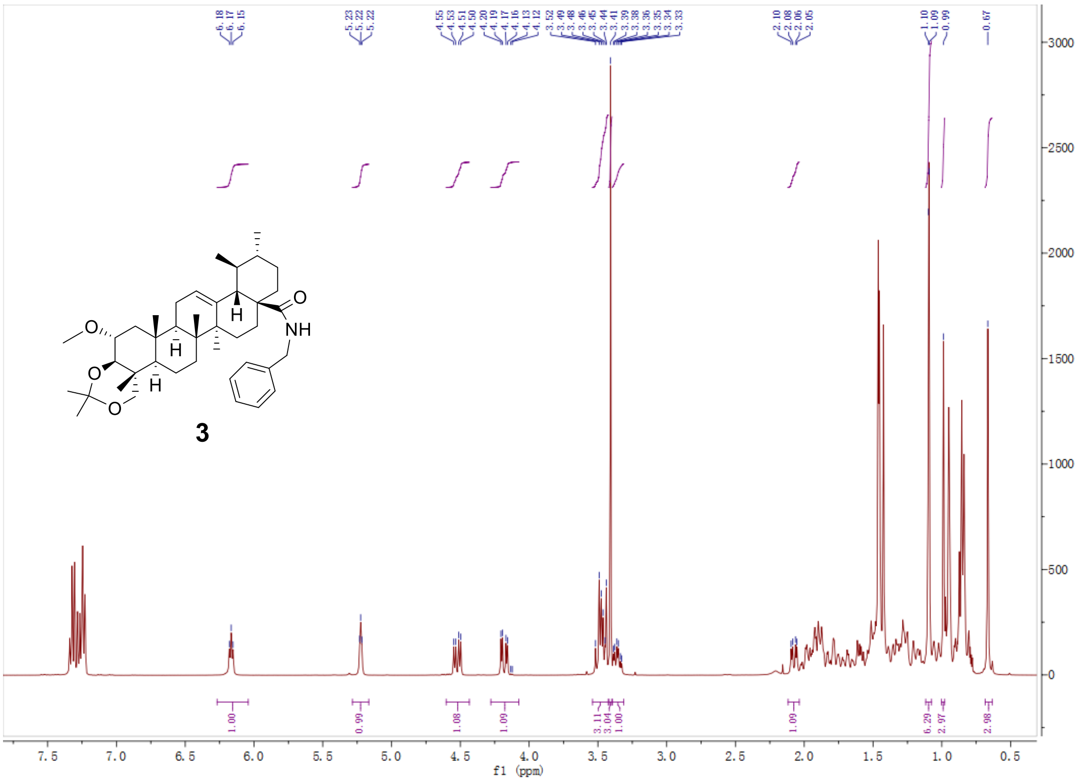

^{1}H NMR Spectrum of α-methoxy-3β,23-isopropylidenedioxy-urs-12-ene-28-benzylamide (**3**) (400 MHz, CDCl3)

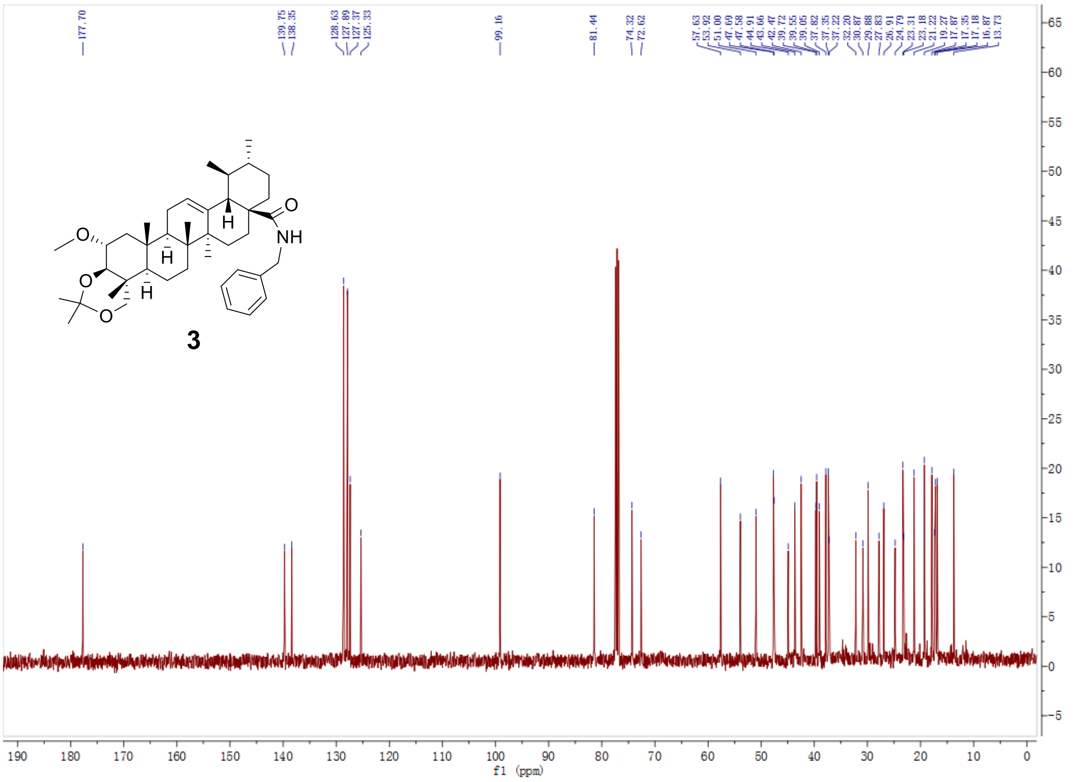

^{13}C NMR Spectrum of α-methoxy-3β,23-isopropylidenedioxy-urs-12-ene-28-benzylamide (**3**) (101 MHz, CDCl3)

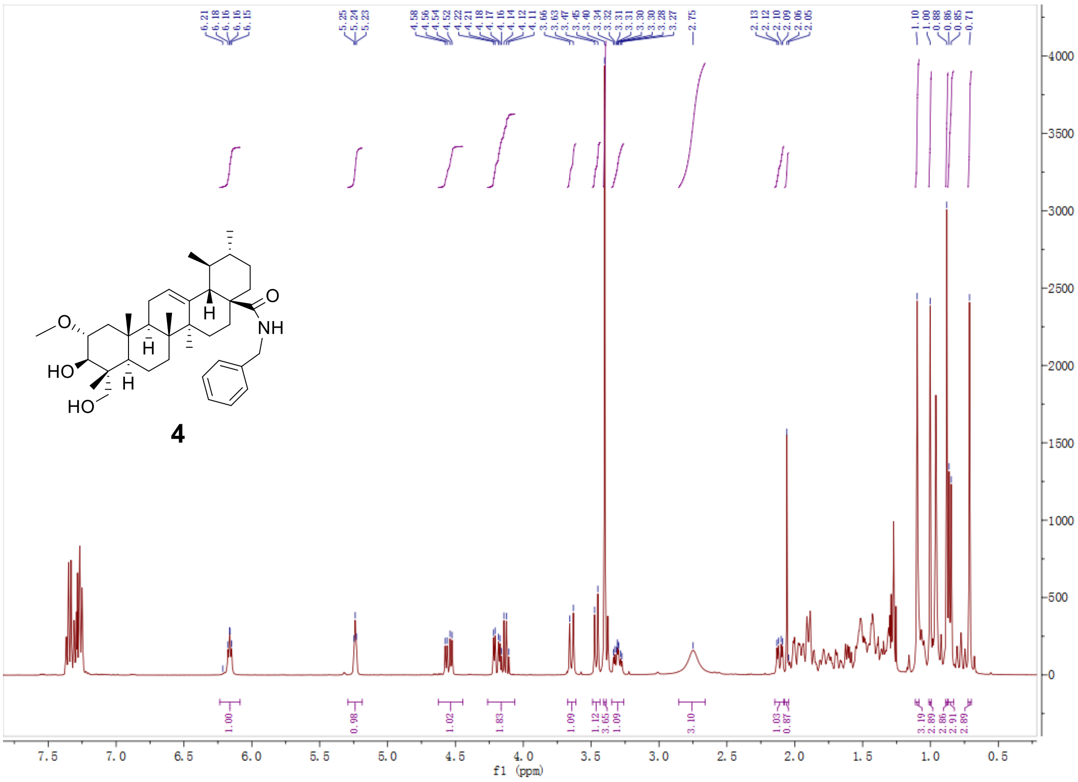

^{1}H NMR Spectrum of 2α-methoxy-3β,23-dihydroxy-urs-12-ene-28-benzylamide **(4)** (400 MHz, CDCl3)

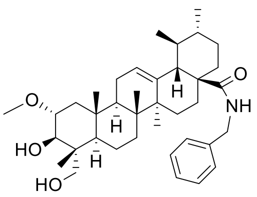

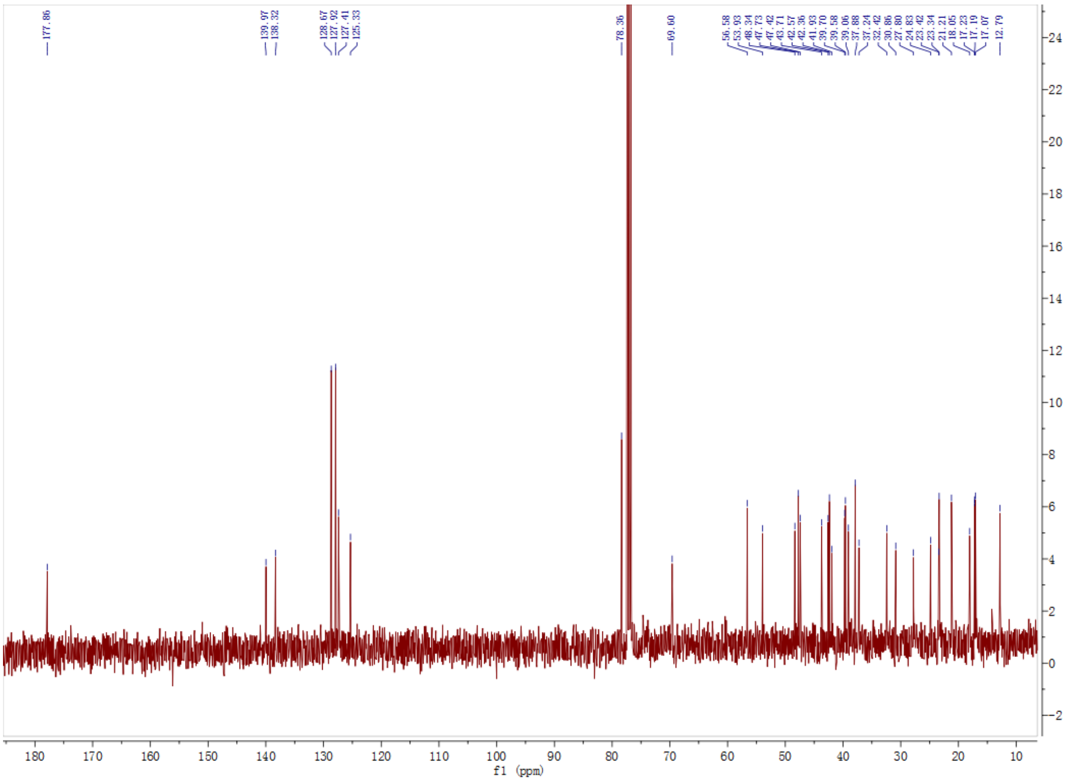

^{13}C NMR Spectrum of 2α-methoxy-3β,23-dihydroxy-urs-12-ene-28-benzylamide **(4)** (101 MHz, CDCl3)

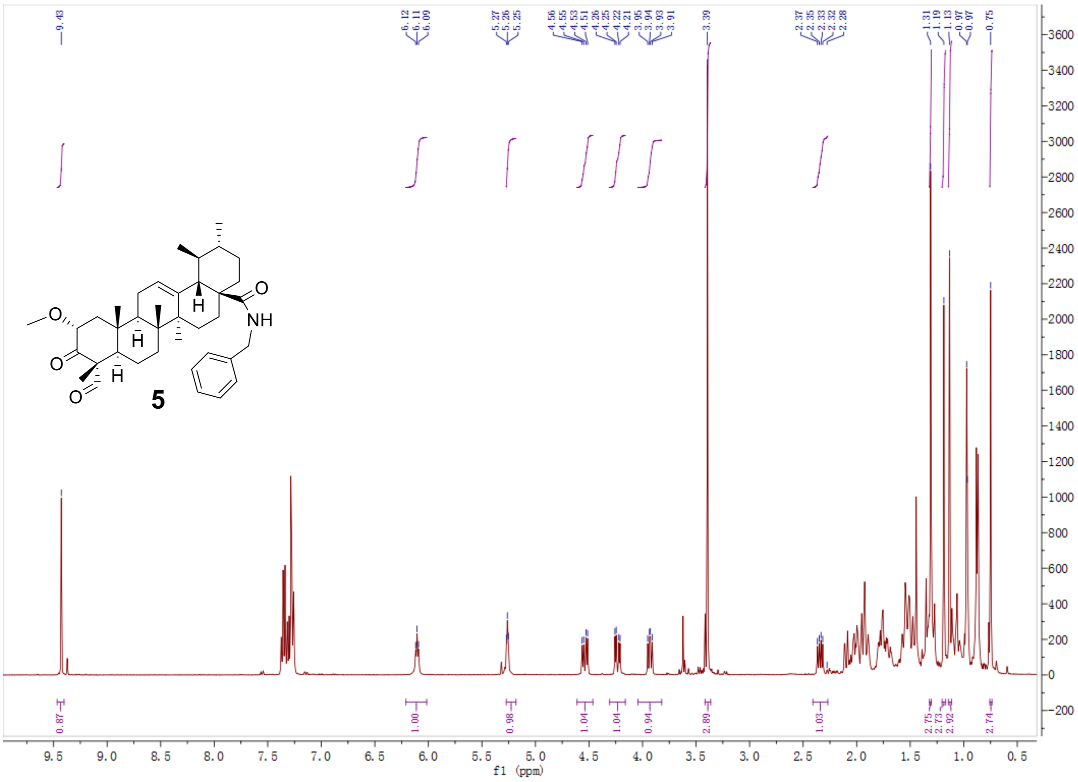

^{1}H NMR Spectrum of 2α-methoxy-3,23-dioxo-urs-12-ene-28-benzylamide **(5)** (400 MHz, CDCl3)

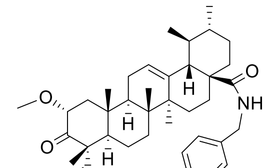

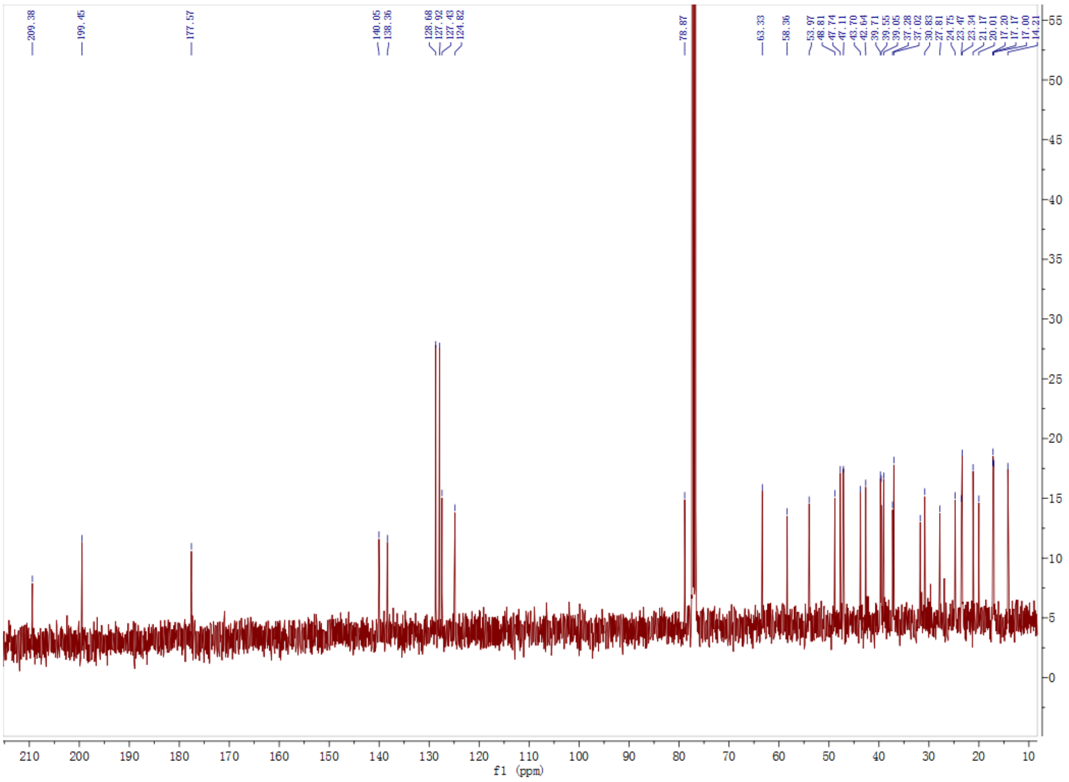

^{13}C NMR Spectrum of 2α-methoxy-3,23-dioxo-urs-12-ene-28-benzylamide **(5)** (101 MHz, CDCl3)

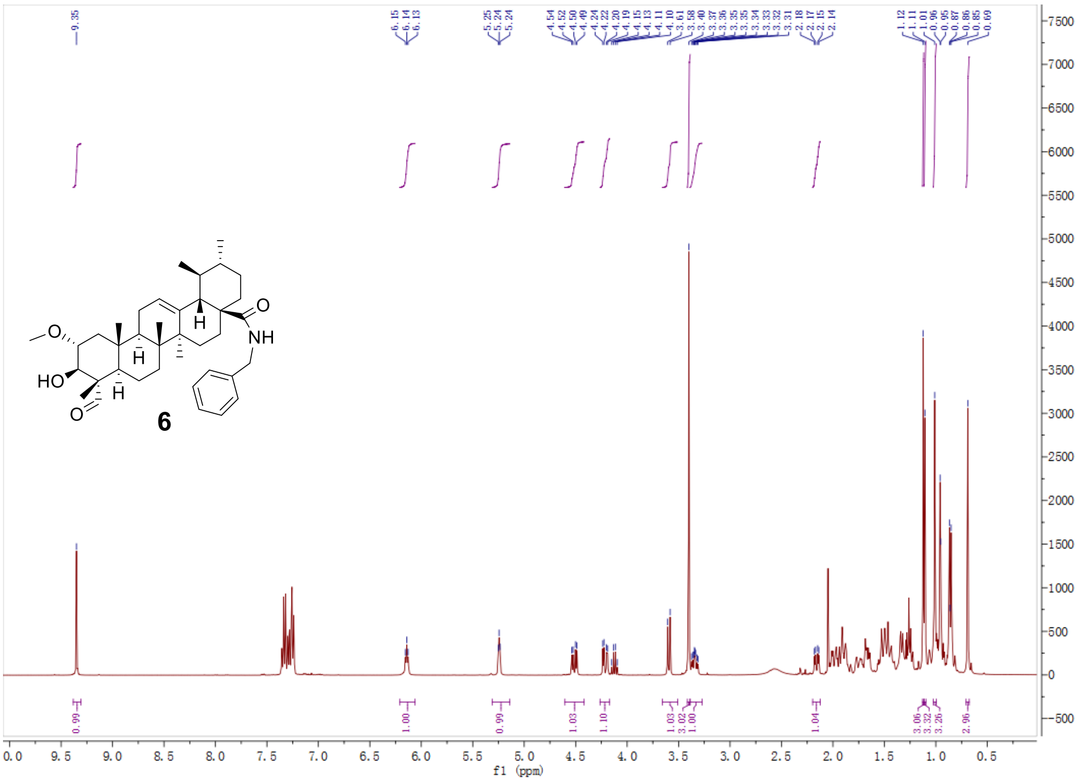

^{1}H NMR Spectrum of 2α-methoxy-3β-hydroxy-23-oxo-urs-12-ene-28-benzylamide **(6)** (400 MHz, CDCl3)

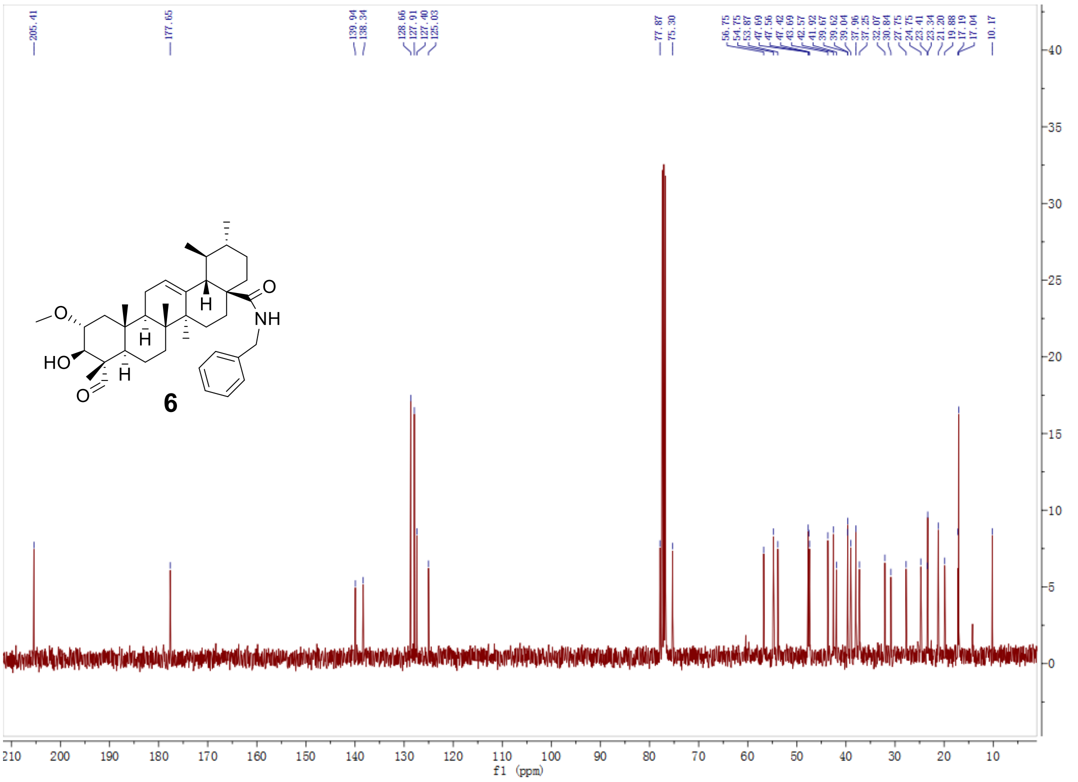

^{13}C NMR Spectrum of 2α-methoxy-3β-hydroxy-23-oxo-urs-12-ene-28-benzylamide **(6)** (101 MHz, CDCl3)

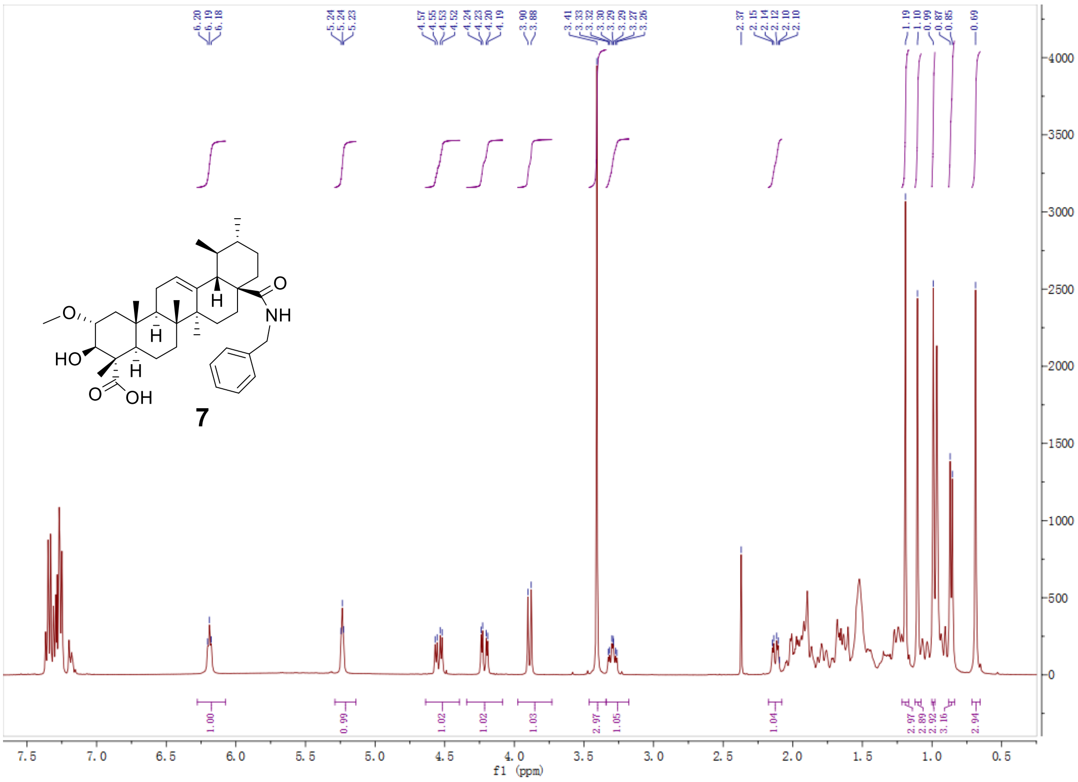

^{1}H NMR Spectrum of 2α-methoxy-3β-hydroxy-urs-12-ene-28-benzylamide-23-oic acid **(7)** (400 MHz, CDCl3)

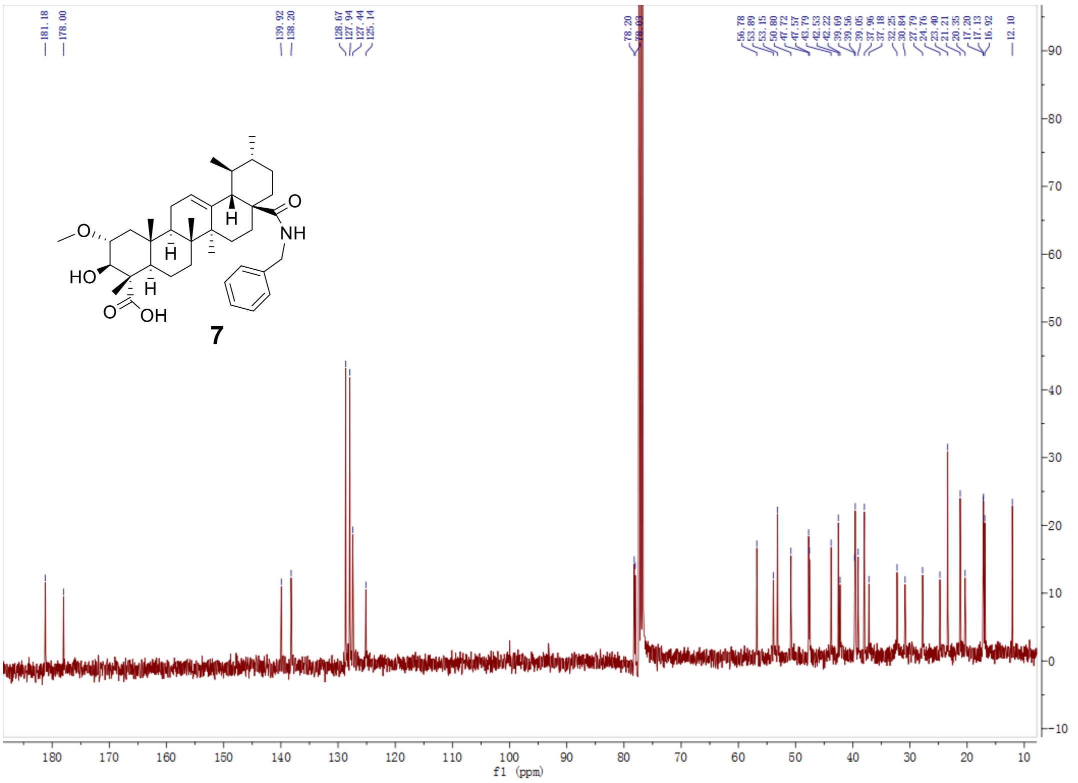

^{13}C NMR Spectrum of 2α-methoxy-3β-hydroxy-urs-12-ene-28-benzylamide-23-oic acid **(7)** (101 MHz, CDCl3)

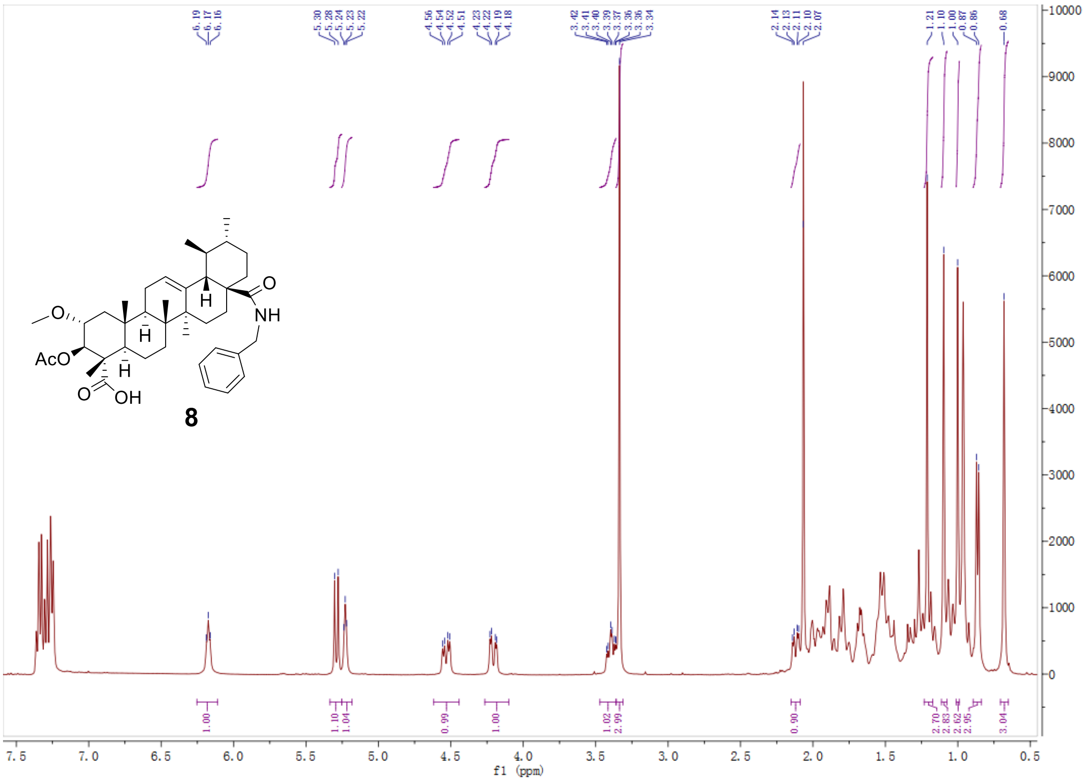

^{1}H NMR Spectrum of 2α-methoxy-3β- acetoxy-urs-12-ene-28-benzylamide-23-oic acid **(8)** (400 MHz, CDCl3)

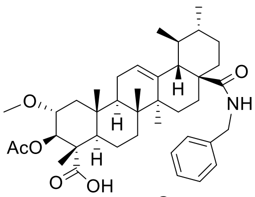

8

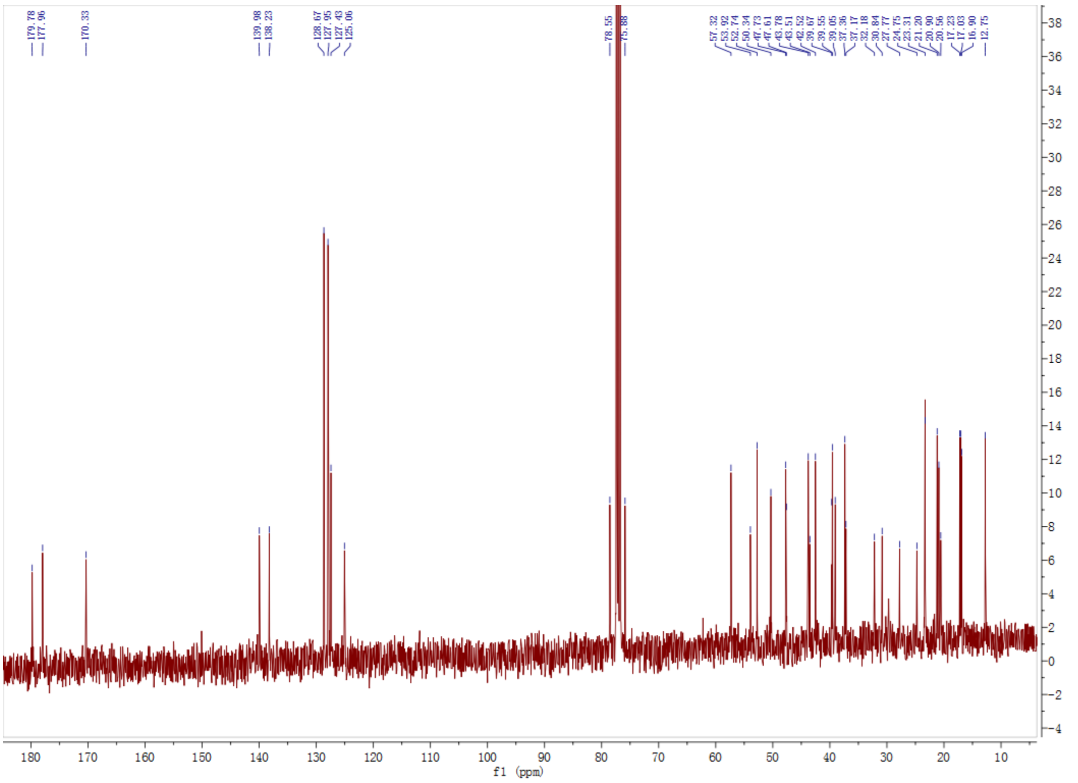

^{13}C NMR Spectrum of 2α-methoxy-3β- acetoxy-urs-12-ene-28-benzylamide-23-oic acid **(8)** (101 MHz, CDCl3)

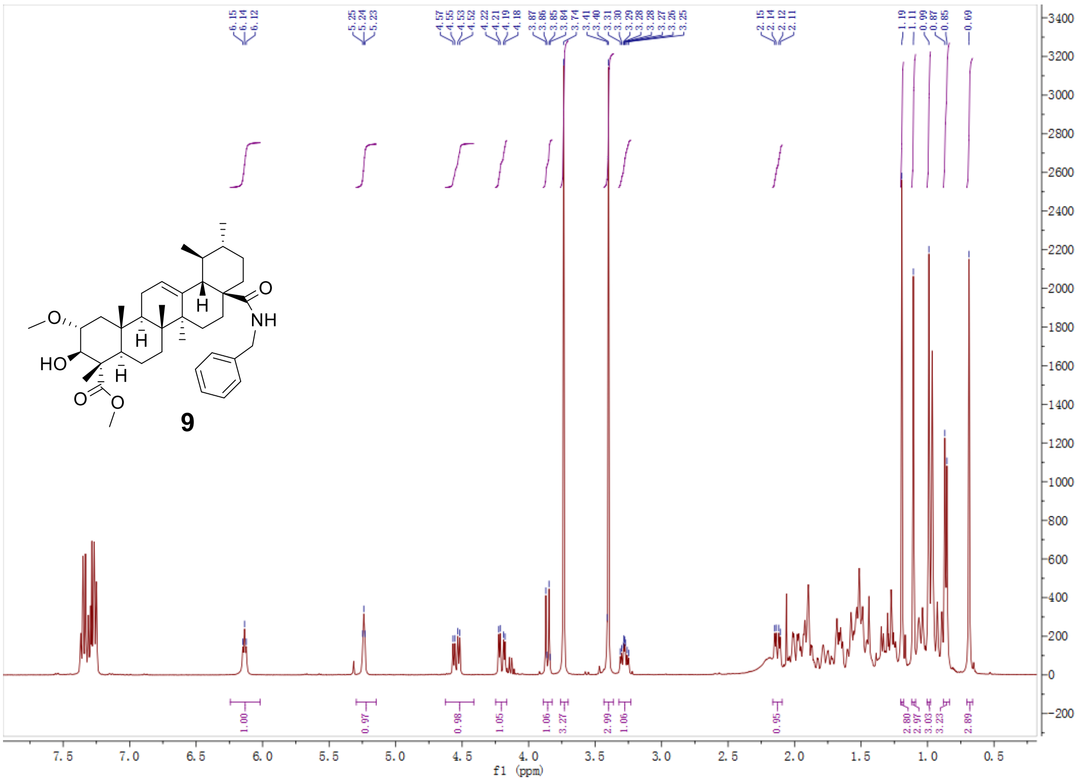

^{1}H NMR Spectrum of 2α-methoxy-3β-hydroxy-urs-12-ene-28-benzylamide-23-methly ester **(9)** (400 MHz, CDCl3)

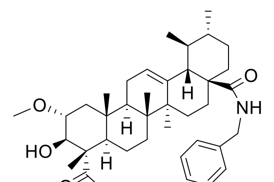

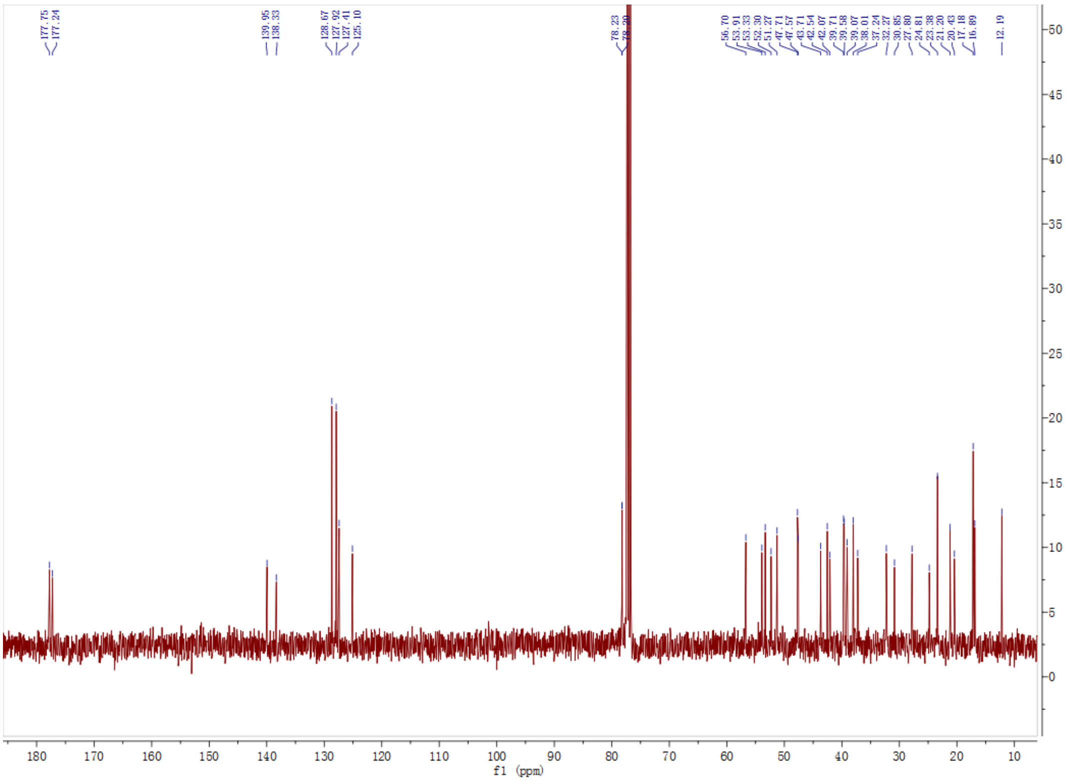

^{13}C NMR Spectrum of 2α-methoxy-3β-hydroxy-urs-12-ene-28-benzylamide-23-methly ester **(9)** (101 MHz, CDCl3)

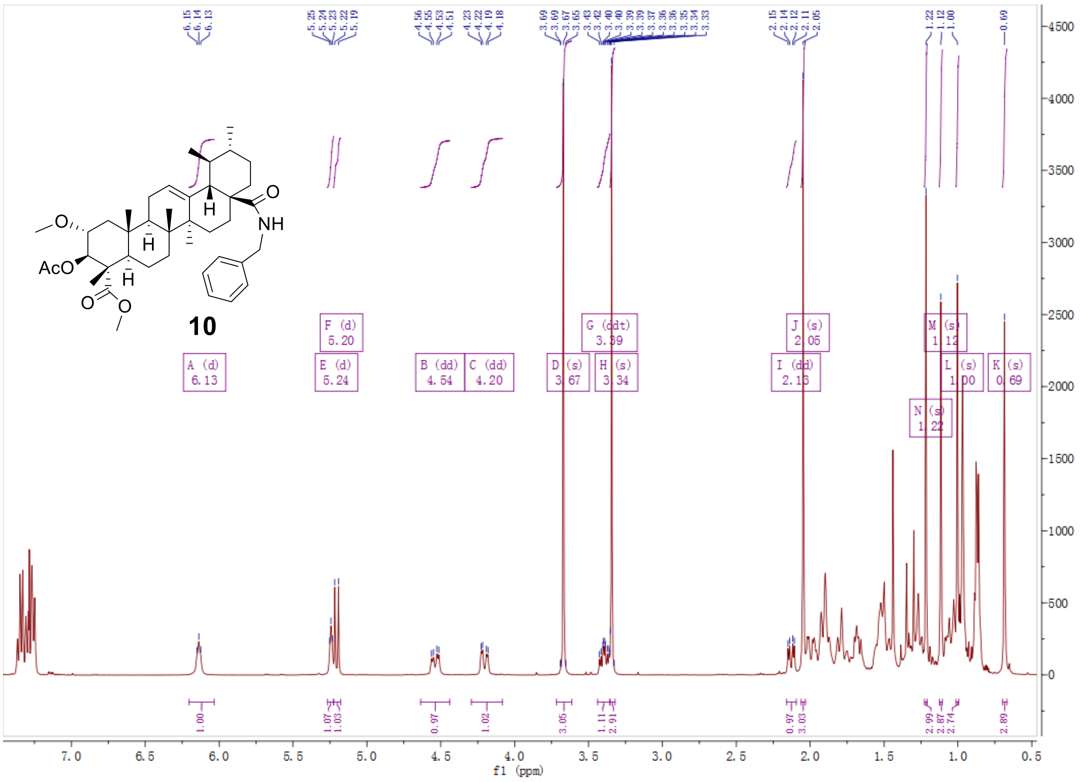

^{1}H NMR Spectrum of 2α-methoxy-3β- acetoxy-urs-12-ene-28-benzylamide-23-methly ester **(10)** (400 MHz, CDCl3)

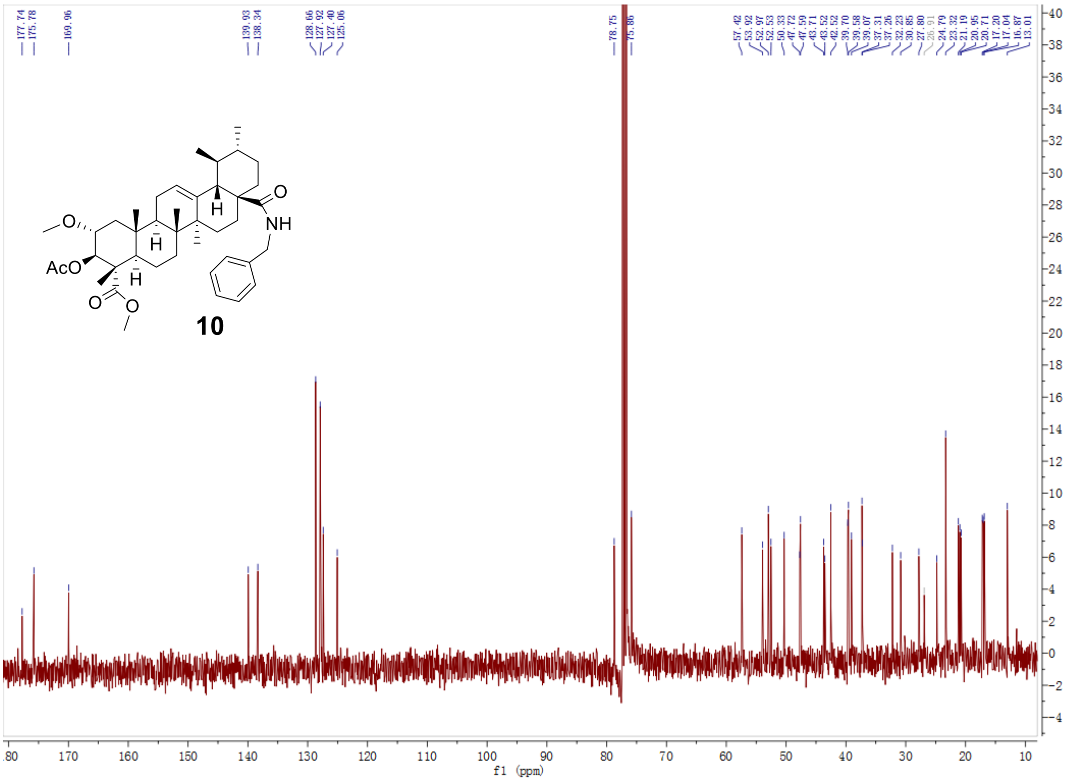

^{13}C NMR Spectrum of 2α-methoxy-3β- acetoxy-urs-12-ene-28-benzylamide-23-methly ester **(10)** (101 MHz, CDCl3)

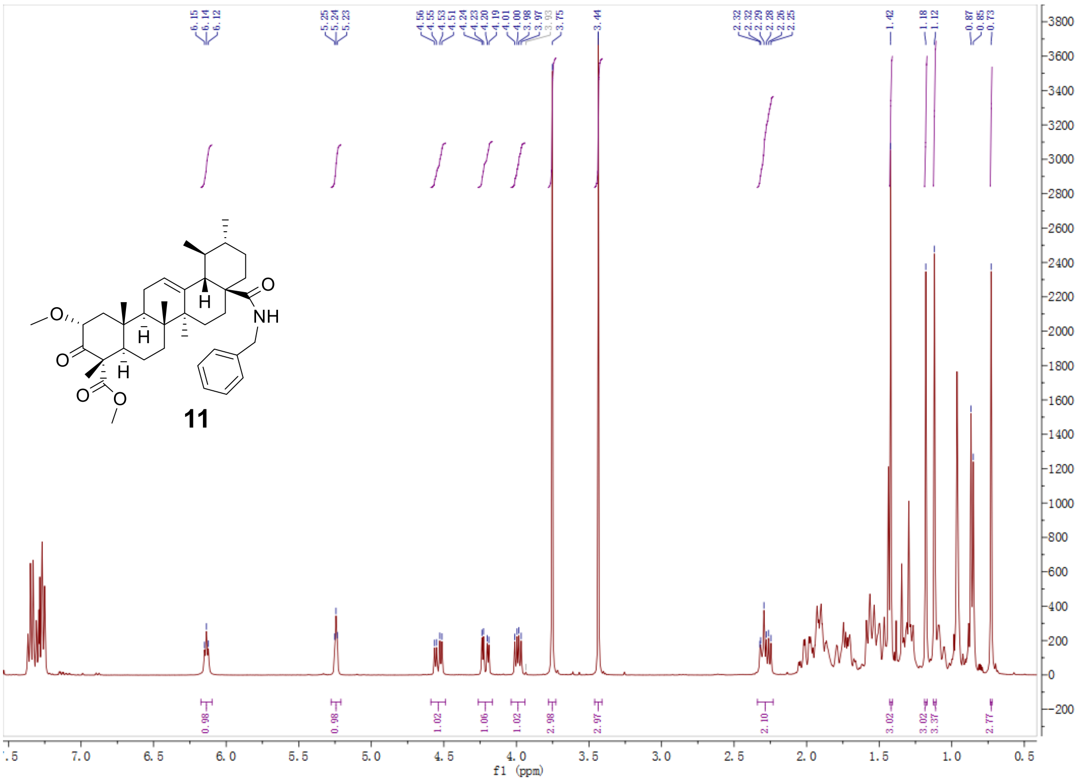

^{1}H NMR Spectrum of 2α-methoxy-3-oxo-urs-12-ene-28-benzyl amide-23-methly ester **(11)** (400 MHz, CDCl3)

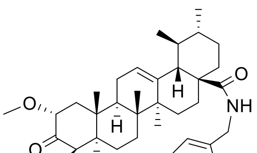

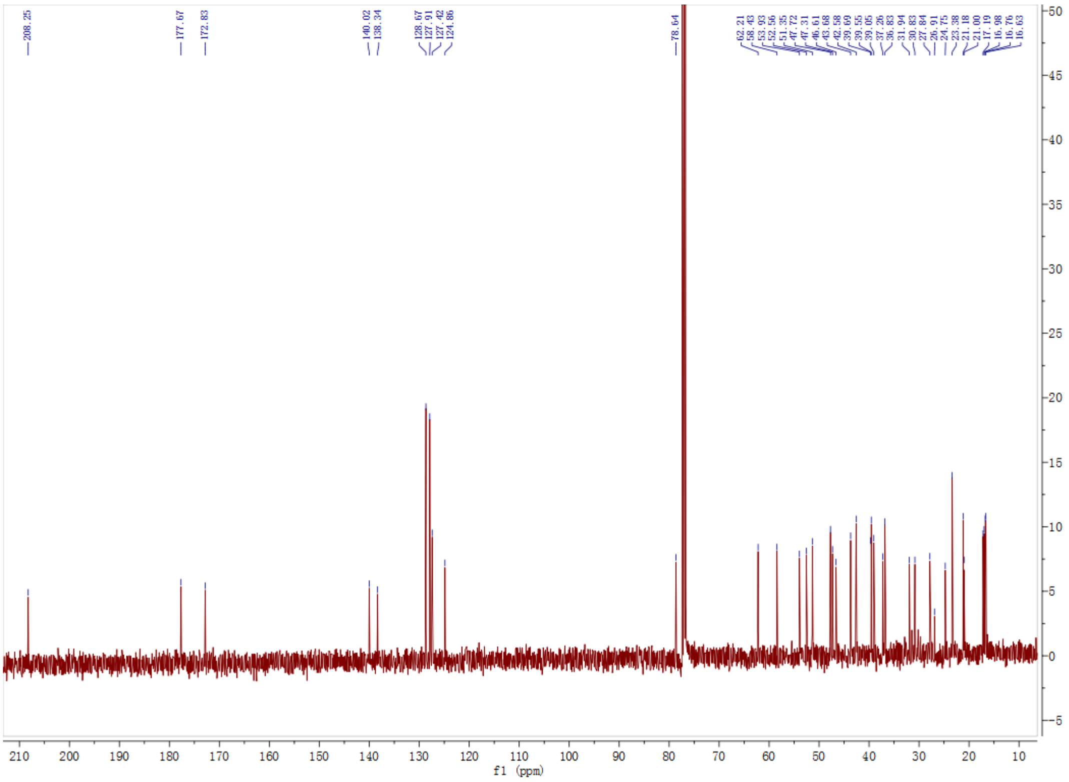

^{13}C NMR Spectrum of 2α-methoxy-3-oxo-urs-12-ene-28-benzyl amide-23-methly ester **(11)** (101 MHz, CDCl3)

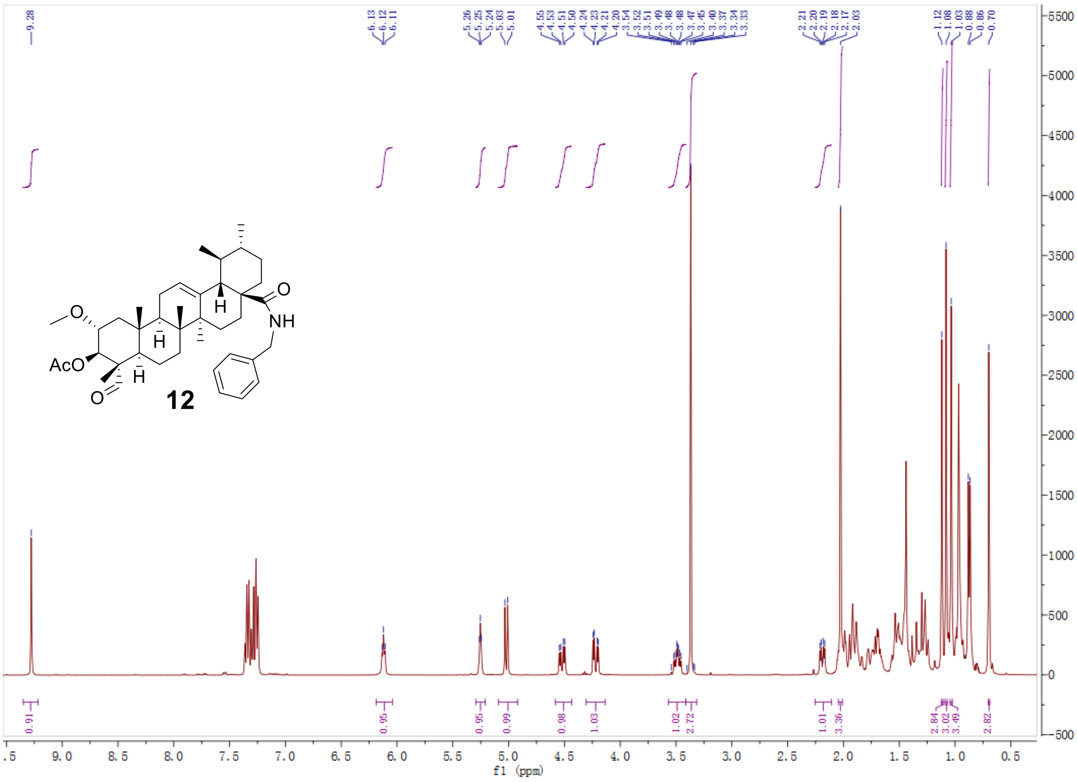

^{1}H NMR Spectrum of 2α-methoxy-3β-acetoxy-23-oxo-urs-12-ene-28-benzyl amide **(12)** (400 MHz, CDCl3)

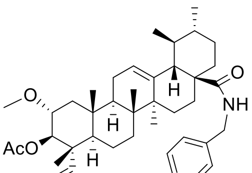

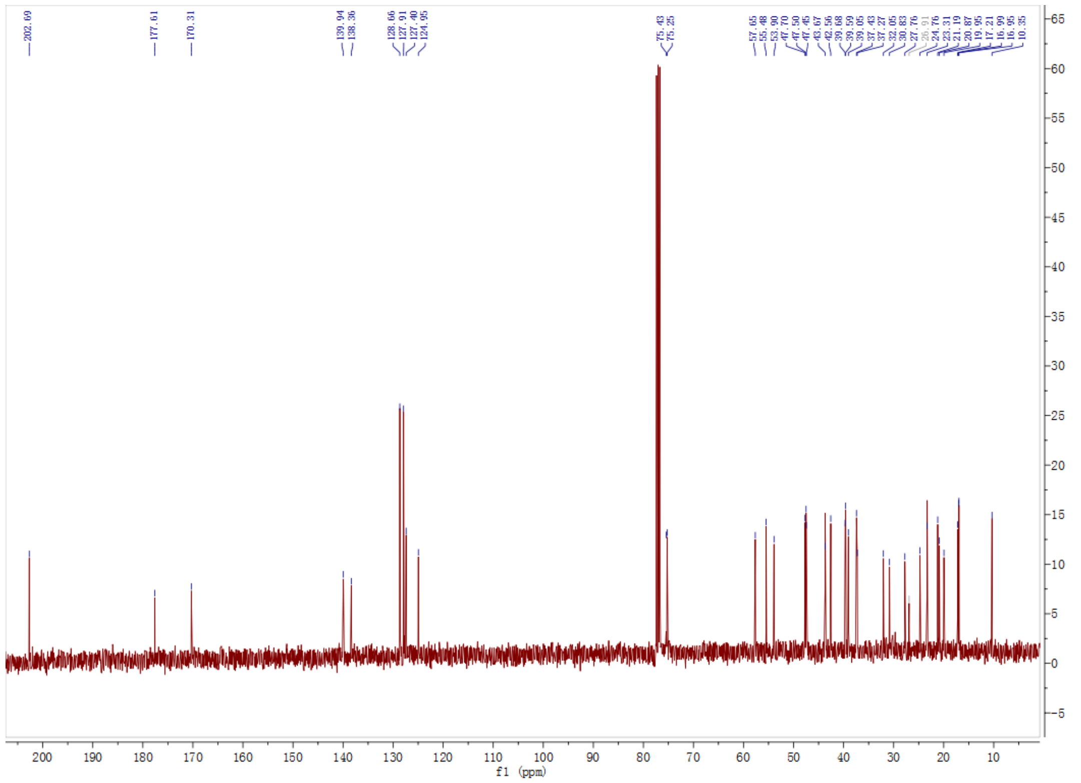

^{13}C NMR Spectrum of 2α-methoxy-3β-acetoxy-23-oxo-urs-12-ene-28-benzyl amide **(12)** (101 MHz, CDCl3)

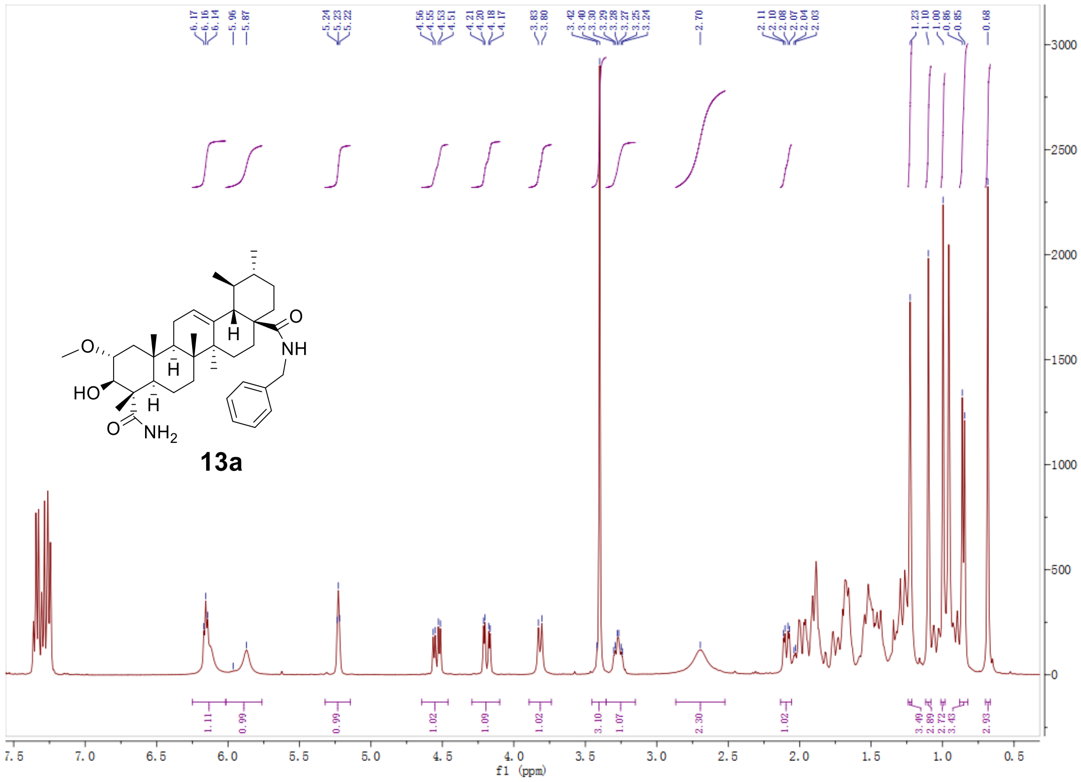

^{1}H NMR Spectrum of 2α-methoxy-3β-hydroxy-urs-12-ene-28-benzyl amide-23-amide **(13a)** (400 MHz, CDCl3)

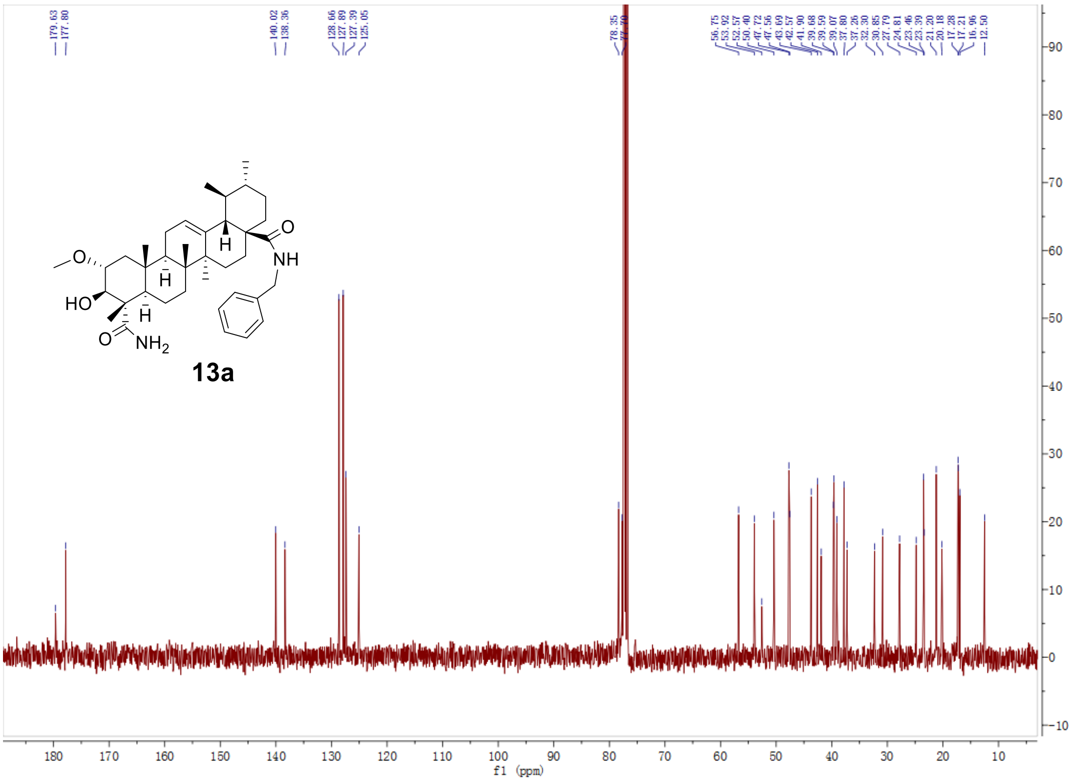

^{13}C NMR Spectrum of 2α-methoxy-3β-hydroxy-urs-12-ene-28-benzyl amide-23-amide **(13a)** (101 MHz, CDCl3)

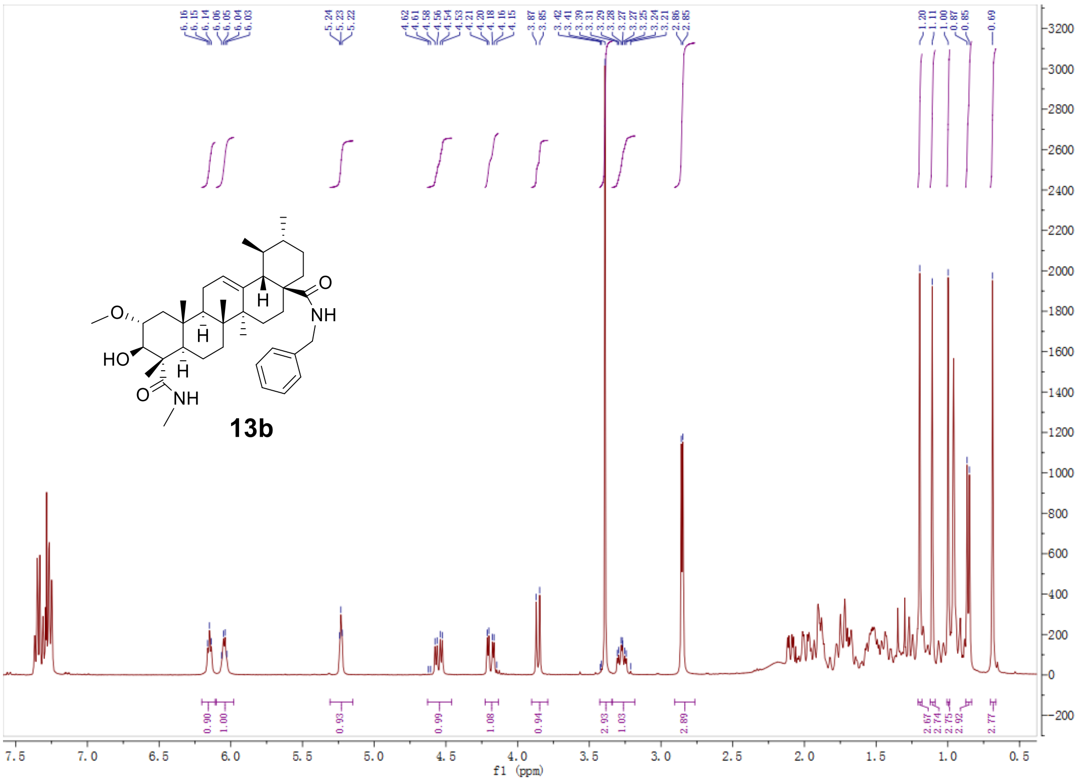

^{1}H NMR Spectrum of 2α-methoxy-3β-hydroxy-urs-12-ene-28-benzyl amide-23-methlyamide (**13b**) (400 MHz, CDCl3)

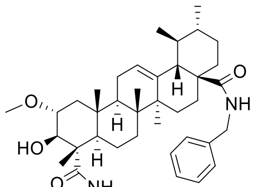

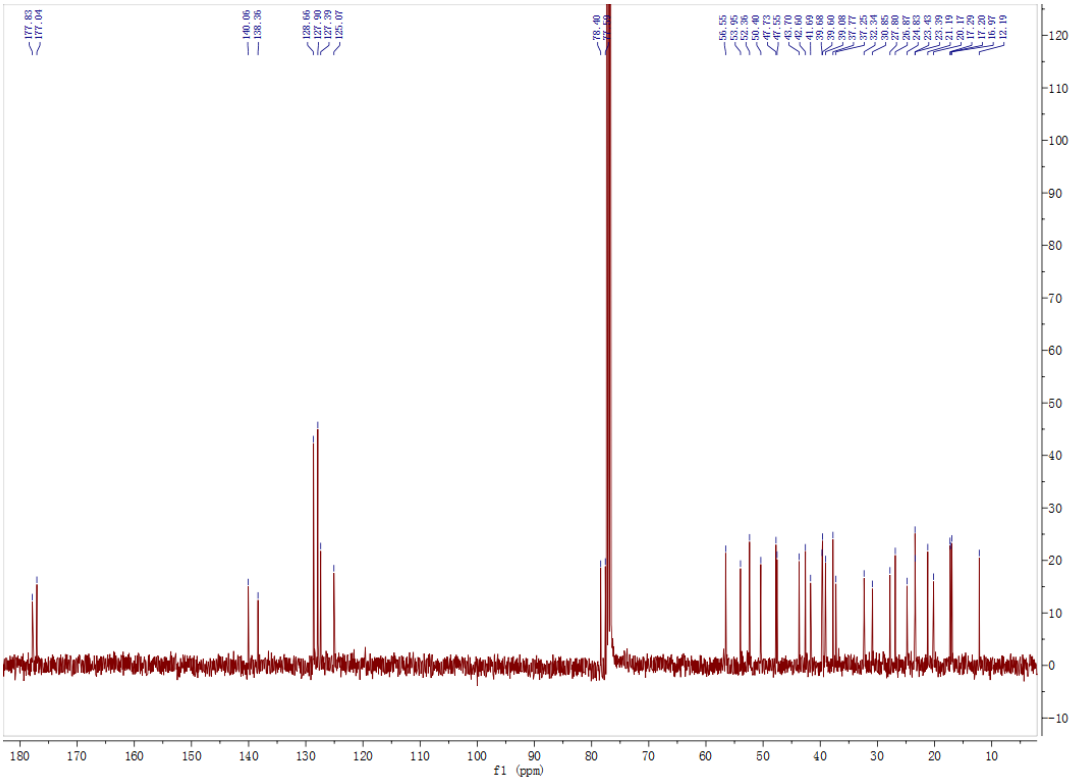

^{13}C NMR Spectrum of 2α-methoxy-3β-hydroxy-urs-12-ene-28-benzyl amide-23-methlyamide (**13b**) (101 MHz, CDCl3)

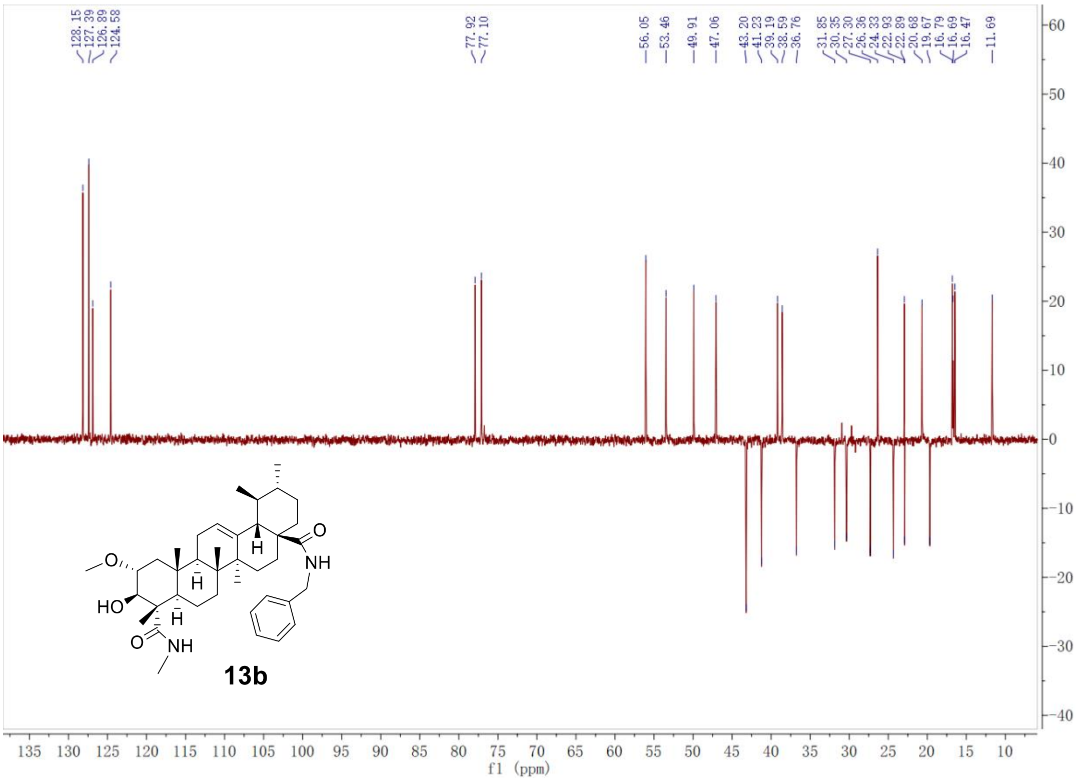

DEPT135 NMR Spectrum of 2α-methoxy-3β-hydroxy-urs-12-ene-28-benzyl amide-23-methlyamide (**13b**) (126 MHz, CDCl3)

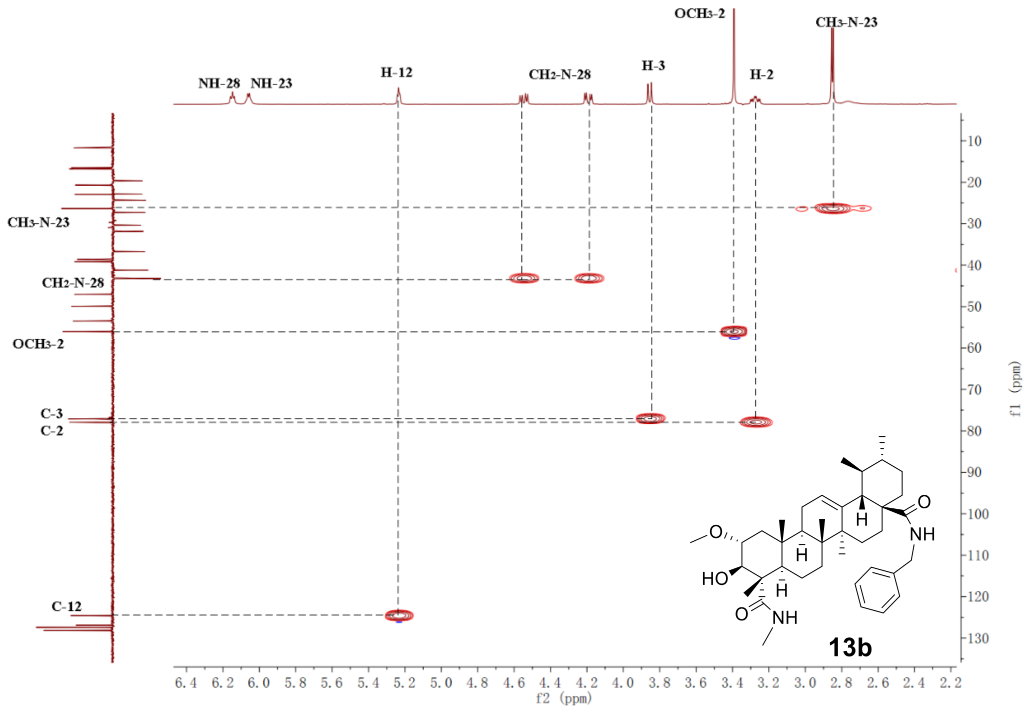

2-D ^{1}H-^{13}C HSQC NMR Spectrum of 2α-methoxy-3β-hydroxy-urs-12-ene-28-benzyl amide-23-methlyamide (**13b**) (^{1}H NMR 500 MHz, $CDCl_3$, ^{13}C NMR 126 MHz, $CDCl_3$)

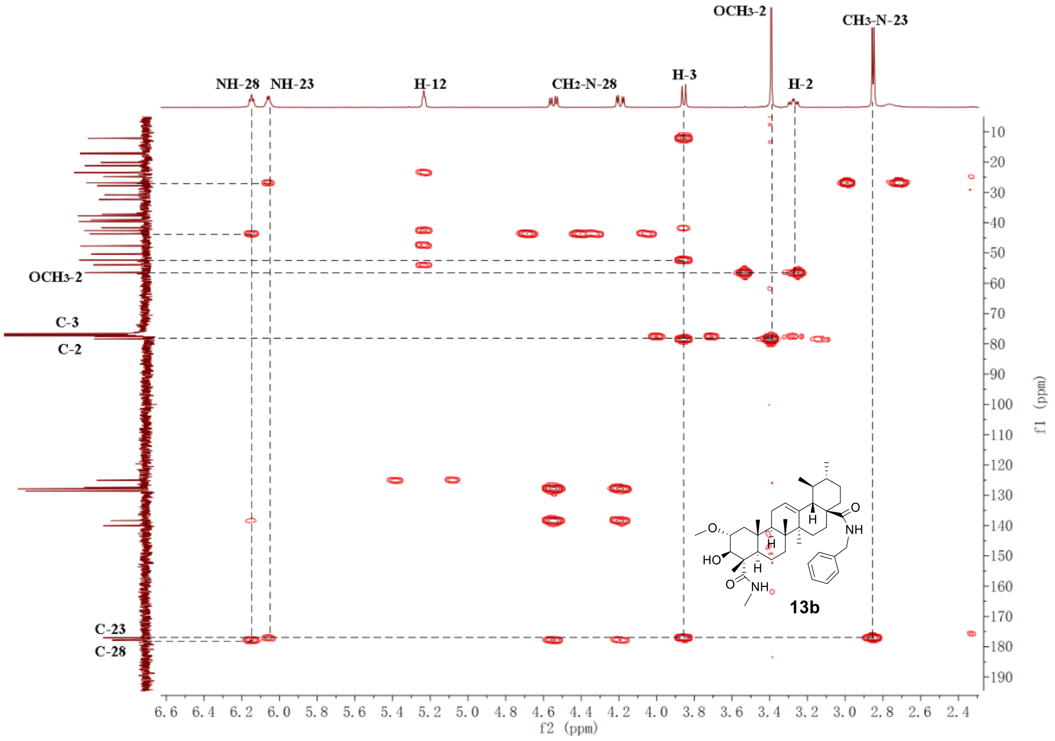

2-D ^{1}H-^{13}C HMBC NMR Spectrum of 2α-methoxy-3β-hydroxy-urs-12-ene-28-benzyl

amide-23-methlyamide (**13b**) (^{1}H NMR 500 MHz, $CDCl_3$, ^{13}C NMR 126 MHz, $CDCl_3$)

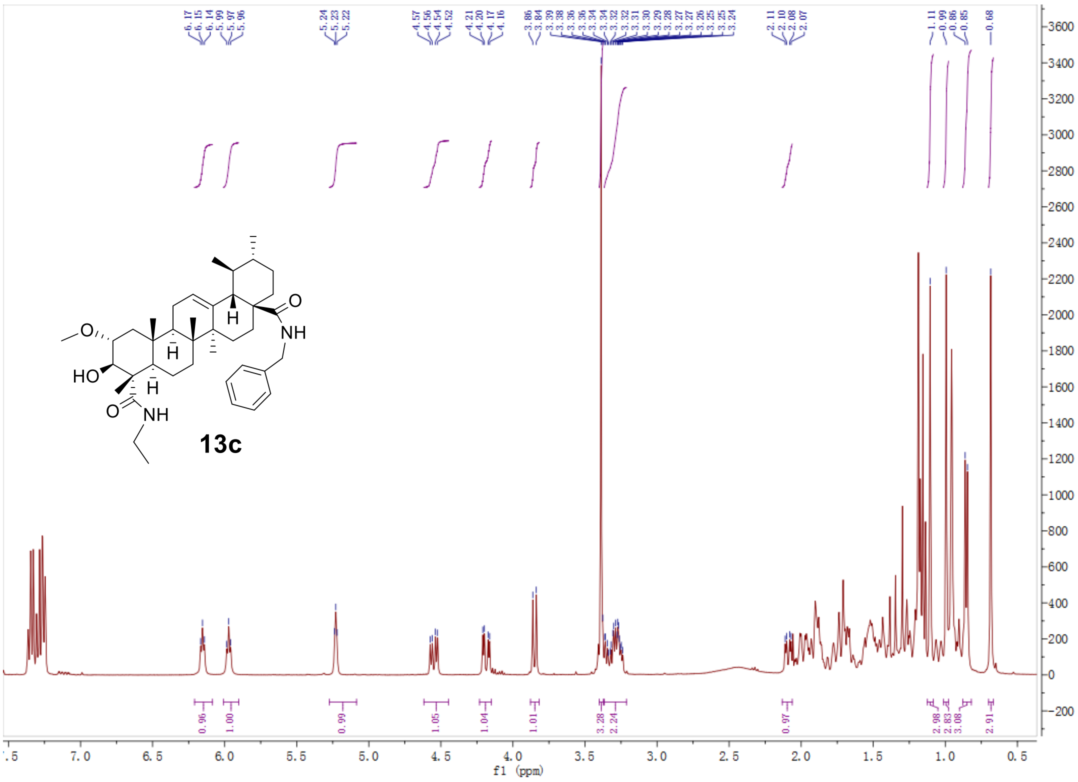

^{1}H NMR Spectrum of 2α-methoxy-3β-hydroxy-urs-12-ene-28-benzyl amide-23-ethlyamide (**13c**) (400 MHz, CDCl3)

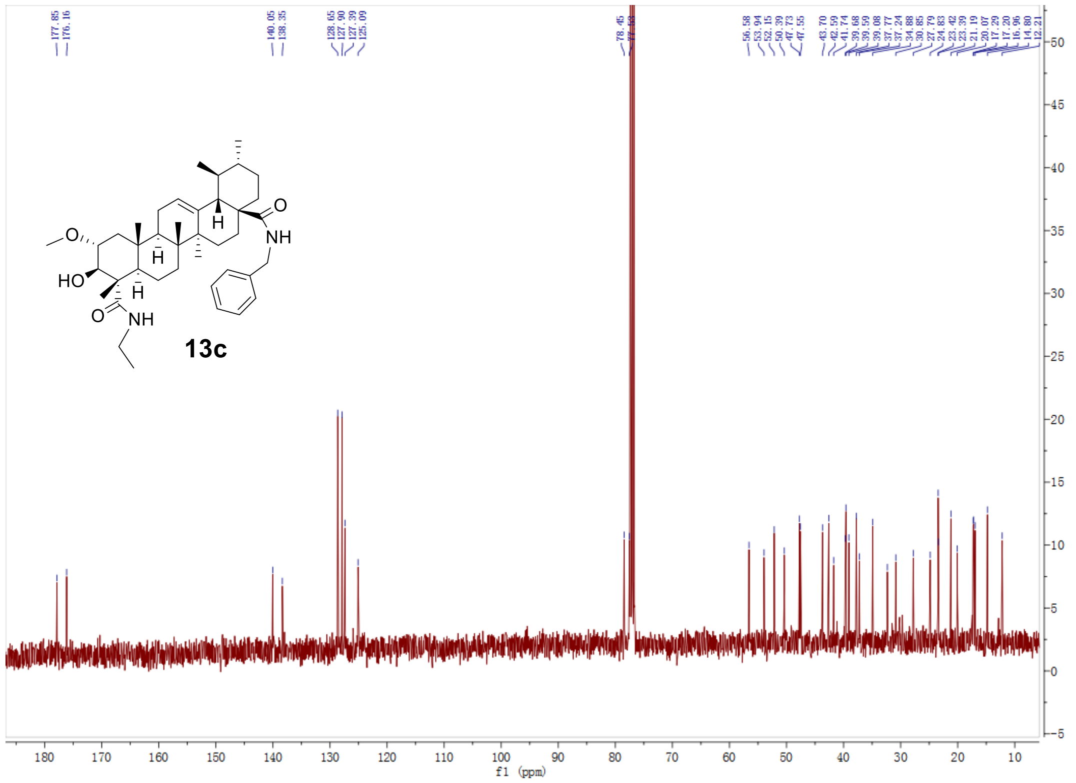

^{13}C NMR Spectrum of 2α-methoxy-3β-hydroxy-urs-12-ene-28-benzyl amide-23-ethlyamide (**13c**) (101 MHz, CDCl3)

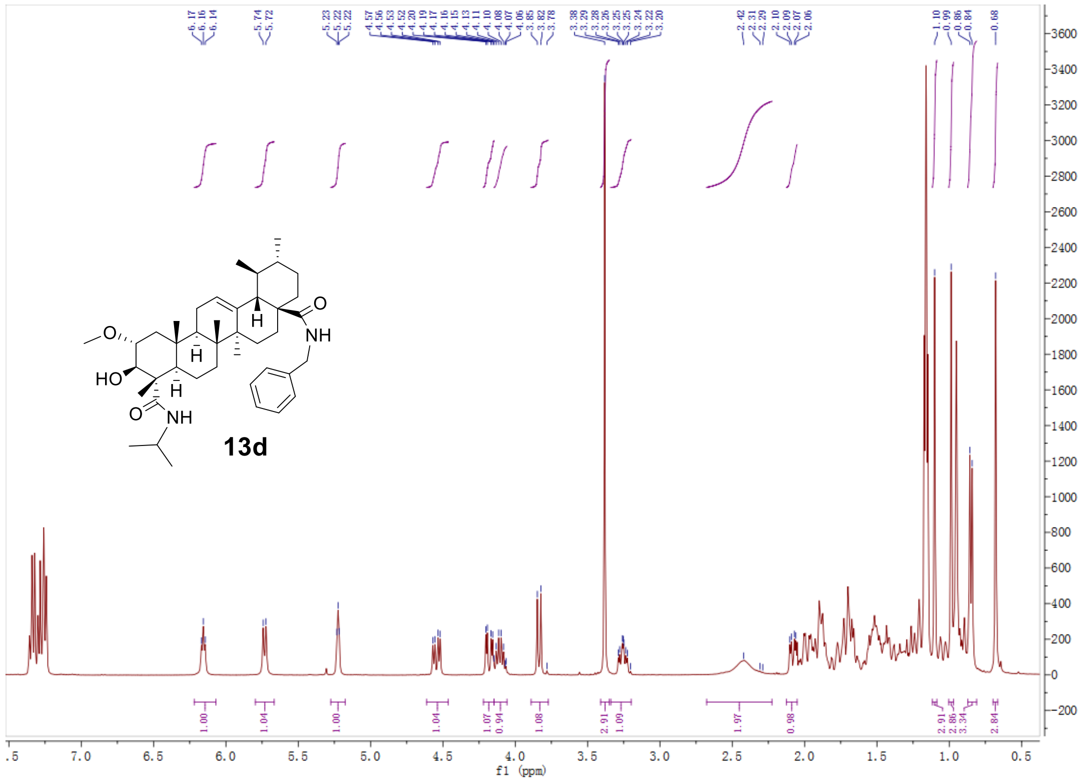

^{1}H NMR Spectrum of 2α-methoxy-3β-hydroxy-urs-12-ene-28-benzyl amide-23-isopropylamide (**13d**) (400 MHz, CDCl3)

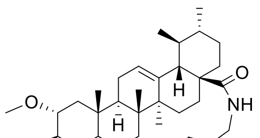

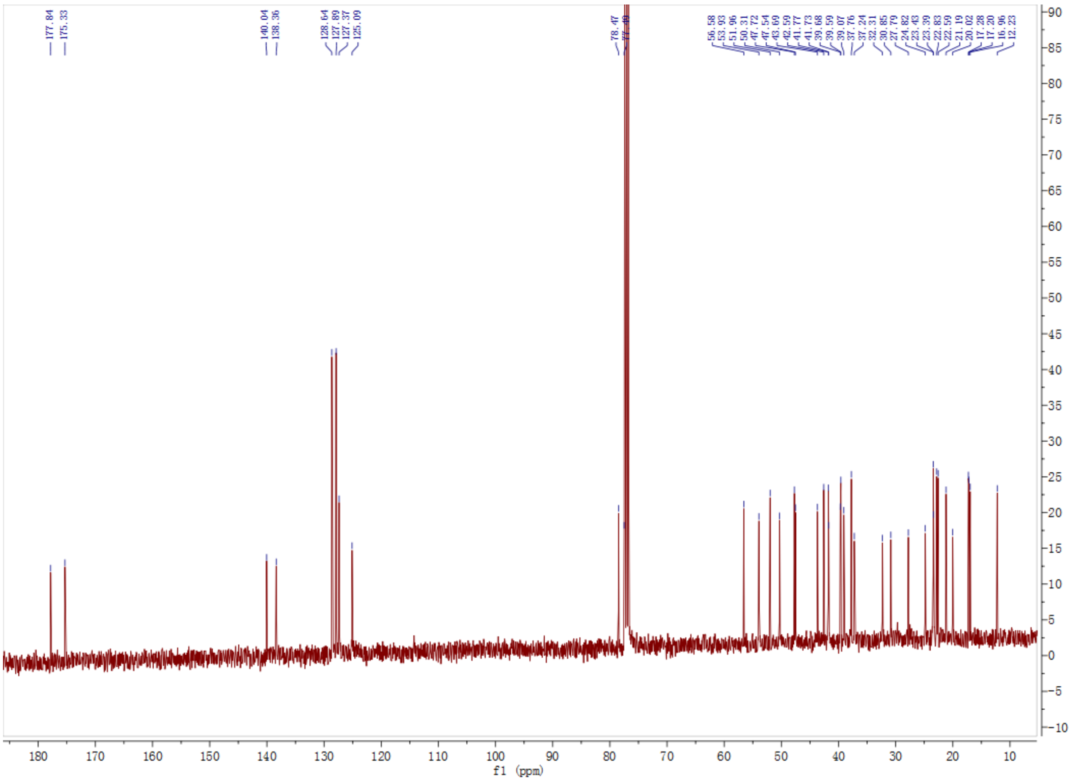

^{13}C NMR Spectrum of 2α-methoxy-3β-hydroxy-urs-12-ene-28-benzyl amide-23-isopropylamide (**13d**) (101 MHz, CDCl3)

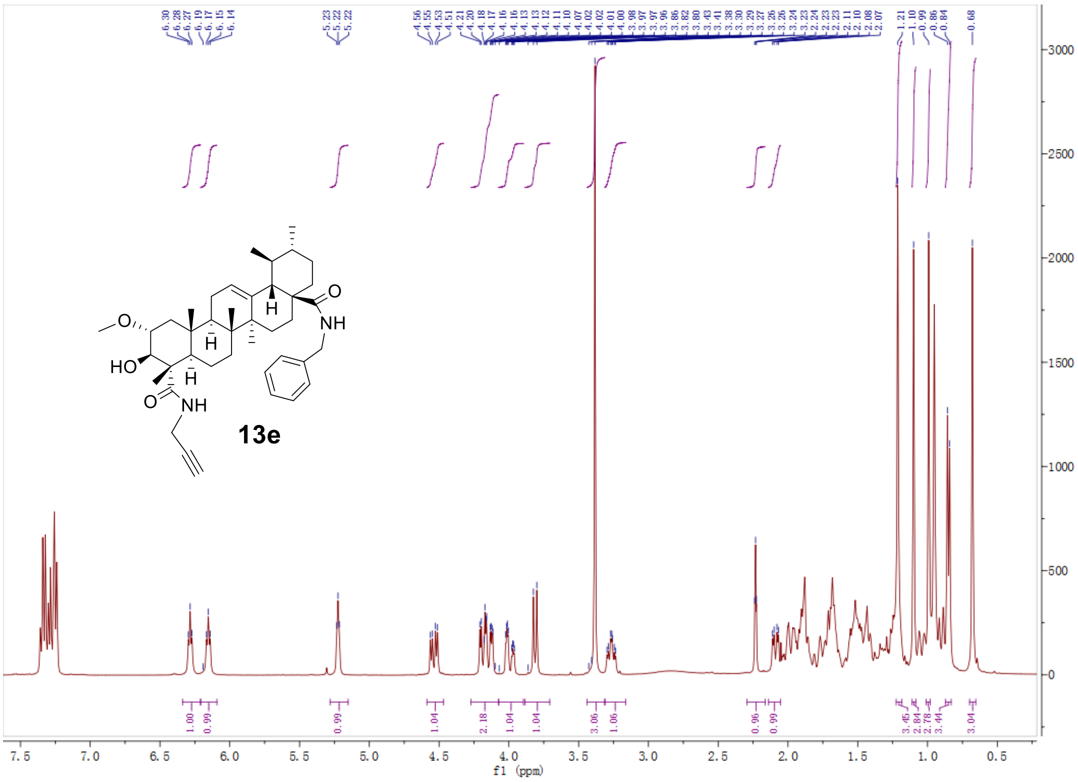

^{1}H NMR Spectrum of 2α-methoxy-3β-hydroxy-urs-12-ene-28-benzyl amide-23-propargylamide (**13e**) (400 MHz, CDCl3)

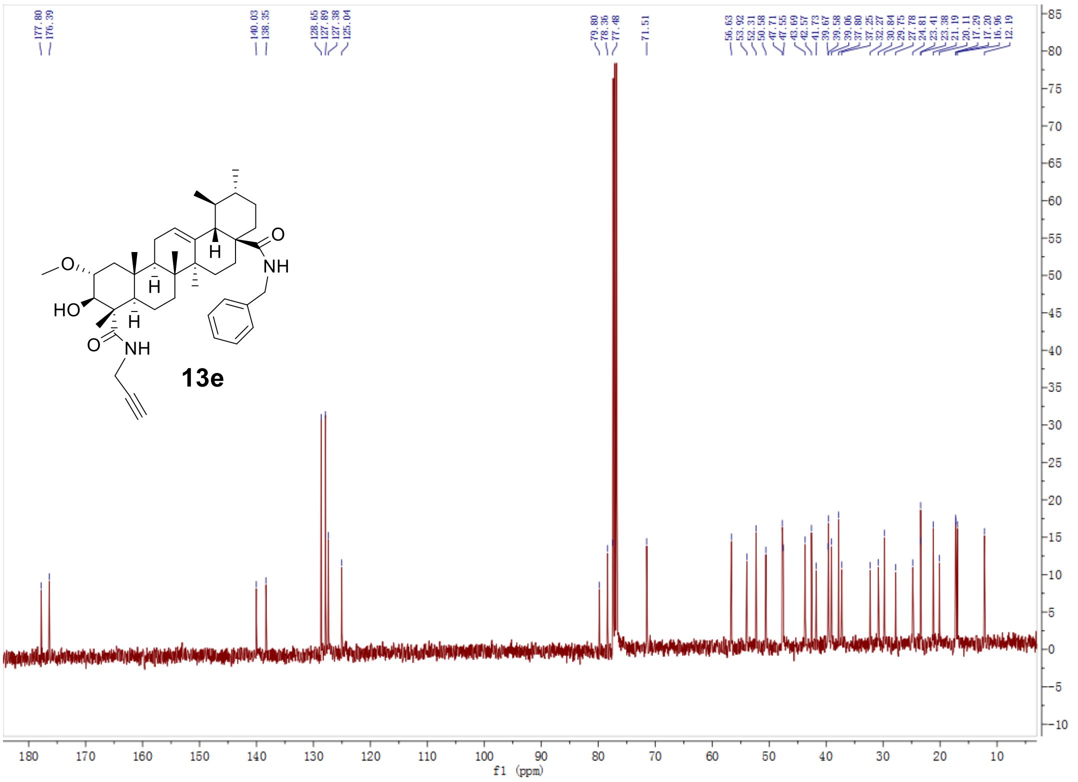

^{13}C NMR Spectrum of 2α-methoxy-3β-hydroxy-urs-12-ene-28-benzyl amide-23-propargylamide (**13e**) (101 MHz, CDCl3)

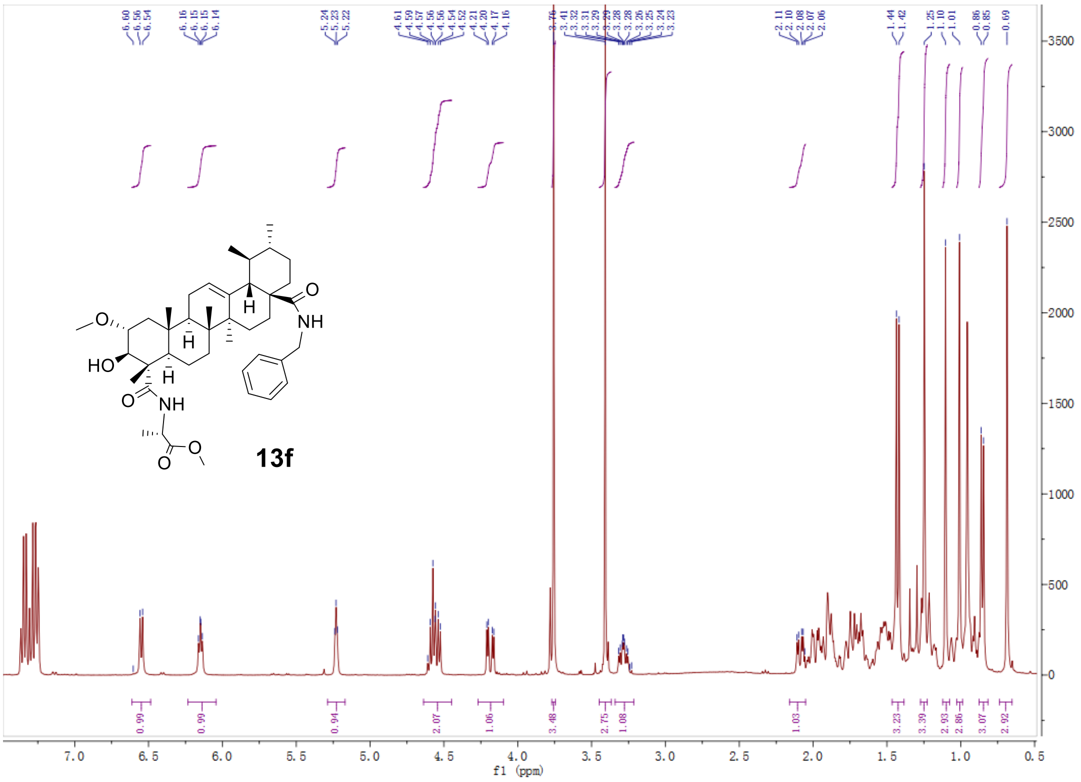

^{1}H NMR Spectrum of N-(2α-methoxy-3β-hydroxy-urs-12-ene-28-benzylamide-23-oyl)-L-alanine methyl ester (**13f**) (400 MHz, CDCl3)

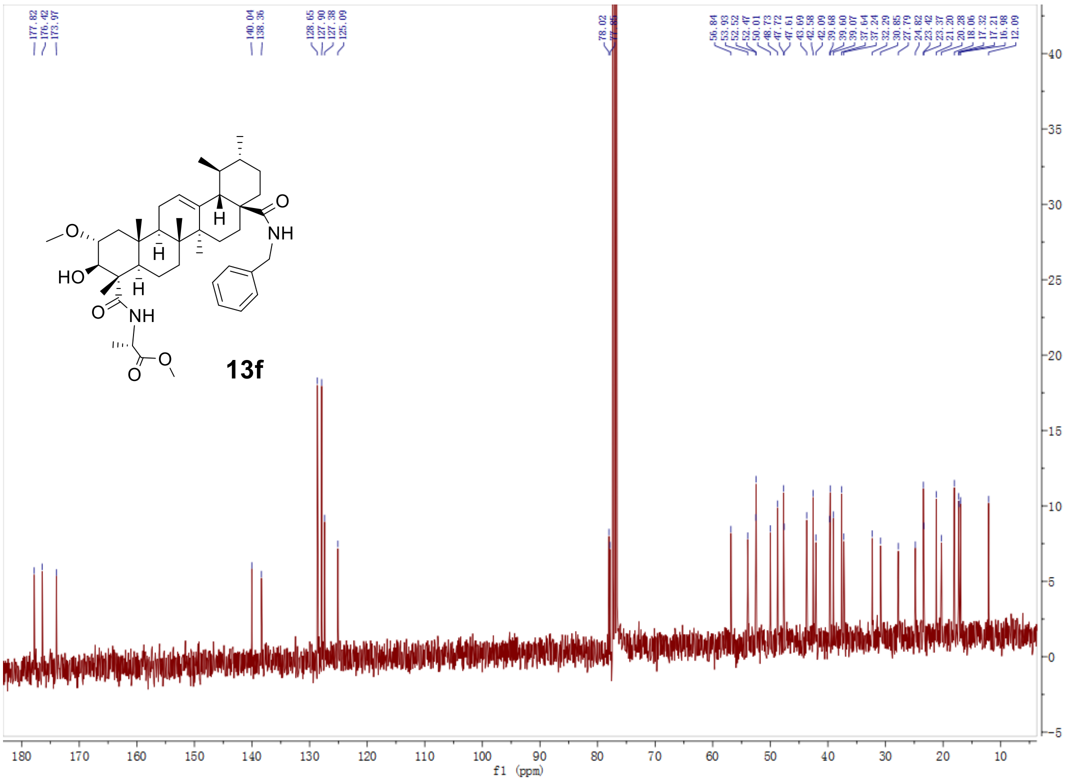

^{13}C NMR Spectrum of N-(2α-methoxy-3β-hydroxy-urs-12-ene-28-benzylamide-23-oyl)-L-alanine methyl ester (**13f**) (101 MHz, CDCl3)

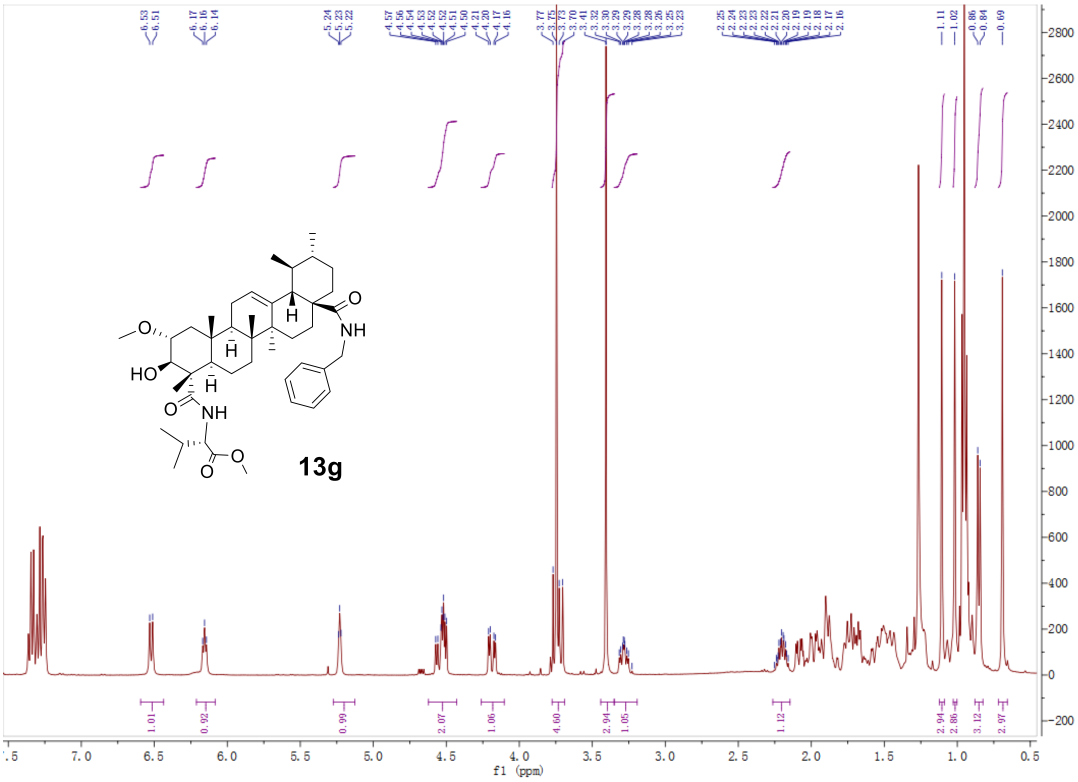

^{1}H NMR Spectrum of N-(2α-methoxy-3β-hydroxy-urs-12-ene-28-benzylamide-23-oyl)-L-valine methyl ester (**13g**) (400 MHz, CDCl3)

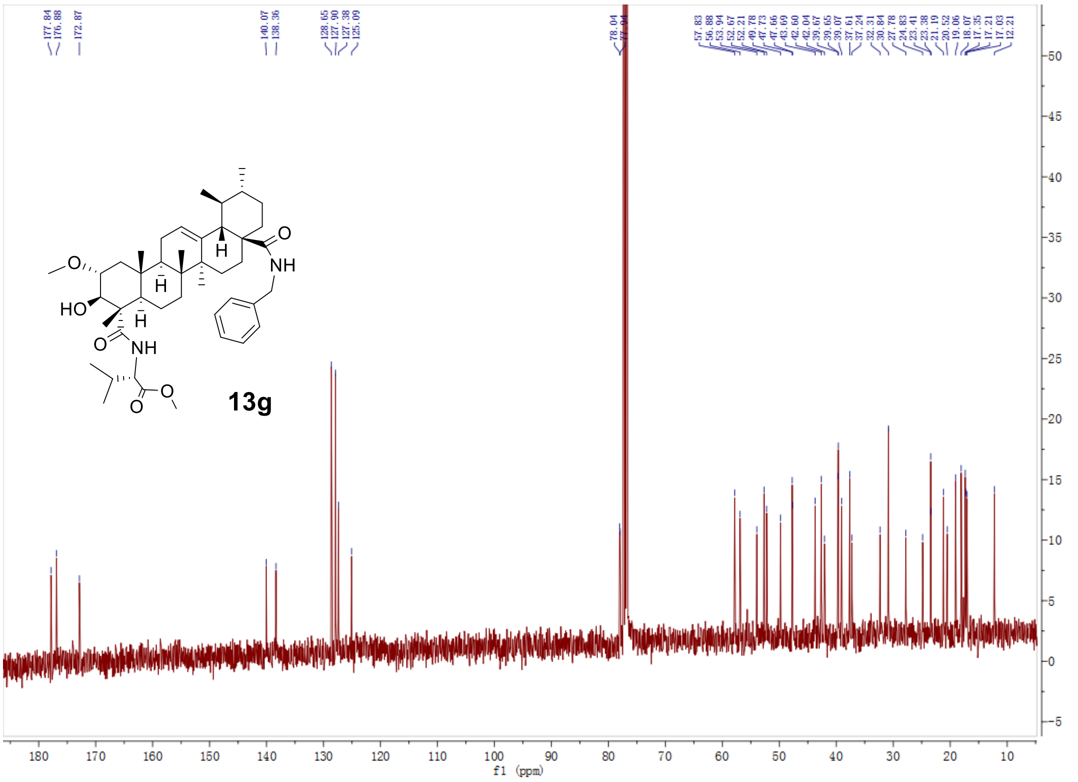

^{13}C NMR Spectrum of N-(2α-methoxy-3β-hydroxy-urs-12-ene-28-benzylamide-23-oyl)-L-valine methyl ester (**13g**) (101 MHz, CDCl3)

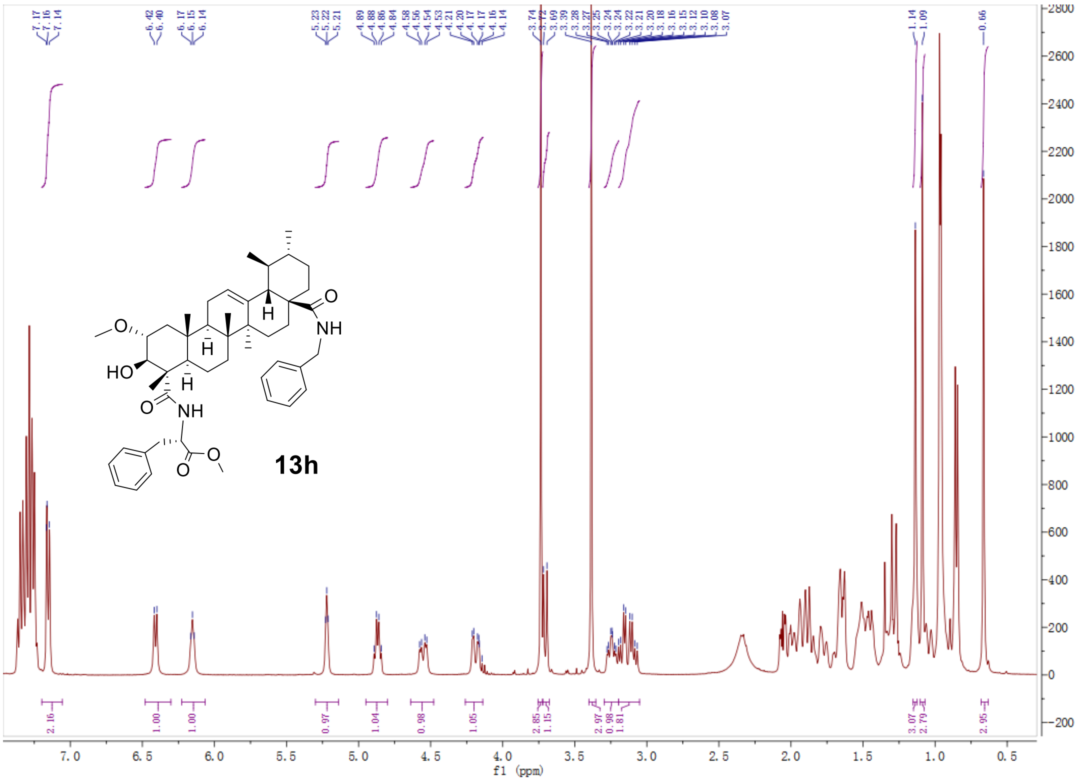

^{1}H NMR Spectrum of N-(2α-methoxy-3β-hydroxy-urs-12-ene-28-benzylamide-23-oyl)- L-phenylalanine methyl ester (**13h**) (400 MHz, CDCl3)

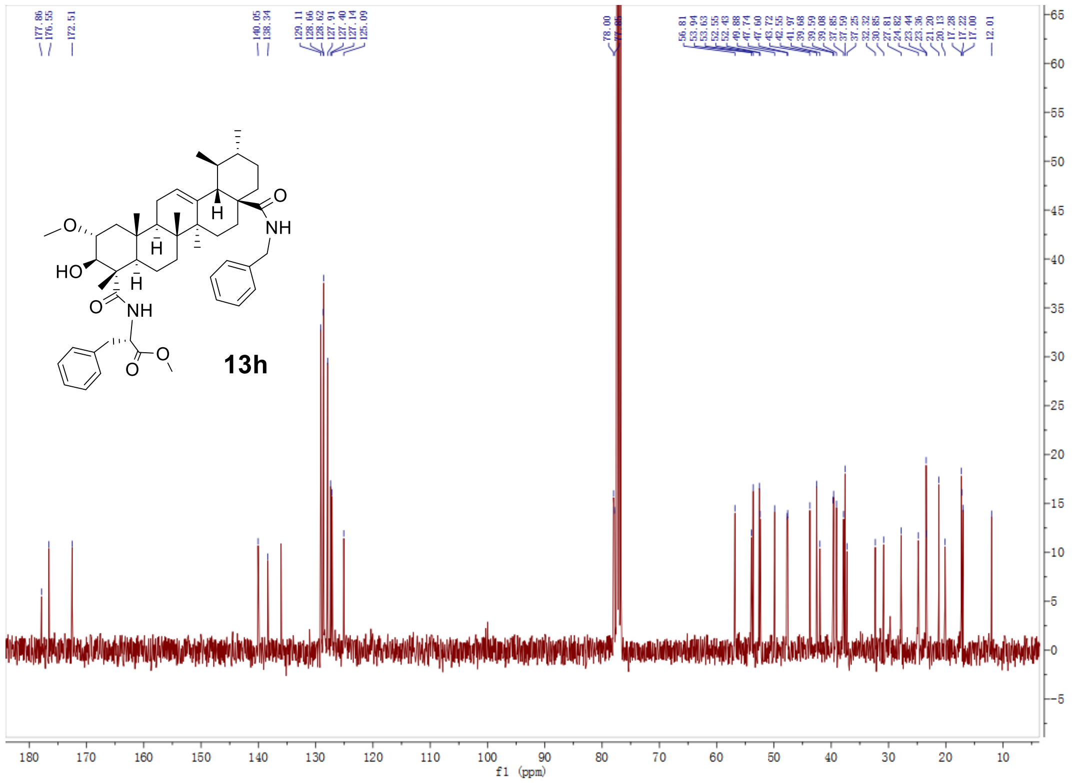

^{13}C NMR Spectrum of N-(2α-methoxy-3β-hydroxy-urs-12-ene-28-benzylamide-23-oyl)- L-phenylalanine methyl ester (**13h**) (101 MHz, CDCl3)

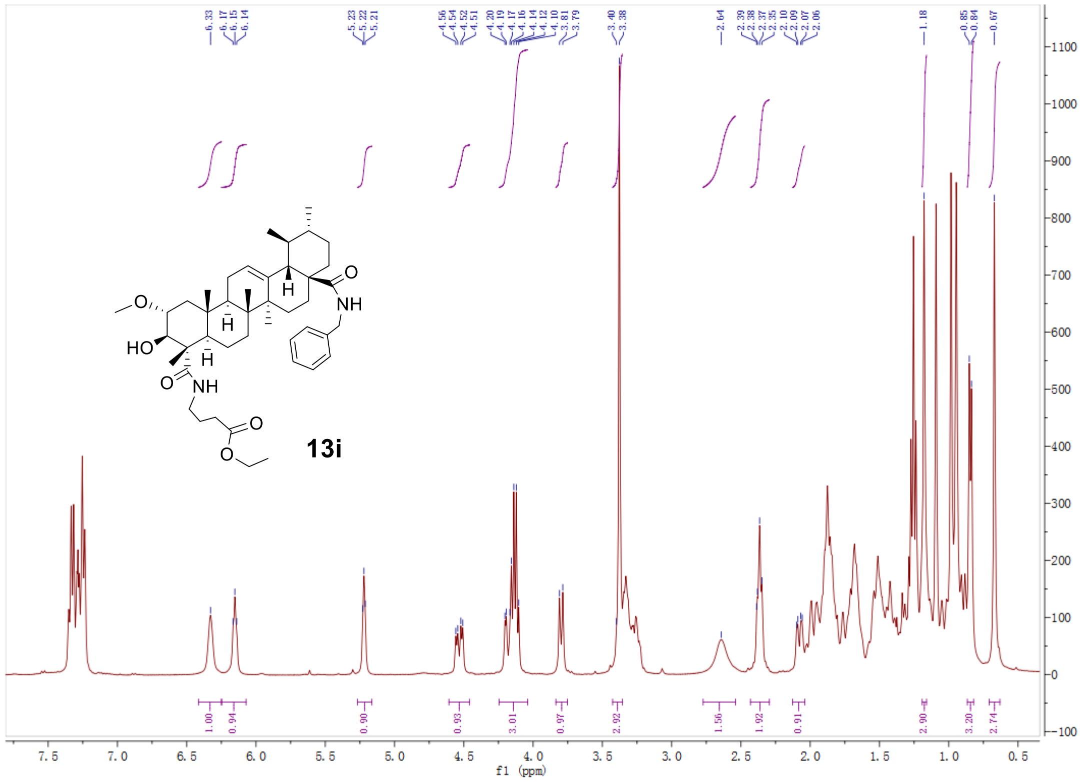

^{1}H NMR Spectrum of N-(2α-methoxy-3β-hydroxy-urs-12-ene-28-benzylamide-23-oyl)- 4-aminobutyric ethyl ester (**13i**) (400 MHz, CDCl3)

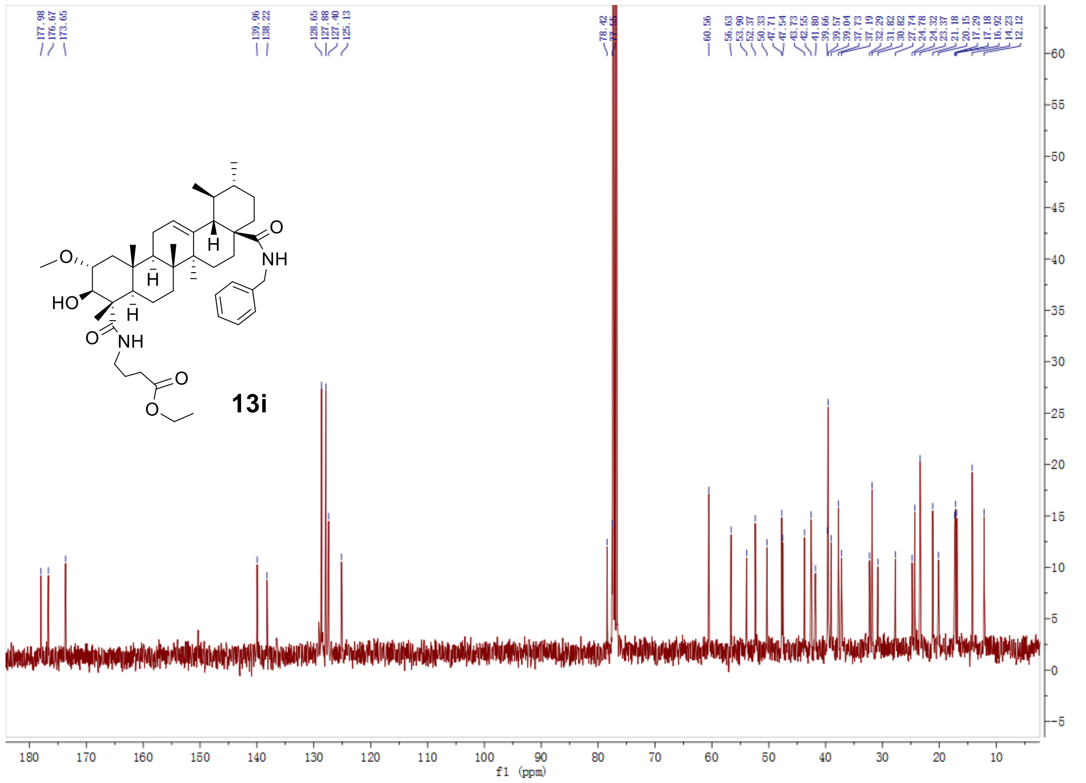

^{13}C NMR Spectrum of N-(2α-methoxy-3β-hydroxy-urs-12-ene-28-benzylamide-23-oyl)- 4-aminobutyric ethyl ester (**13i**) (101 MHz, CDCl3)

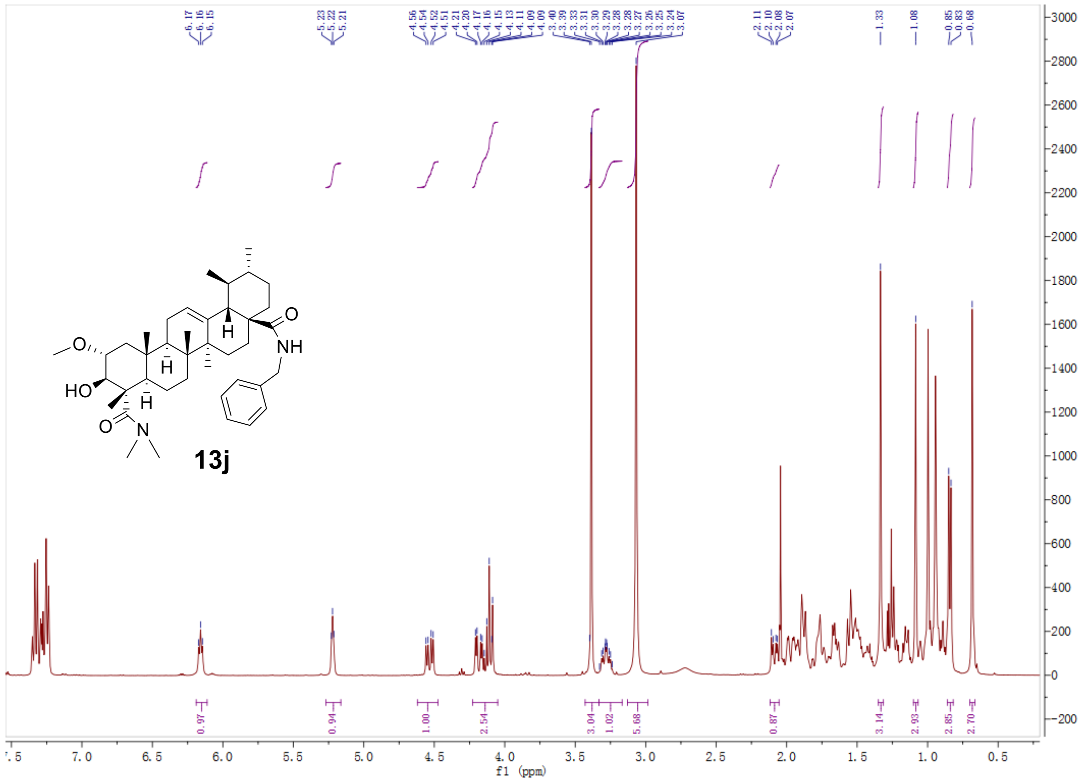

^{1}H NMR Spectrum of 2α-methoxy-3β- hydroxy-urs-12-ene-28-benzyl amide-23-dimethlyamide (**13j**) (400 MHz, CDCl3)

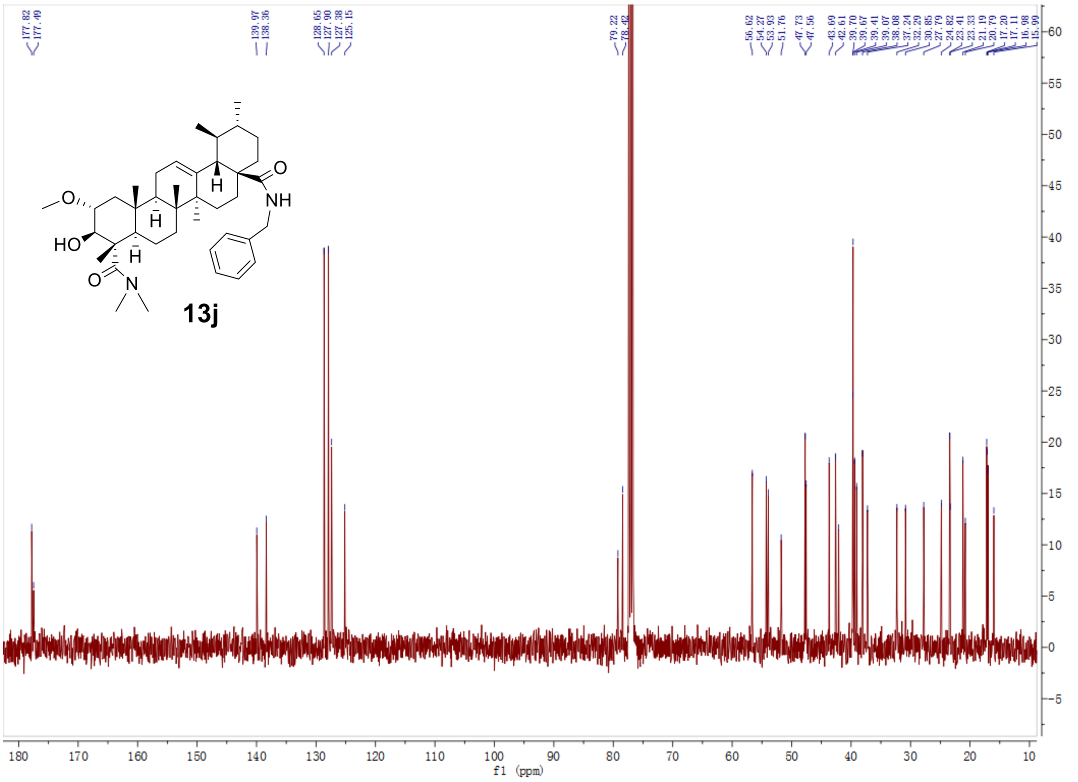

^{13}C NMR Spectrum of 2α-methoxy-3β- hydroxy-urs-12-ene-28-benzyl amide-23-dimethlyamide (**13j**) (101 MHz, CDCl3)

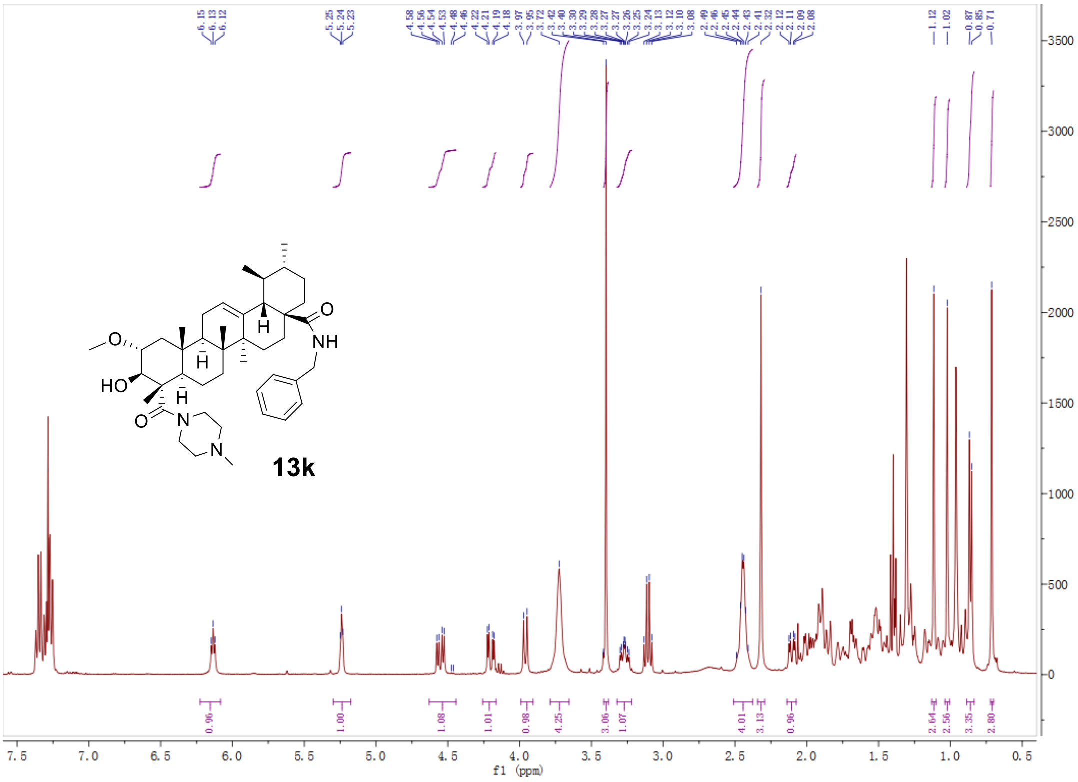

^{1}H NMR Spectrum of 2α-methoxy-3β-hydroxy-urs-12-ene-28-benzylamide-23- (4-methyl-1-piperazinyl)-amide (**13k**) (400 MHz, CDCl3)

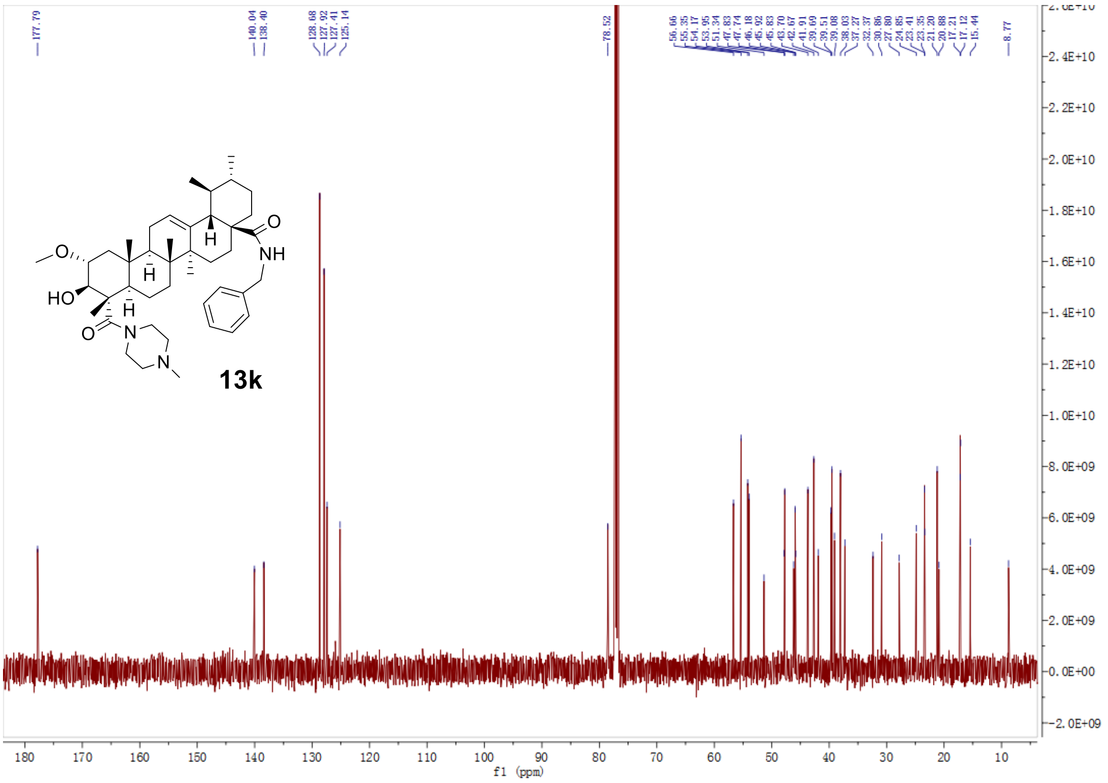

^{13}C NMR Spectrum of 2α-methoxy-3β-hydroxy-urs-12-ene-28-benzylamide-23- (4-methyl-1-piperazinyl)-amide (**13k**) (126 MHz, $CDCl_3$)

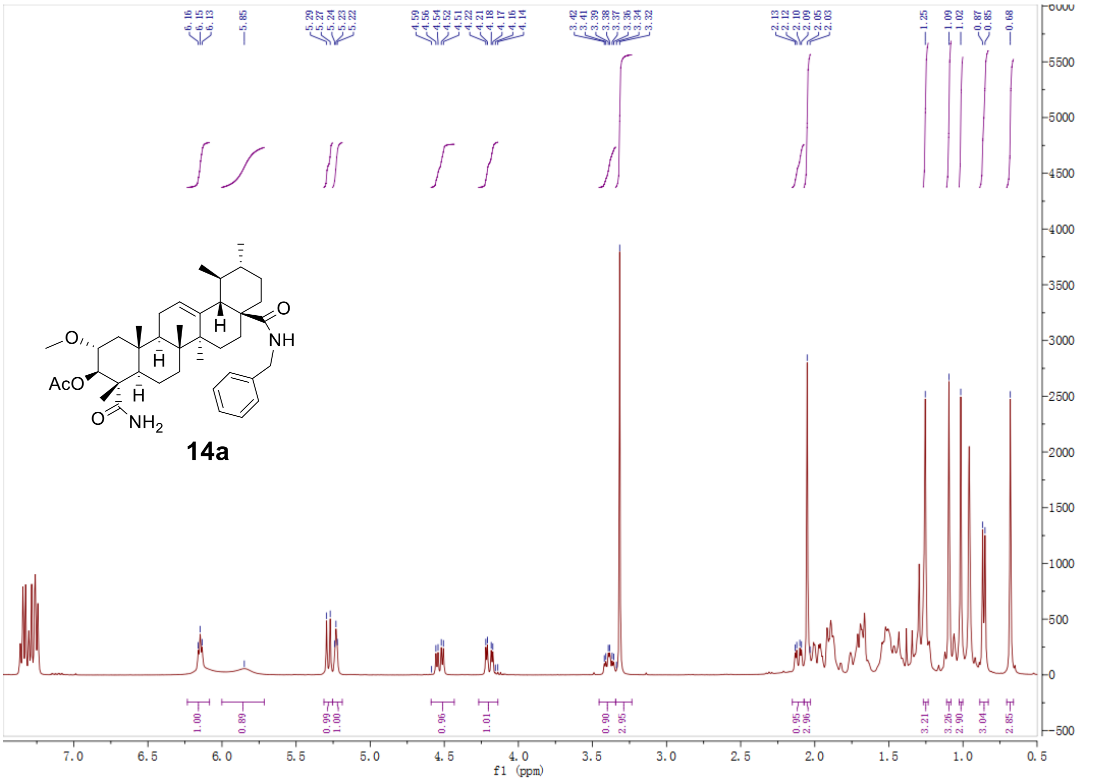

^{1}H NMR Spectrum of 2α-methoxy-3β-acetoxy-urs-12-ene-28-benzyl amide-23-amide (**14a**) (400 MHz, CDCl3)

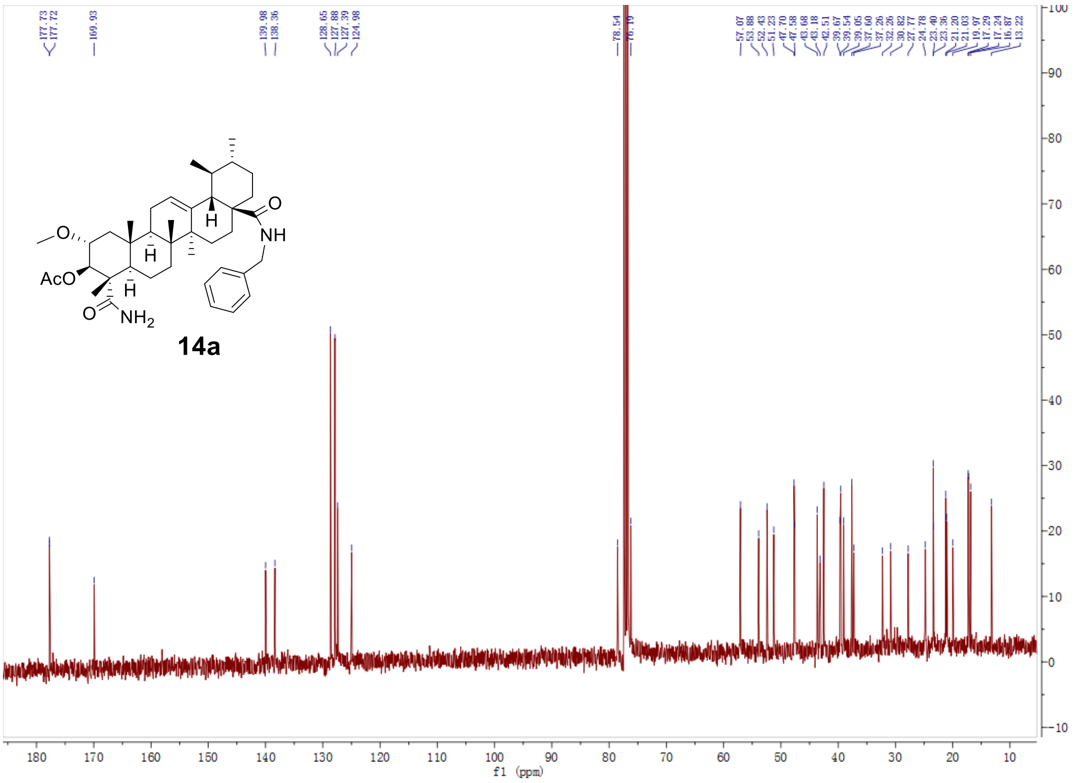

^{13}C NMR Spectrum of α-methoxy-3β-acetoxy-urs-12-ene-28-benzyl amide-23-amide (**14a**) (101 MHz, CDCl3)

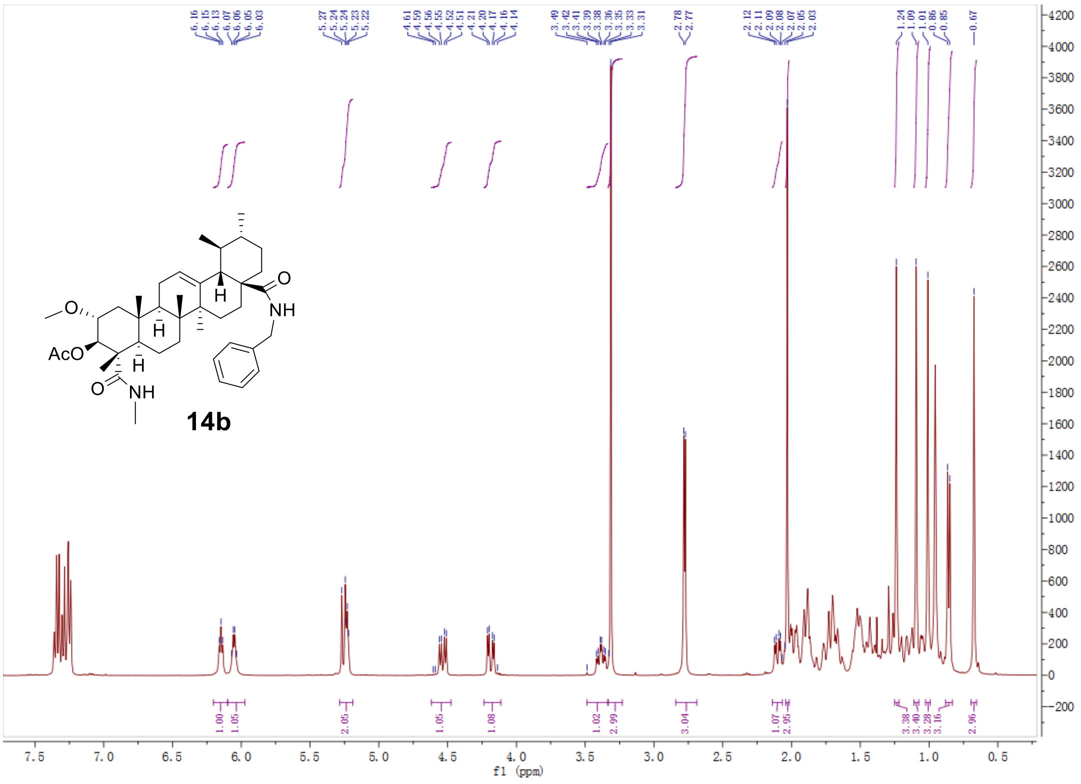

^{1}H NMR Spectrum of 2α-methoxy-3β-acetoxy-urs-12-ene-28-benzyl amide-23-methlyamide (**14b**) (400 MHz, CDCl3)

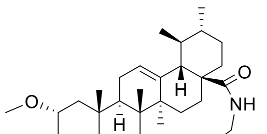

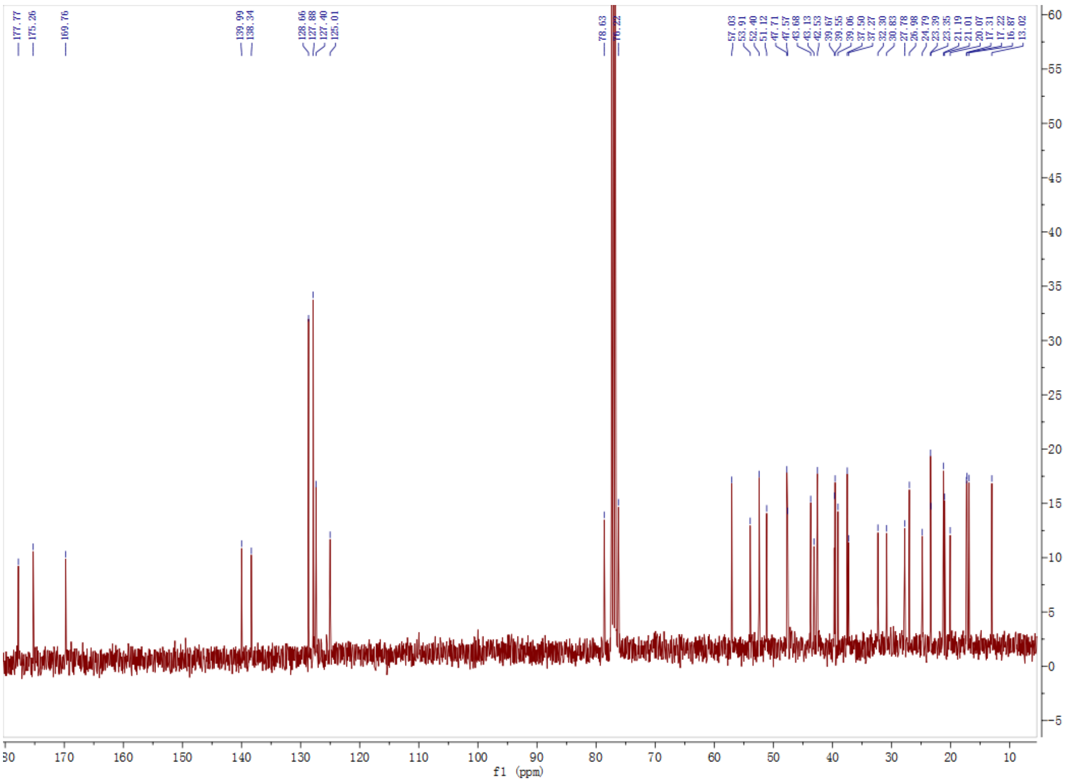

^{13}C NMR Spectrum of 2α-methoxy-3β-acetoxy-urs-12-ene-28-benzyl amide-23-methlyamide (**14b**) (101 MHz, CDCl3)

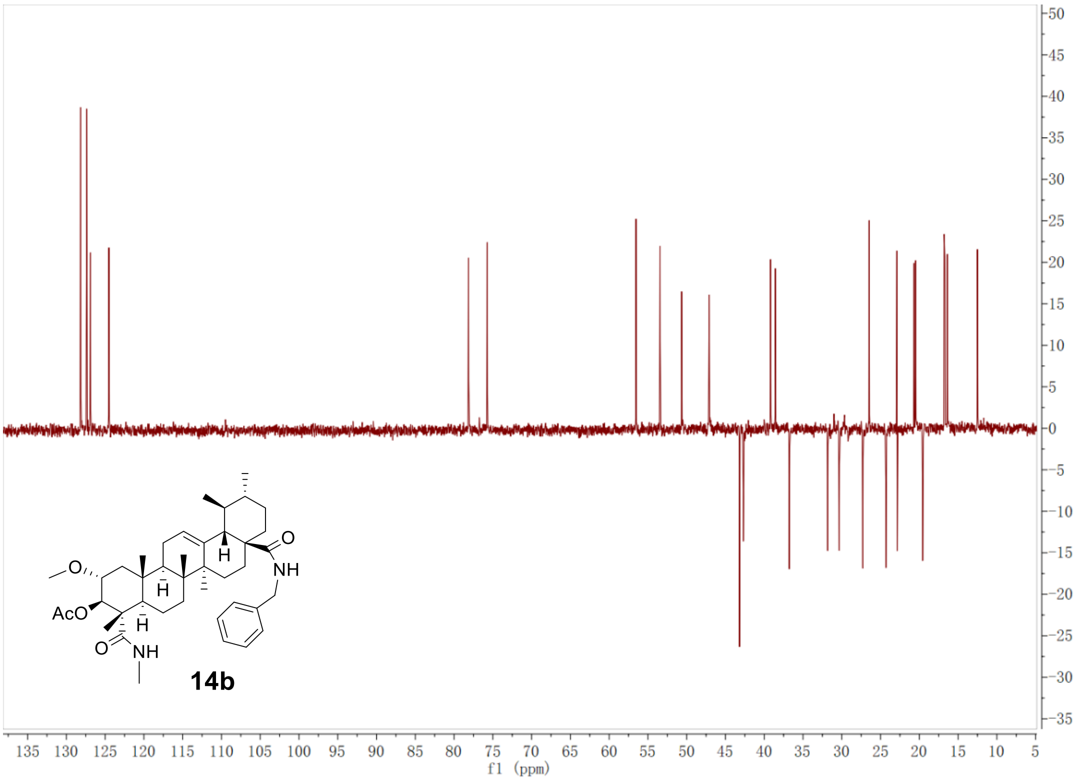

DEPT135 NMR Spectrum of 2α-methoxy-3β-acetoxy-urs-12-ene-28-benzyl amide-23-methlyamide (**14b**) (^{13}C NMR 126 MHz)

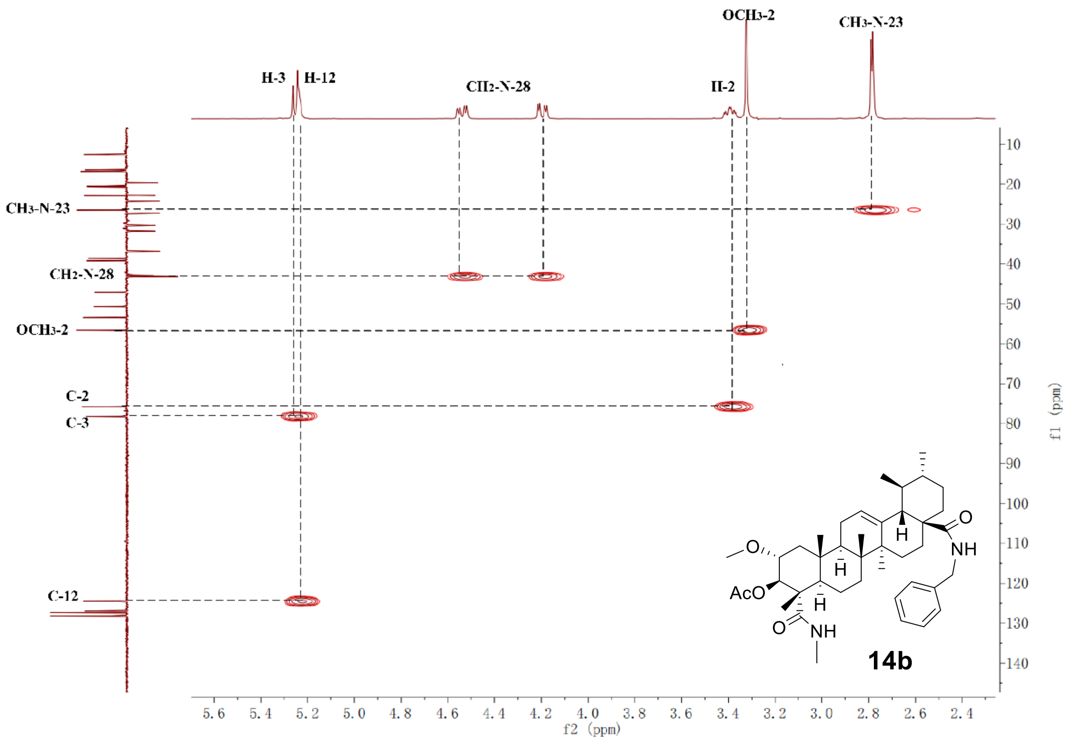

2-D ^{1}H-^{13}C HSQC NMR Spectrum of 2α-methoxy-3β-acetoxy-urs-12-ene-28-benzyl amide-23-methlyamide (**14b**) (^{1}H NMR 500 MHz, $CDCl_3$, ^{13}C NMR 126 MHz, $CDCl_3$)

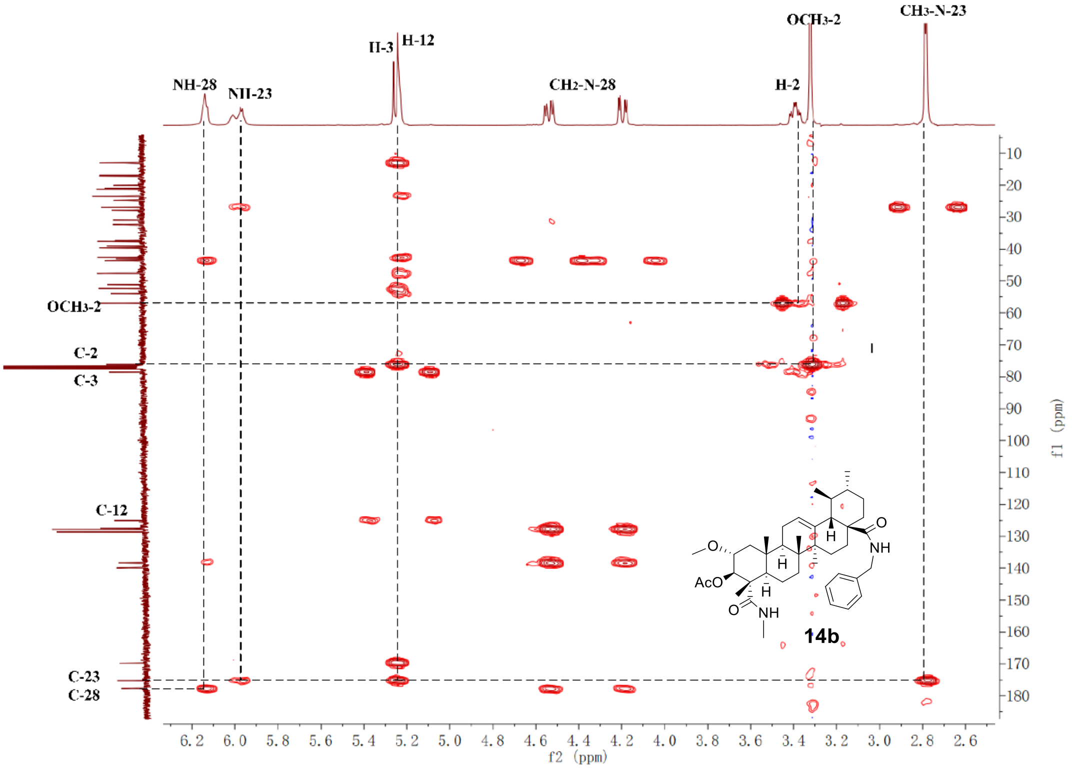

2-D ^{1}H-^{13}C HMBC NMR Spectrum of 2α-methoxy-3β-acetoxy-urs-12-ene-28-benzyl

amide-23-methlyamide (**14b**) (^{1}H NMR 500 MHz, $CDCl_3$, ^{13}C NMR 126 MHz, $CDCl_3$)

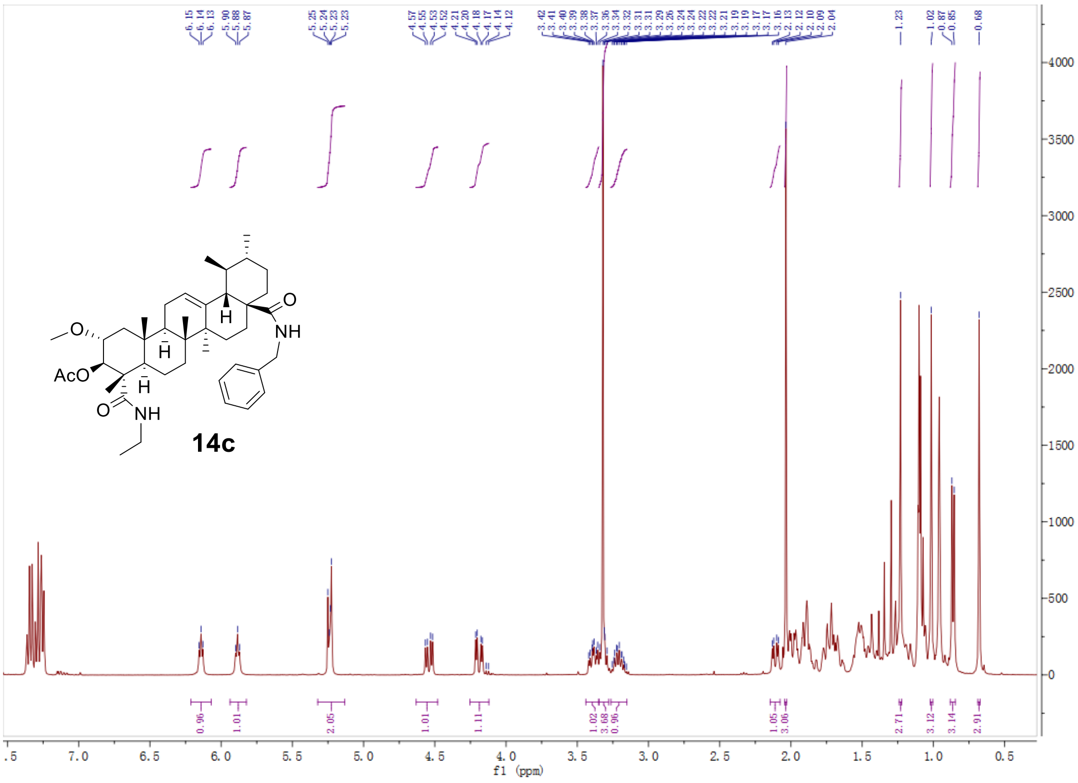

^{1}H NMR Spectrum of 2α-methoxy-3β-acetoxy-urs-12-ene-28-benzyl amide-23-

ethlyamide (**14c**) (400 MHz, CDCl3)

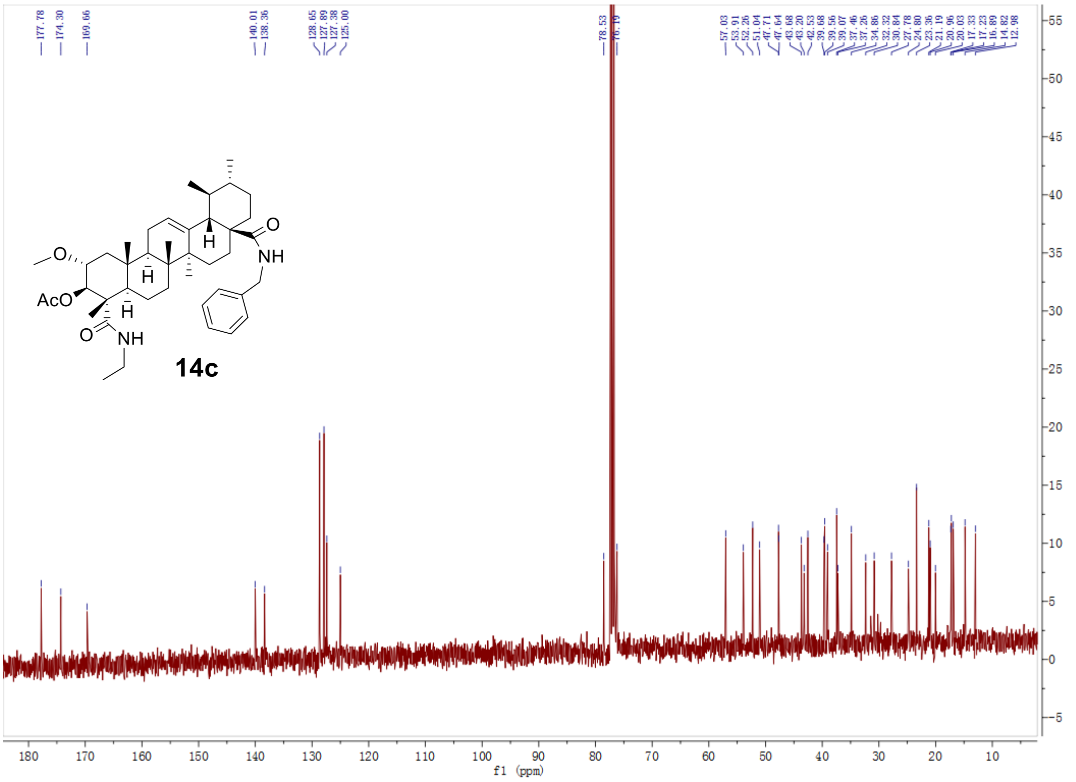

^{13}C NMR Spectrum of 2α-methoxy-3β-acetoxy-urs-12-ene-28-benzyl amide-23-ethlyamide (**14c**) (101 MHz, CDCl3)

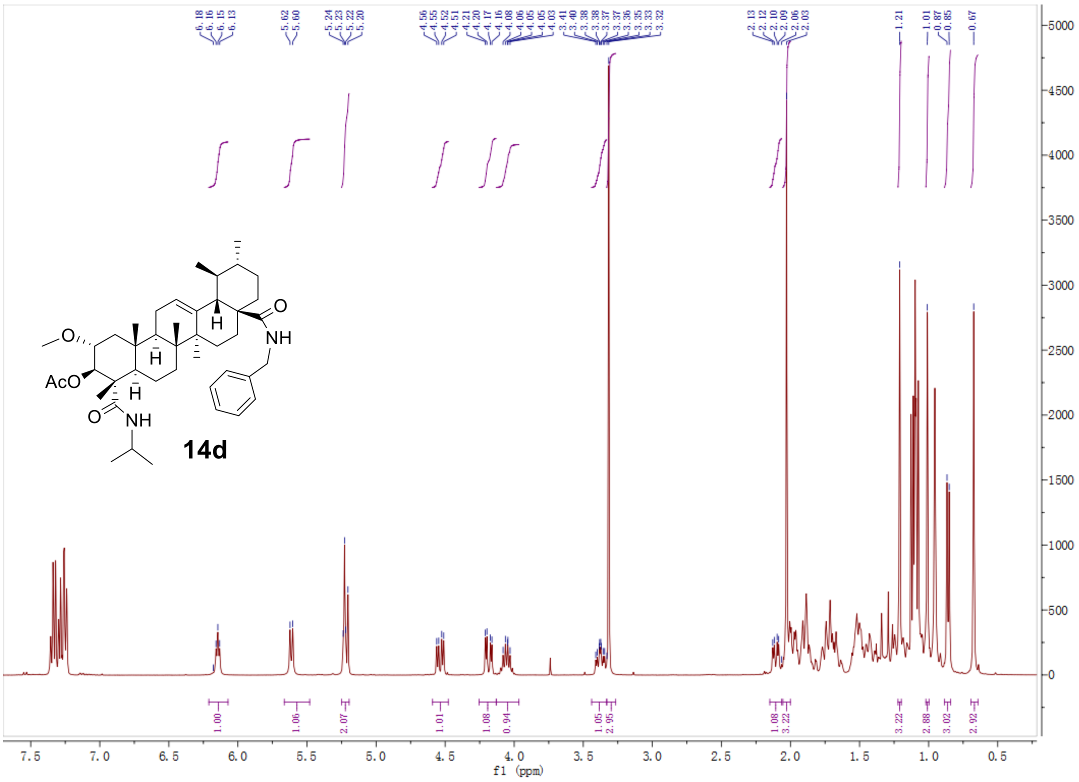

^{1}H NMR Spectrum of 2α-methoxy-3β-acetoxy-urs-12-ene-28-benzyl amide-23-isopropylamide (**14d**) (400 MHz, CDCl3)

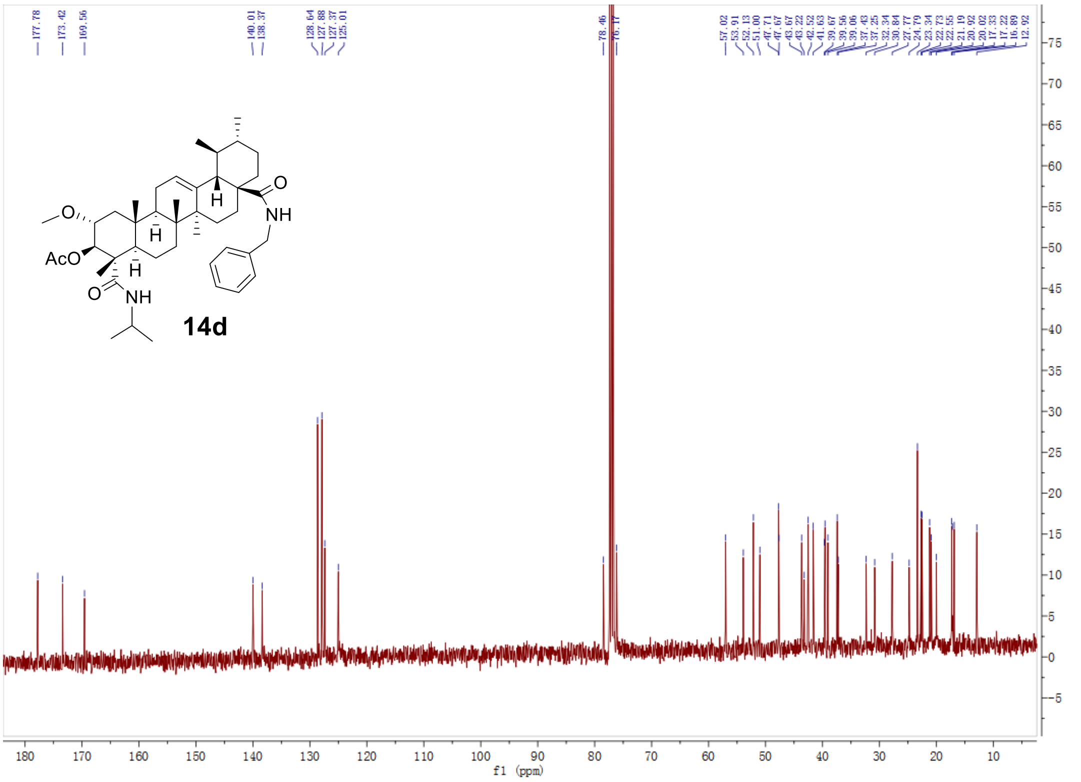

^{13}C NMR Spectrum of 2α-methoxy-3β-acetoxy-urs-12-ene-28-benzyl amide-23-isopropylamide (**14d**) (101 MHz, CDCl3)

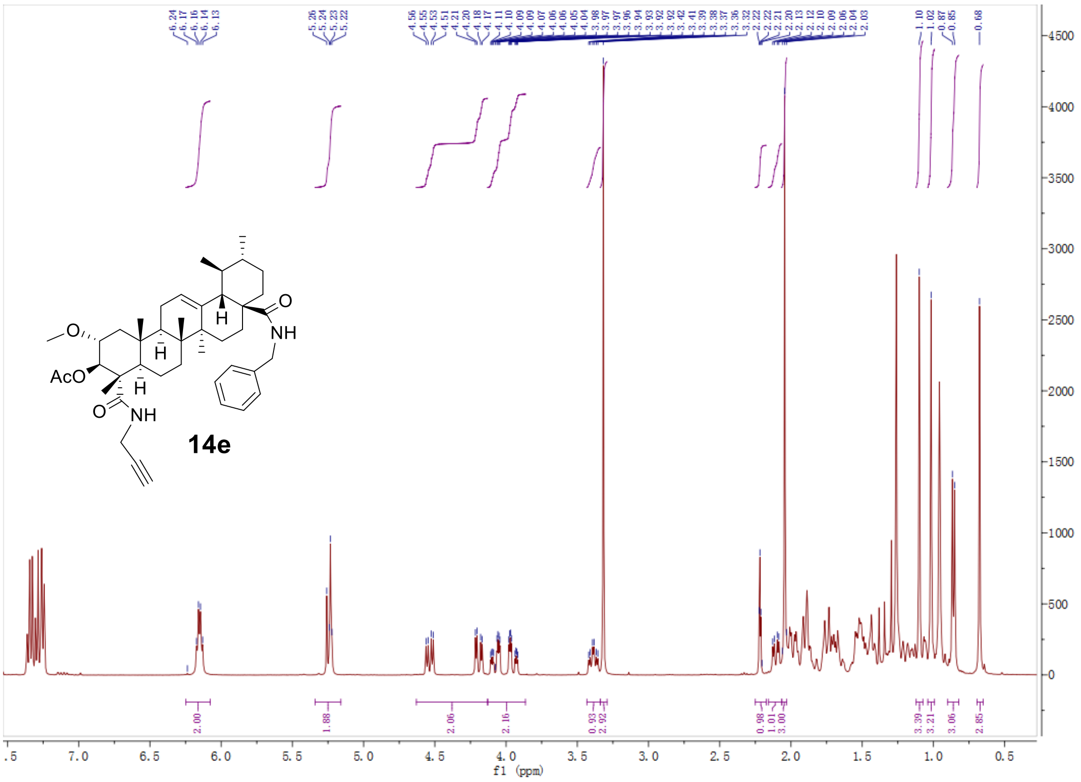

^{1}H NMR Spectrum of 2α-methoxy-3β-acetoxy-urs-12-ene-28-benzyl amide-23-propargylamide (**14e**) (400 MHz, CDCl3)

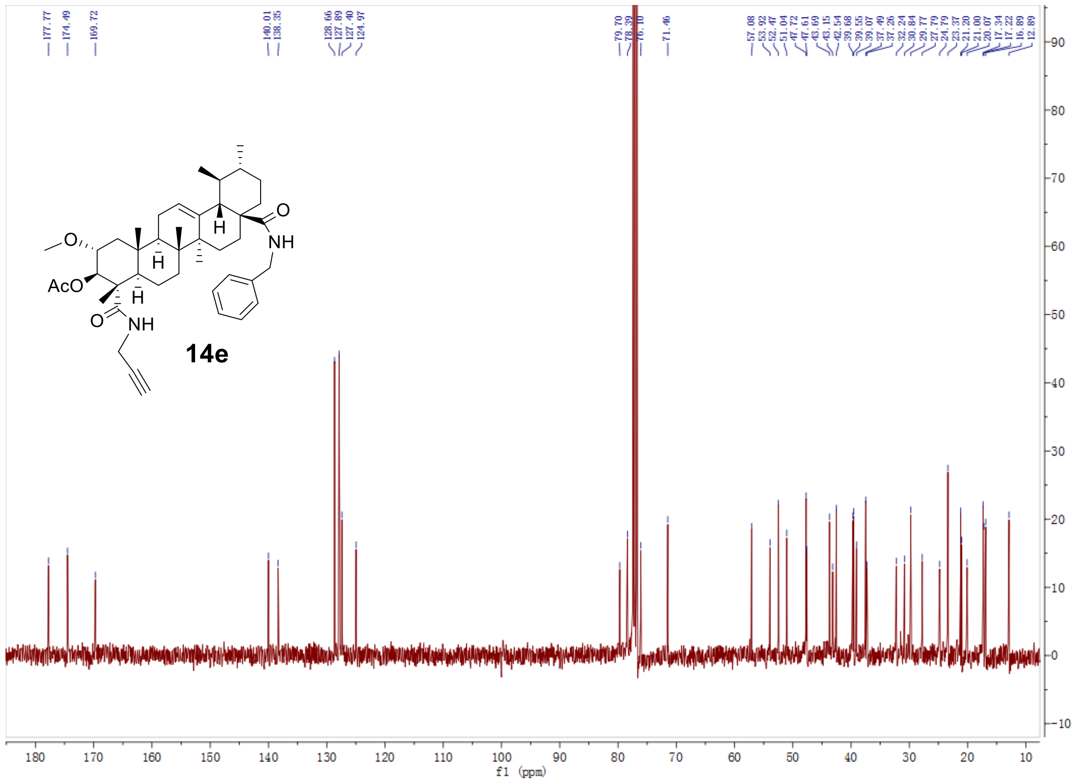

^{13}C NMR Spectrum of 2α-methoxy-3β-acetoxy-urs-12-ene-28-benzyl amide-23-propargylamide (**14e**) (101 MHz, CDCl3)

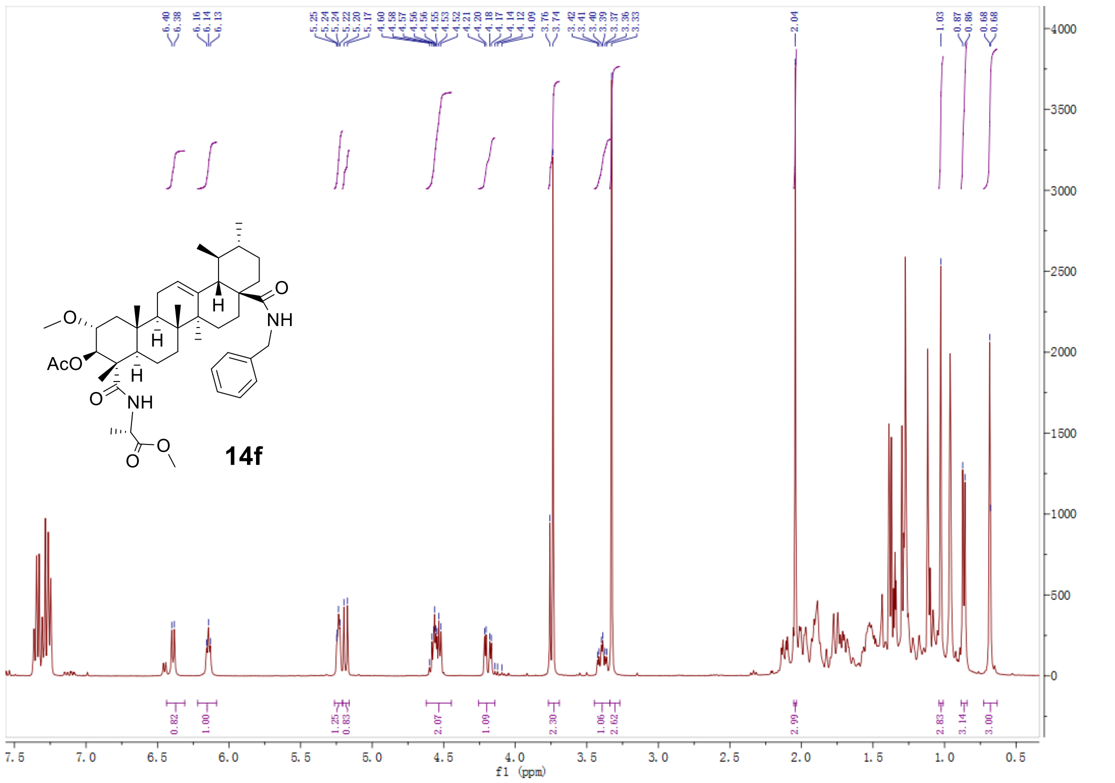

^{1}H NMR Spectrum of N-(2α-methoxy-3β-acetoxy-urs-12-ene-28-benzylamide-23-oyl)-L-alanine methyl ester (**14f**) (400 MHz, CDCl3)

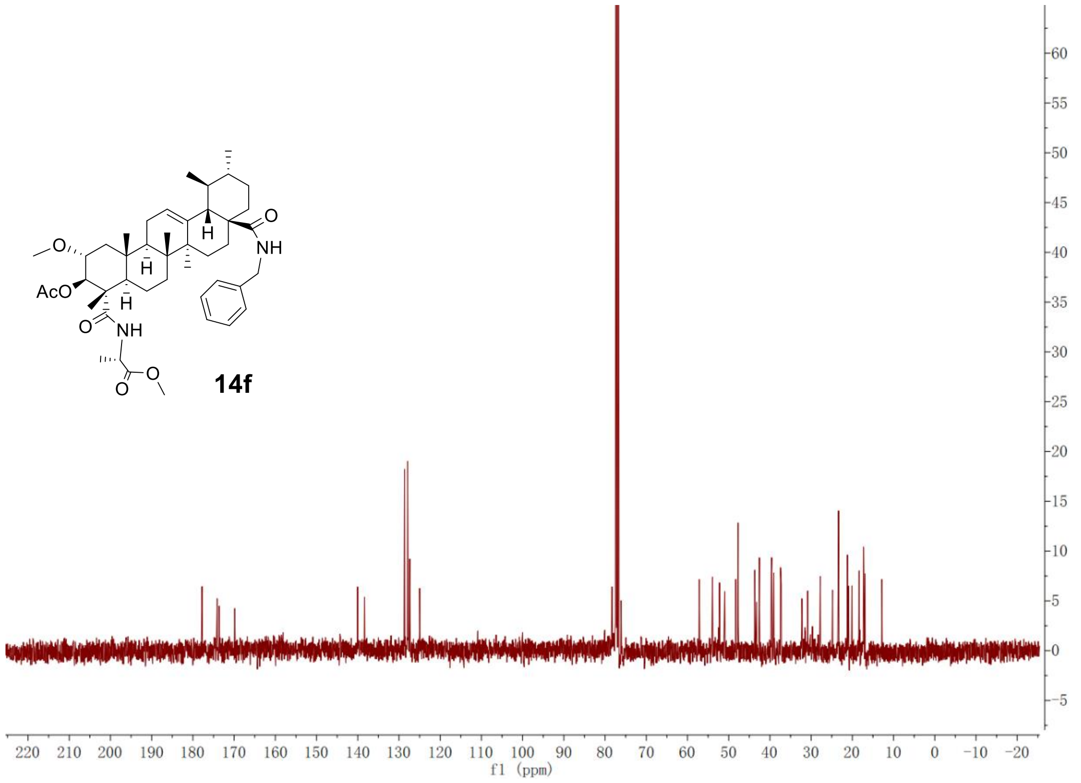

^{13}C NMR Spectrum of N-(2α-methoxy-3β-acetoxy-urs-12-ene-28-benzylamide-23-oyl)-L-alanine methyl ester (**14f**) (101 MHz, CDCl3)

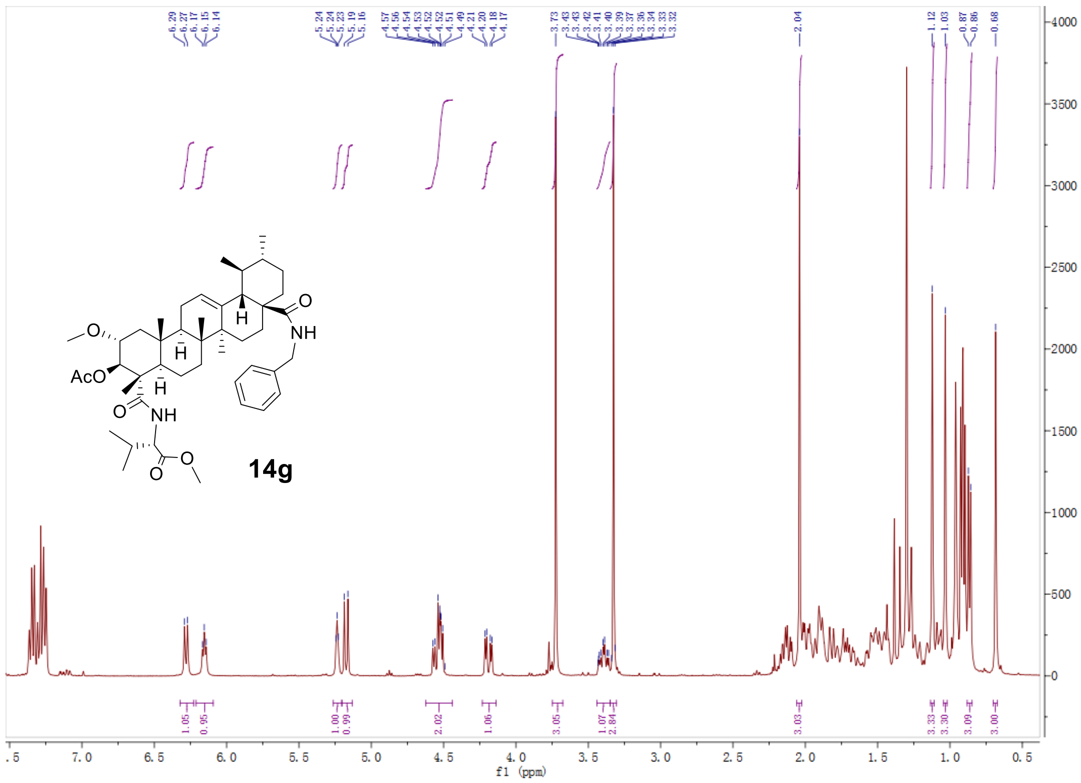

^{1}H NMR Spectrum of N-(2α-methoxy-3β-acetoxy-urs-12-ene-28-benzylamide-23-oyl)-L-valine methyl ester (**14g**) (400 MHz, CDCl3)

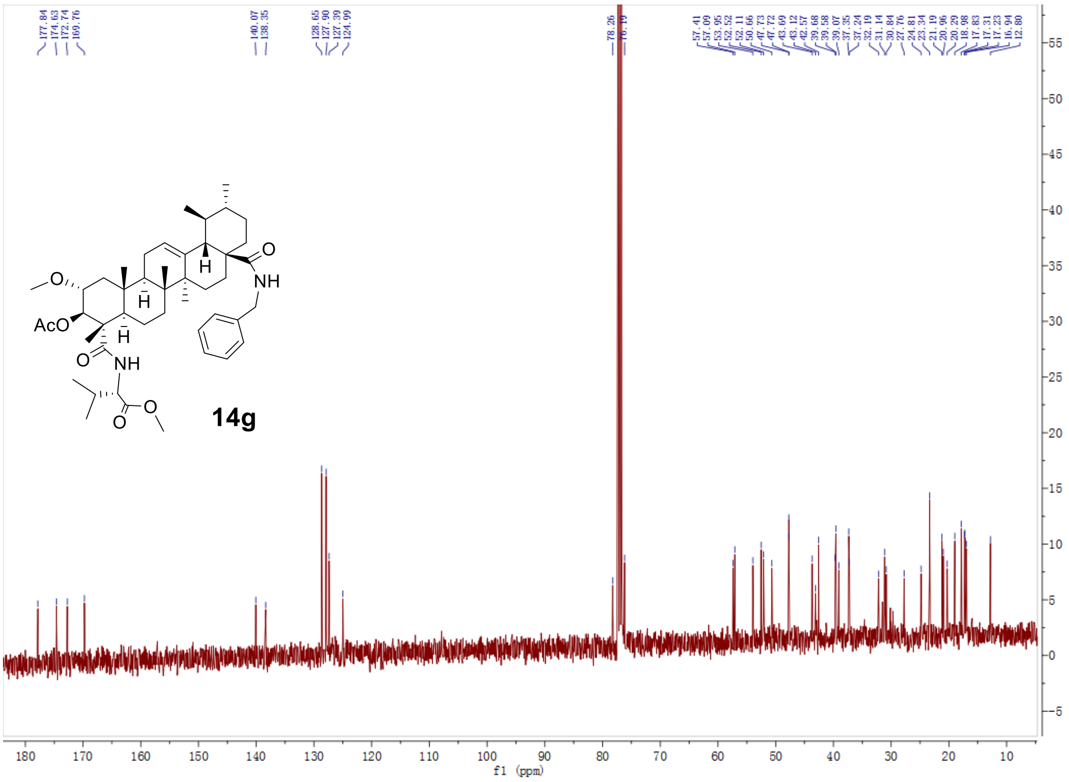

[13] C NMR Spectrum of N-(2α-methoxy-3β-acetoxy-urs-12-ene-28-benzylamide-23-oyl)-L-valine methyl ester (**14g**) (101 MHz, CDCl3)

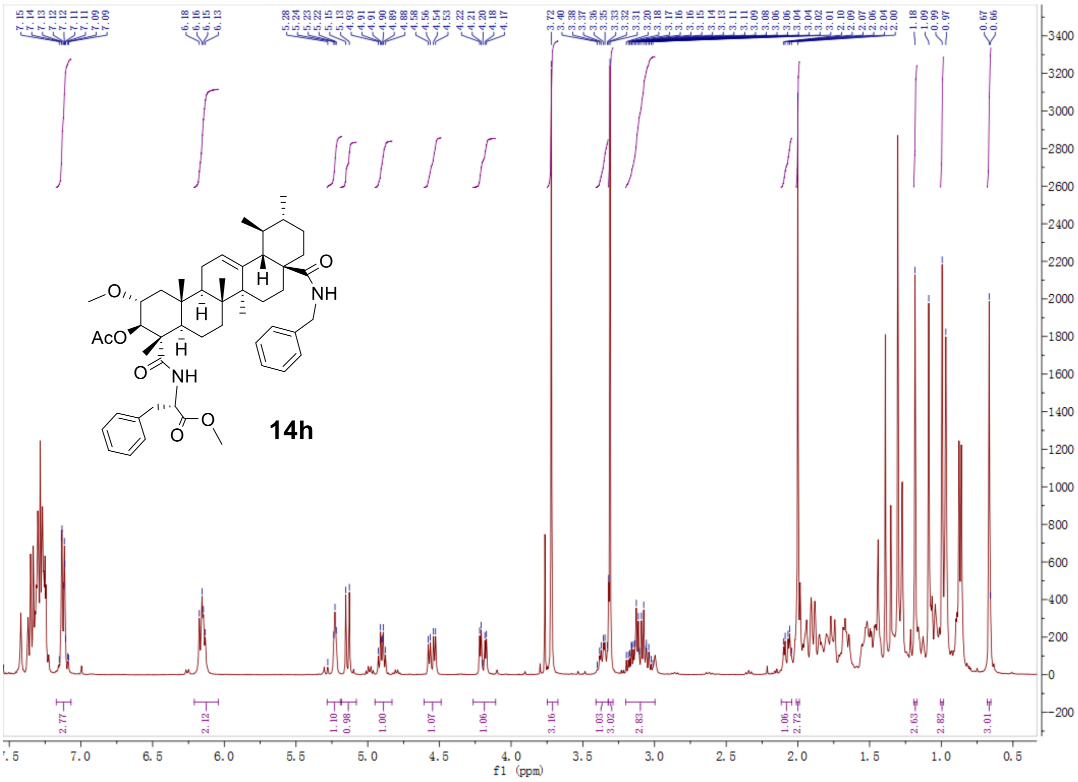

[1] H NMR Spectrum of N-(2α-methoxy-3β-acetoxy-urs-12-ene-28-benzylamide-23-oyl)- L-phenylalanine methyl ester (**14h**) (400 MHz, CDCl3)

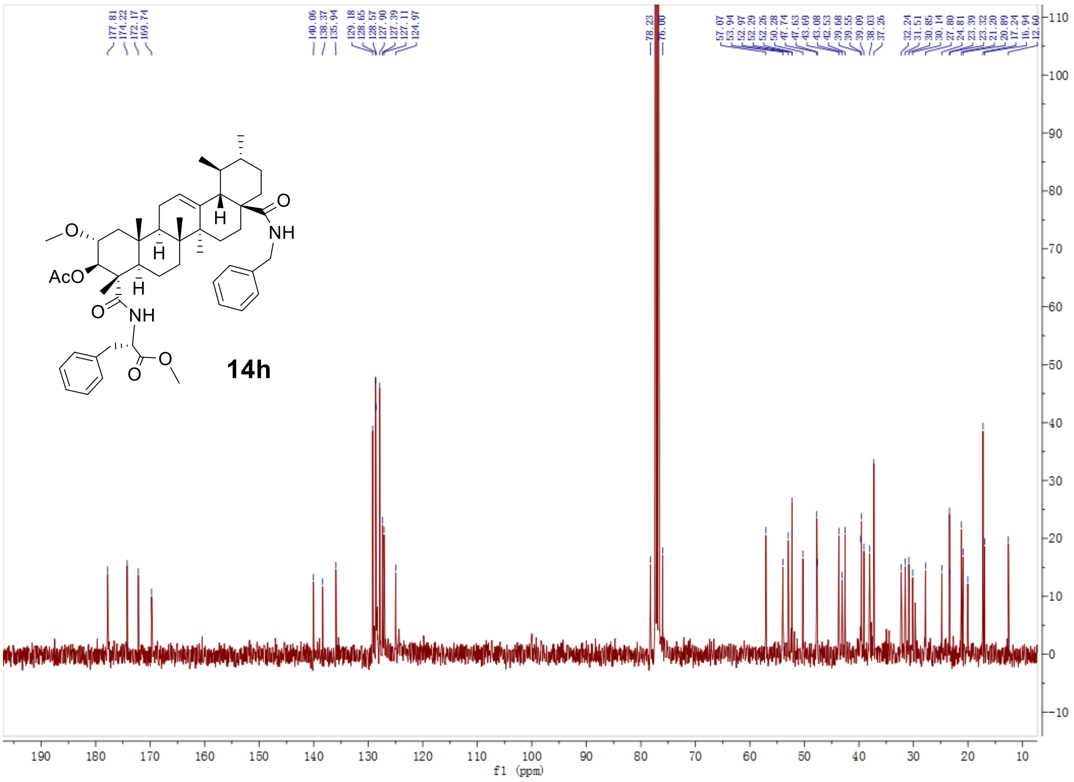

^{13}C NMR Spectrum of N-(2α-methoxy-3β-acetoxy-urs-12-ene-28-benzylamide-23-oyl)- L-phenylalanine methyl ester (**14h**) (101 MHz, CDCl3)

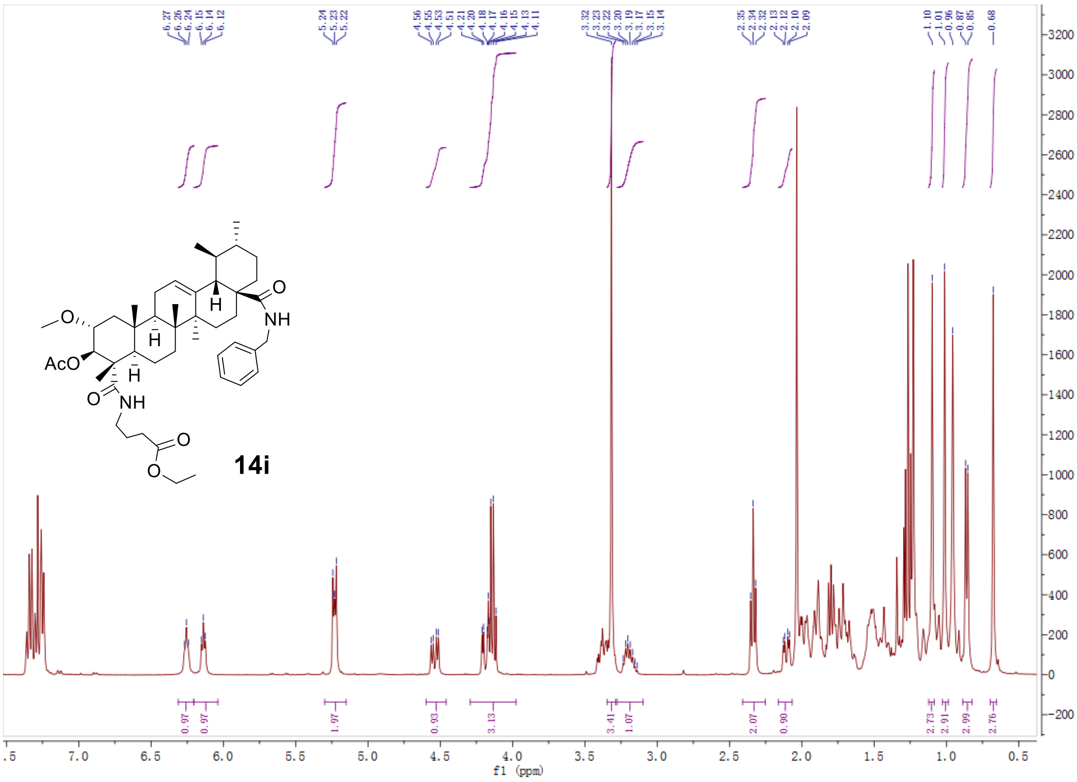

^{1}H NMR Spectrum of N-(2α-methoxy-3β-acetoxy-urs-12-ene-28-benzylamide-23-oyl)-4-aminobutyric ethyl ester (**14i**) (400 MHz, CDCl3)

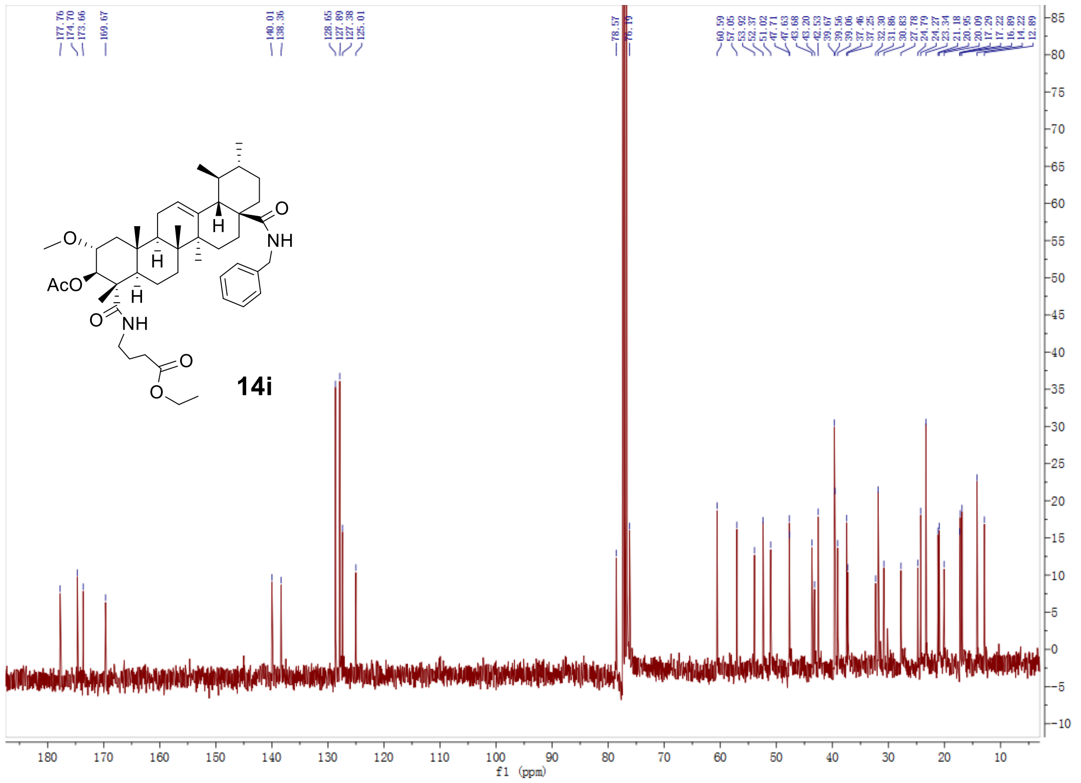

^{13}C NMR Spectrum of N-(2α-methoxy-3β-acetoxy-urs-12-ene-28-benzylamide-23-oyl)-4-aminobutyric ethyl ester (**14i**) (101 MHz, CDCl3)

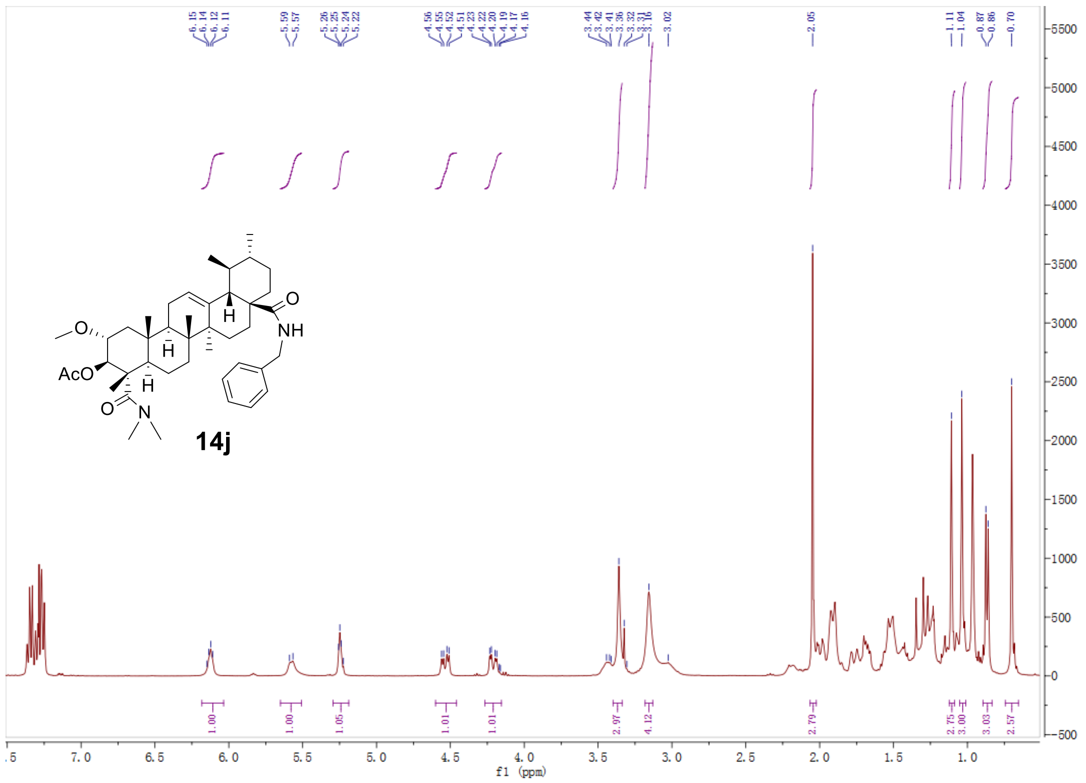

^{1}H NMR Spectrum of 2α-methoxy-3β- acetoxy-urs-12-ene-28-benzyl amide-23-dimethlyamide (**14j**) (400 MHz, CDCl3)

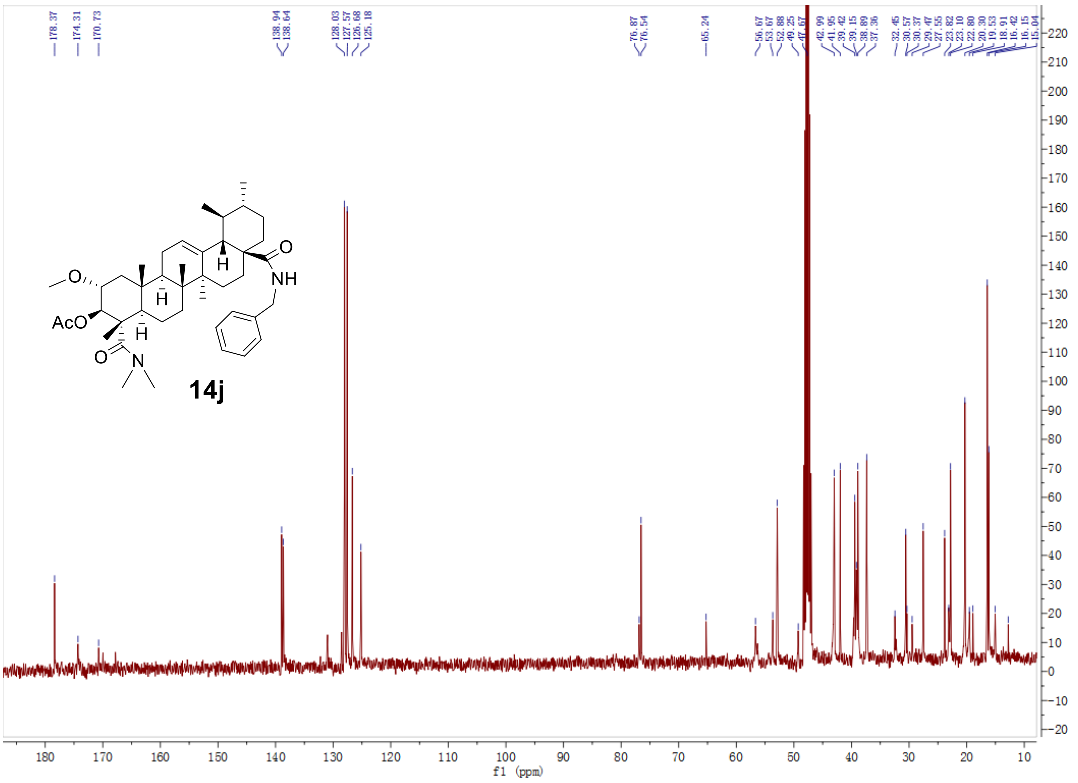

^{13}C NMR Spectrum of 2α-methoxy-3β- acetoxy-urs-12-ene-28-benzyl amide-23-dimethlyamide (**14j**) (101 MHz, MeOD)

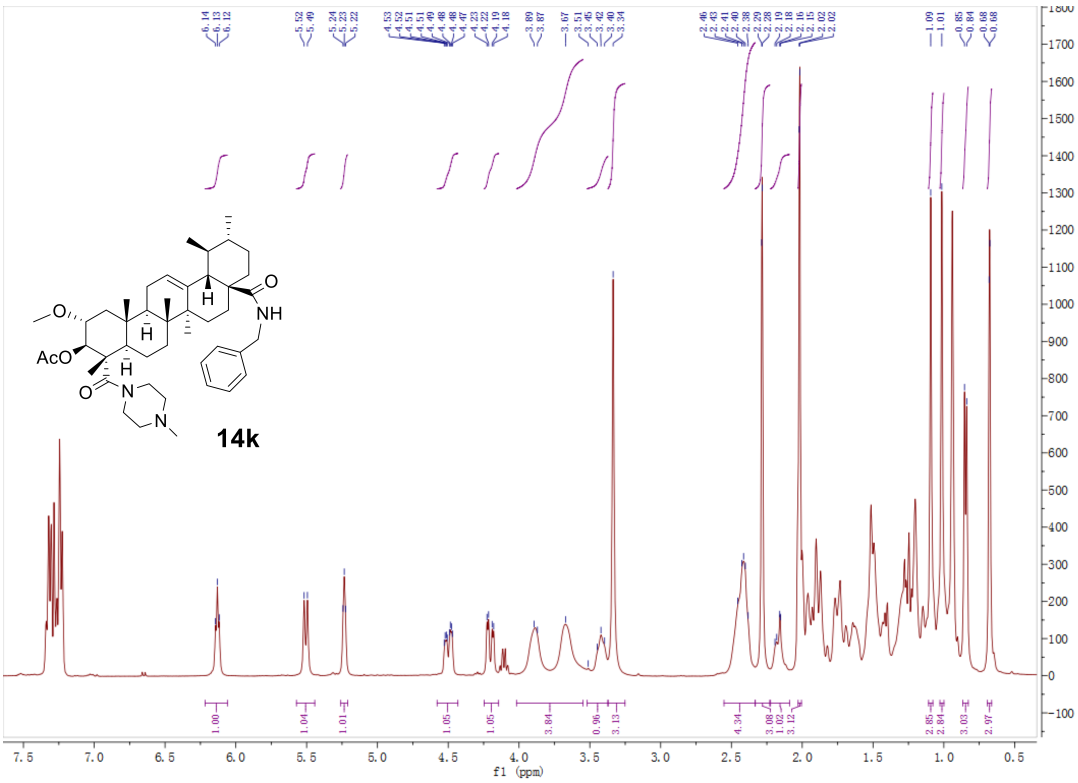

^{1}H NMR Spectrum of 2α-methoxy-3β-acetoxy-urs-12-ene-28-benzylamide-23-(4-methyl-1-piperazinyl)-amide (**14k**) (400 MHz, CDCl3)

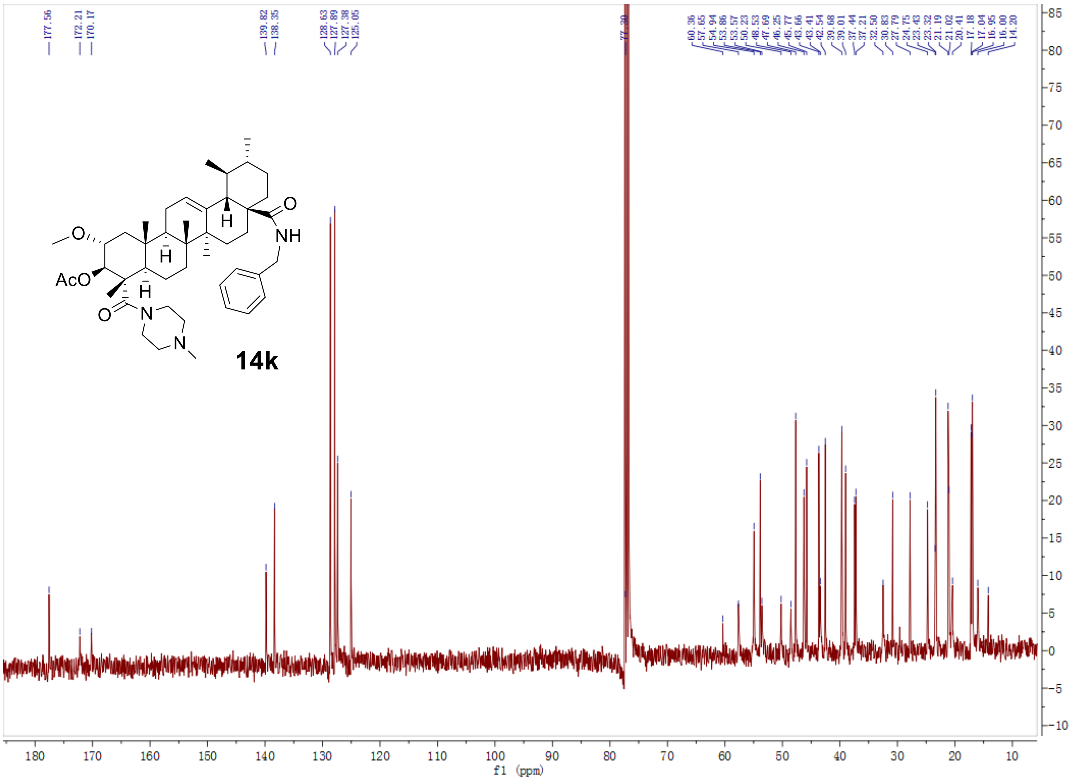

^{13}C NMR Spectrum of 2α-methoxy-3β-acetoxy-urs-12-ene-28-benzylamide-23-(4-methyl-1-piperazinyl)-amide (**14k**) (101 MHz, CDCl3)

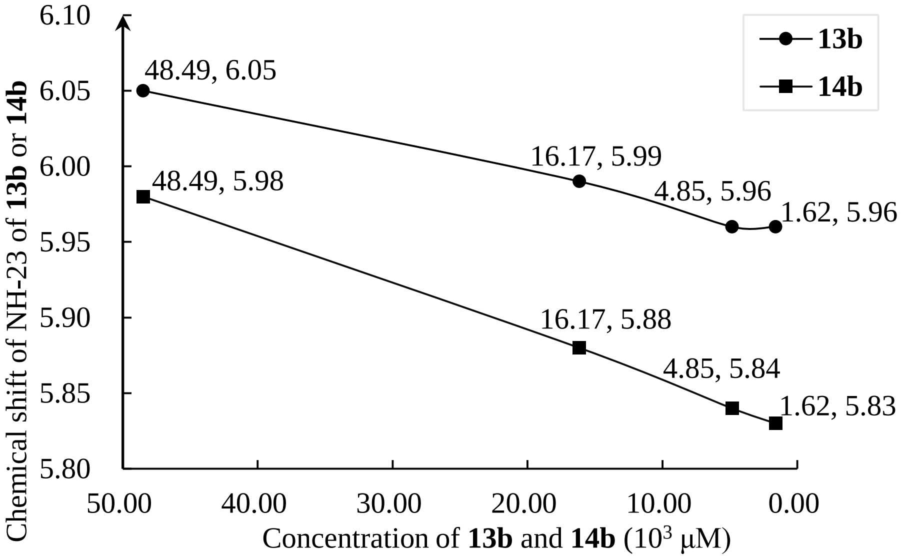

Fig. 1. NMR titration curves for compounds **13b** and **14b**.

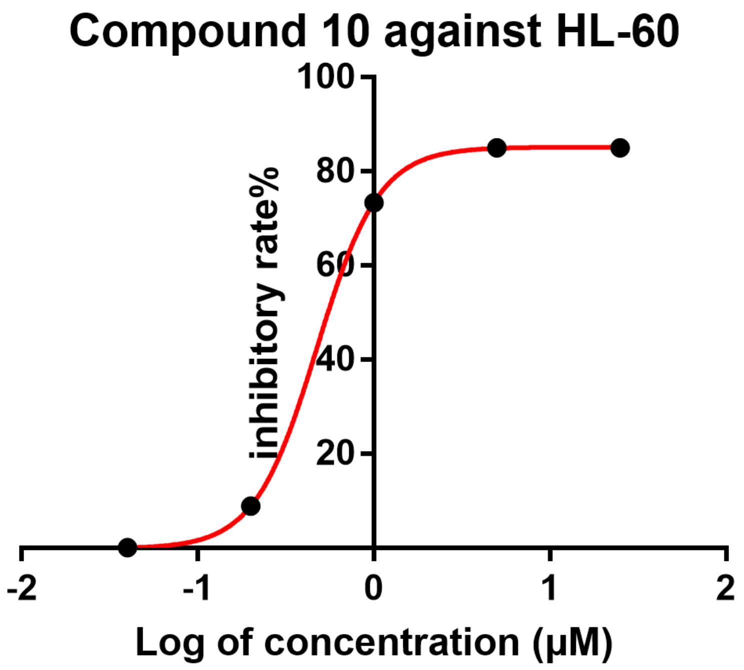

Fig. 2. IC_{50} curve of compound **10** against HL-60.

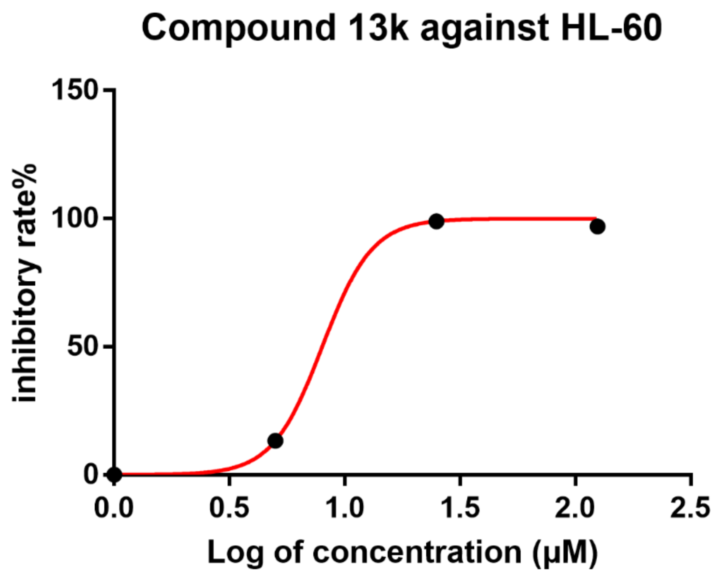

Fig. 3. IC_{50} curve of compound **13k** against HL-60.

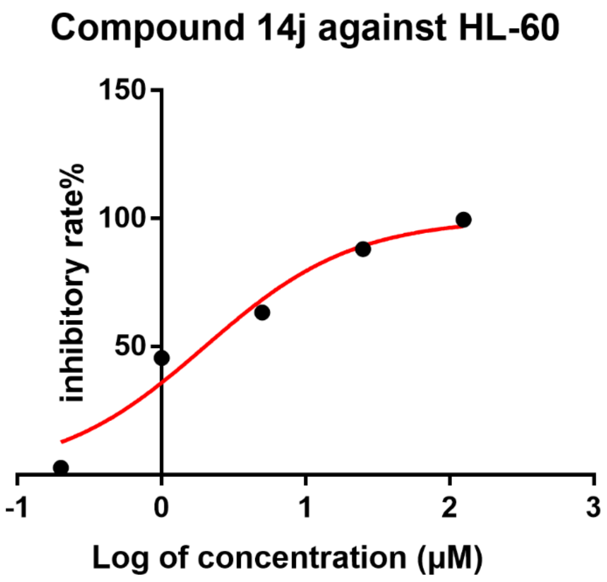

Fig. 4. IC_{50} curve of compound **14j** against HL-60.

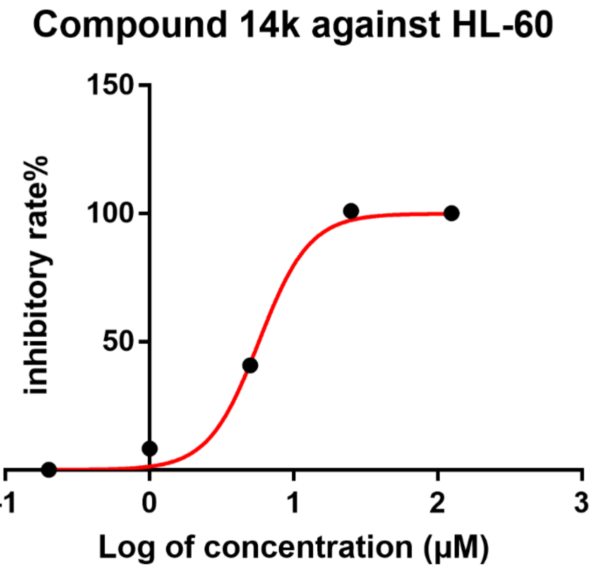

Fig. 5. IC_{50} curve of compound **14k** against HL-60.
